# Tuning the ^2^LMCT Deactivation of Cyclometalated Iron Carbene Complexes with Electronic Substituent Effects

**DOI:** 10.1002/chem.202501985

**Published:** 2025-07-25

**Authors:** Abhishek Mishra, Kumkum Sharma, Catherine Ellen Johnson, Emmanuel Adu Fosu, Jesper Schwarz, Om Prakash, Arvind Kumar Gupta, Ping Huang, Fredrik Lindgren, Lennart Häggström, Jesper Bendix, Elena Jakubikova, Reiner Lomoth, Kenneth Wärnmark

**Affiliations:** ^1^ Centre for Analysis and Synthesis Department of Chemistry Lund University Box 124 SE‐22100 Lund Sweden; ^2^ Department of Chemistry – Ångström Laboratory Uppsala University Box 523 SE‐75120 Uppsala Sweden; ^3^ Emmanuel Adu Fosu Elena Jakubikova Department of Chemistry North Carolina State University Raleigh NC 27695 USA; ^4^ Department of Physics – Ångström Laboratory Uppsala University Box 538 SE‐75121 Uppsala Sweden; ^5^ Department of Chemistry University of Copenhagen Universitetsparken 5 DK‐2100 Copenhagen Denmark

**Keywords:** iron, N‐heterocyclic carbene, photophysics, photoredox catalysis, Potential energy diagrams

## Abstract

Fe^III^ complexes based on the [Fe^III^(ImP)_2_]^+^ motif (ImP = bis(2,6‐bis(3‐methylimidazol‐2‐ylidene‐1‐yl)phenylene)), where the ligand contains both carbene and cyclometalated moieties, are a promising class of photoactive materials made from this abundant metal. In this work, it is shown that bromo or furanyl substituents attached to the cyclometalating moiety of the ImP ligands stabilize the ^2^LMCT excited state to very different extent resulting in opposing effects on the ^2^LMCT lifetime. For [Fe^III^(ImPBr)_2_]^+^, the lifetime (255 ps) of its moderately stabilized ^2^LMCT state (1.85 eV) is slightly increased compared to the parent complex (1.90 eV, 240 ps) pointing to an increased barrier for deactivation via the ^4^MC state and enabling applications as photoredox catalyst. In contrast, the ^2^LMCT energy of [Fe^III^(ImPFur)_2_]^+^ is lowered substantially to a value of 1.63 eV due to the extended π‐system of the ligands and the reduced energy gap favors internal conversion directly to the ground state resulting in a considerably reduced ^2^LMCT lifetime of 59 ps. These findings have general implications for design of ligand modifications aiming at extended LMCT lifetimes and/or modified ground and excited state potentials.

## Graphical abstract

Bromo‐ or furanyl‐substituents on a cyclometalated iron(III) N‐heterocyclic complex stabilize the ^2^LMCT state to very different extent. With substantially different ^2^LMCT energies and lifetimes, the two cases illustrate the balance between non‐radiative deactivation directly to the ground state or via metal‐centered states.

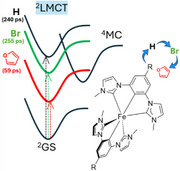

## 1 Introduction

In the pursuit of Earth‐abundant transition metal complexes as photosensitizers and photocatalysts,^[^ ^1^, ^2^, ^3^, ^4^ ^]^ strategies often focus on extending the lifetimes of charge‐transfer (CT) states, which exhibit stronger and broader absorption than metal‐centered (MC) states.^[^ ^5^ ^]^ CT states facilitate efficient use of absorbed light energy, but for the more abundant first row transition metals (TMs),^[^ ^6^, ^7^, ^8^ ^]^ lifetimes are often limited by deactivation through MC states, which possess weaker absorption and act as deactivation pathways. Recent approaches aim to raise MC state energies relative to CT states through strategies such as incorporating strongly σ‐donating or π‐accepting ligands and increasing molecular rigidity.^[^ ^9^, ^10^, ^11^ ^]^

Iron, Earth's most abundant transition metal, has emerged as a promising candidate due to its availability and it being the first‐row congener of ruthenium, however the ^3^MLCT excited state lifetimes of archetypical Fe^II^ polypyridyl complexes are extremely short. A key breakthrough in extending CT state lifetimes has been by the incorporation of strongly σ‐donating *N*‐heterocyclic carbene (NHC) ligands around the iron center,^[^ ^8^, ^12^ ^]^ which has produced iron complexes with nanosecond excited‐state lifetimes, in the Fe^III^ state,^[^ ^13^ ^]^ and a half‐nanosecond lifetime in the Fe^II^ state,^[^ ^14^ ^]^ and made the CT transfer state participate in bimolecular quenching reactions.^[^ ^13^ ^]^ Next to the original *all*‐FeNHC ligand motifs, similar progress has been made with Fe^III^ complexes bearing mixed NHC/cyclometalated ligands.^[^ ^15^, ^16^, ^17^ ^]^ For Fe^II^ complexes with mixed NHC/pyridyl ligand sets, the attachment of electron withdrawing and π‐accepting groups has been previously established as an effective approach to lower ^3^MLCT state energies which disfavors deactivation via MC states and leads to improved ^3^MLCT lifetimes in complexes based on the [Fe^II^(pbmi)_2_]^2+^ motif (pbmi = 1,1′‐(pyridine‐2,6‐diyl)bis(methylimidazole‐2‐ylidine).^[^ ^18^, ^19^, ^20^ ^]^ We were therefore interested to see if analogous effects can be used to tune the ^2^LMCT properties of Fe(III) complexes bearing mixed NHC/cyclometalated ligands.

To explore this concept, bromine or furanyl groups were attached to the *para*‐position of the cyclometalated phenylene ring in complexes based on the established [Fe^III^(ImP)_2_]^+^ motif^[^ ^15^, ^16^ ^]^ that shares a very similar ligand backbone with [Fe^II^(pbmi)_2_]^2+^, except for the substitution of a pyridine for the central cyclometalated ring. While Br‐ substituents exert a plain electron‐withdrawing inductive effect, the furanyl group combines inductive and mesomeric effects in a less predictable way. The electrochemical properties of the complexes revealed that both types of subtituents ease reduction of the Fe(III) center corresponding to a net electron‐withdrawing effect, while the furanyl group in addition facilitates ligand oxidation due to the extended π‐system. As a result, these substituents introduce a very different extent of ^2^LMCT stabilization that was found to cause opposing effects on the ^2^LMCT lifetimes. These results provide insight into the excited‐state dynamics with a critical balance between deactivation via MC states and direct non‐radiative conversion to the ground state.

## 2 Results and Discussion

**Scheme 1:**
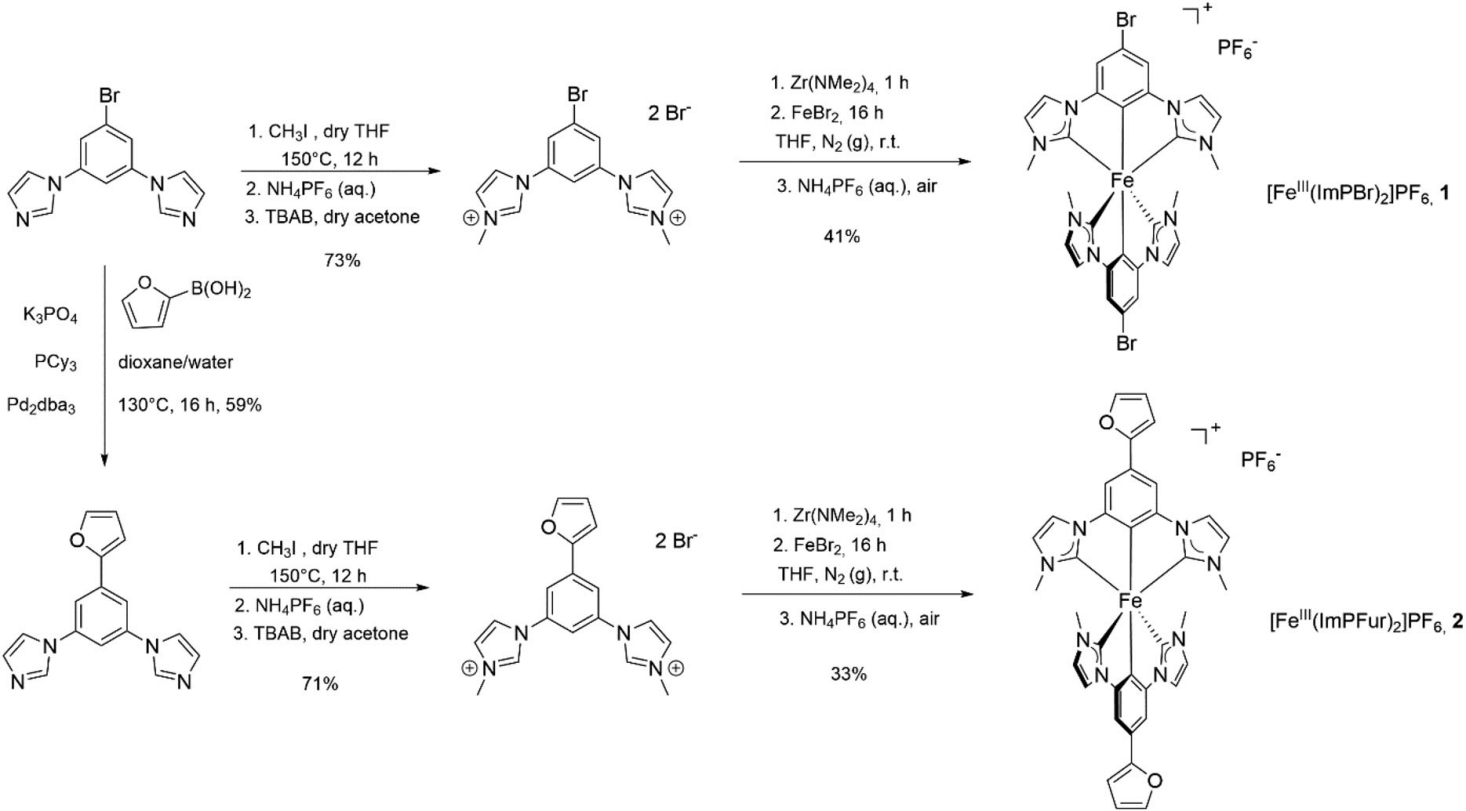
Ligand synthesis and complexation to form [Fe^III^(ImPBr)_2_]PF_6_ and [Fe^III^(ImPFur)_2_]PF_6_.

The synthesis of both ligands started from 1,3,5‐tribromobenzene which was reacted with two equivalents of imidazole under copper catalysis to form 1,1′‐(5‐bromo‐1,3‐phenylene)bis(1*H*‐imidazole) in 45% yield.^[^ ^21^ ^]^ Subsequent methylation with methyl iodide (Scheme 1) gave the corresponding pre‐ligand HImPBr (HImPBr = 1,1′‐(5‐bromo‐1,3‐phenylene)bis(3‐methyl‐1*H*‐imidazol‐3‐ium) dibromide) in 73% yield, which was subsequently reacted with Zr(NMe_2_)_4_ and transmetalated with FeBr_2_, using a method based on the one initially described for the synthesis of the parent complex [Fe^III^(ImP)_2_]PF_6_ by Hollis and Webster,^[^ ^22^ ^]^ and then adapted by Bauer and others,^[^ ^15^, ^16^, ^17^ ^]^ to give the complex [Fe^III^(ImPBr)_2_]PF_6_ in 41% yield (ImPBr = 2,6‐bis(3‐methylimidazol‐2‐ylidene‐1‐yl)(4‐bromophen‐1‐yl)).

In the case of the furanyl‐substituted ligand, 1,1′‐(5‐bromo‐1,3‐phenylene)bis(1*H*‐imidazole)^[^ ^21^ ^]^ was used in a Suzuki‐coupling using Pd_2_(dba)_3_ and the bulky PCy_3_ phosphine ligand as the catalytic system (dba = dibenzylideneacetone and PCy_3_ = tricyclohexylphosphine), giving the compound 1,1′‐(5‐(furan‐2‐yl)‐1,3‐phenylene)bis(1*H*‐imidazole) in 59% yield (Scheme 1). Subsequent methylation with methyl iodide gave the corresponding pre‐ligand HImPFur (HImPFur = 1,1′‐(5‐(furan‐2‐yl)‐1,3‐phenylene)bis(3‐methyl‐1*H*‐imidazol‐3‐ium) dibromide) in 71% yield, which underwent the transmetalation‐complexation reaction to give [Fe^III^(ImPFur)_2_]PF_6_ (ImPFur = 2,6‐bis(3‐methylimidazol‐2‐ylidene‐1‐yl)(4‐(furan‐2‐yl)phen‐1‐yl)) in 33% yield.

The ^1^H NMR spectrum of each of the complexes shows well‐resolved proton resonances, indicating low‐spin complexes. The spin state of both complexes was unambiguously assigned using Mößbauer spectroscopy, EPR, and SQUID magnetization studies (see Supporting Information). All results were consistent with an *S* = ½ d^5^ ground state.

**Figure 1:**
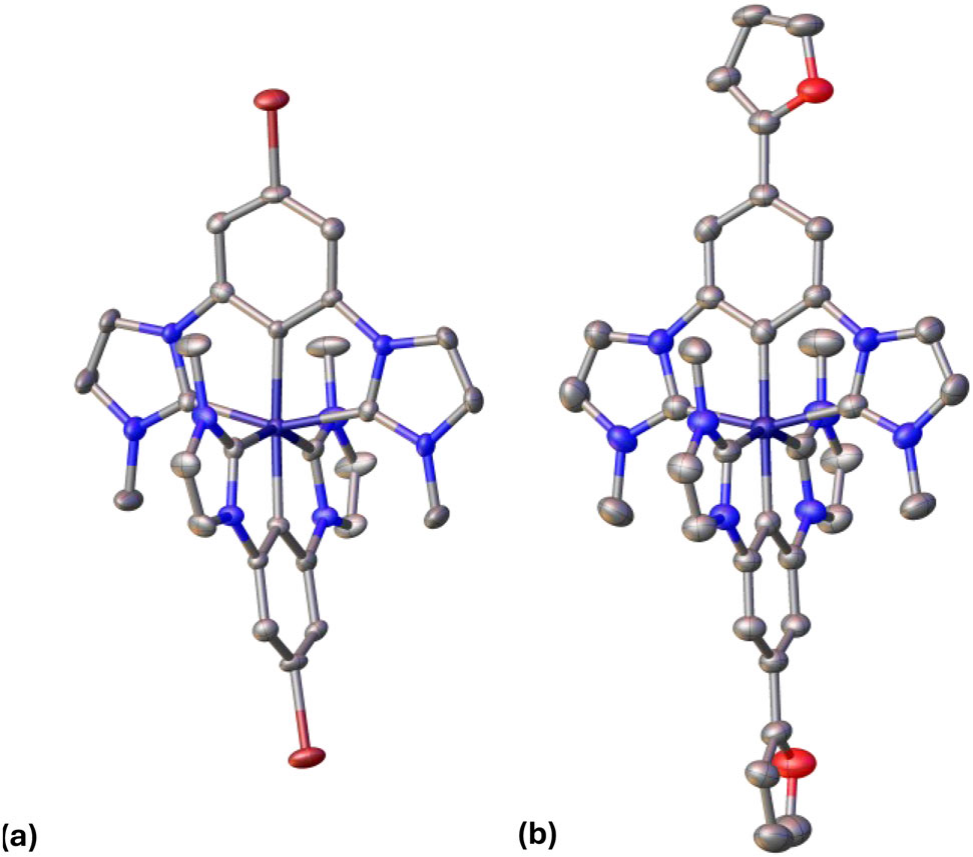
Molecular structure of (a) [Fe^III^(ImPBr)_2_]PF_6_ and (b) [Fe^III^(ImPFur)_2_]PF_6_, as determined by SC‐XRD. Counterions and solvent molecules are omitted for clarity. Displayed atoms are Fe – dark blue, C – gray, N – blue, Br – dark red, O‐ red.

Crystals of [Fe^III^(ImPBr)_2_]PF_6_ and [Fe^III^(ImPFur)_2_]PF_6_ suitable for single‐crystal XRD analysis were grown by slow diffusion of diethyl ether into an acetonitrile solution of the respective complexes resulting in the structures presented in Figure 1. The XRD analysis of [Fe^III^(ImPBr)_2_]PF_6_ resulted in bond lengths of 2.01 and 1.98 Å for the Fe–C(imidazole) bonds, and 1.94 Å fort he Fe–C(cyclometalated) bond. The bond angles for C(imidazole)–Fe–C(cyclometalated) (*cis*) were found to be 77.8° and 77.3°, for C(imidazole)–Fe–C(imidazole) (*trans*) 154.9° and 154.8°, and for C(cyclometalated)–Fe–C(cyclometalated), 172.8°. The XRD analysis of [Fe^III^(ImPFur)_2_]PF_6_ resulted in bond lengths of 1.98 and 1.99 Å for the Fe–C(imidazole) bonds and 1.94 Å for Fe–C(cyclometalated). The bond angles for C(imidazole)–Fe–C(cyclometalated) (*cis*) were 77.2° and 77.7°, for C(imidazole)–Fe–C(imidazole) (*trans*) 154.8° and 155.5°, and for C(cyclometalated)–Fe–C(cyclometalated), 178.8°. The dihedral angle between the planes constituting the furanyl‐ and the phenyl‐group is 4.6°, making electronic communication between the two π‐systems possible. As a comparison, the XRD analysis of the parent unsubstituted compound, [Fe^III^(ImP)_2_]PF_6_,^[^ ^16^ ^]^ resulted in bond lengths Fe–C(imidazole) = 1.93 Å and Fe–C(cyclometalated) = 1.94 Å. The bond angles for C(imidazole)–Fe–C(cyclometalated) (*cis*) were 76.7° each, for C(imidazole)–Fe–C(imidazole) (trans) 153.4° each, and for C(cyclometalated)–Fe–C(cyclometalated), 180°. In conclusion, adding the bromo‐ and furanyl‐ substituent to the ImP framework does only moderately change the bond distance and bond angles in the coordination sphere of this class of complexes.

Computationally‐optimized geometries of complexes [Fe^III^(ImPBr)_2_] ^+^ and [Fe^III^(ImPFur)_2_]^+^ (see Tables S8 and S9 and Figure S58) are consistent with the molecular structures obtained by XRD analysis (Figure 1) and the computational results confirm the electronic structure of the Fe^III^ complexes that is characterized by a ^2^A_1g_ ground state with an unpaired electron localized in the d_z_^2^ orbital of the Fe metal center. The potential energy curves (PECs) for the relevant electronic states are shown in Figure S60. The metal‐centered states,^[^ ^4^ ^] 6^MC, are calculated to be high in energy at the optimized doublet geometry, with the relaxed minima states displaced to longer Fe‐C bond lengths. This destabilization of the metal‐centered states is indicative of the strong σ‐donating capabilities of the ligands.

**Figure 2:**
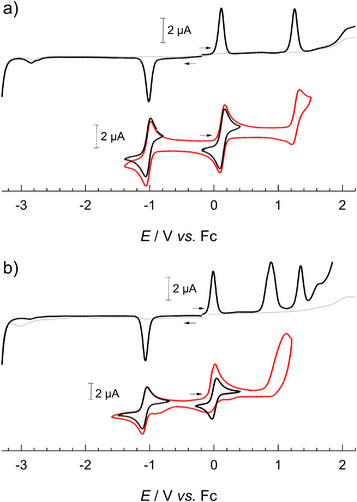
Voltammetry of (a) [Fe^III^(ImPBr)_2_]^+^ (0.82 mM) and (b) [Fe^III^(ImPFur)_2_]^+^ (0.85 mM) in deaerated acetonitrile with 0.1 M TBAPF_6_ as supporting electrolyte. Differential pulse voltammograms (top; step potential: 5 mV, modulation amplitude: 25 mV, modulation time: 50 ms, interval time: 100 ms) and cyclic voltammograms (bottom; scan rate: 0.05 Vs^−1^).

Voltammetric measurements revealed two reversible one‐electron waves of [Fe^III^(ImPBr)_2_]^+^ and [Fe^III^(ImPFur)_2_]^+^ (Figure 2) that can be attributed to the Fe^III/II^ and Fe^IV/III^ couples in analogy to the parent complex [Fe(ImP)_2_]^+^ and in agreement with the interpretation of the electronic absorption spectra (see below). Both complexes undergo further oxidation beyond their Fe(IV) state that is partially ([Fe^III^(ImPBr)_2_]^+^) or fully irreversible ([Fe^III^(ImPFur)_2_]^+^) and presumably ligand based. No ligand reductions were observed within the available potential window, consistent with the spectroscopic data that revealed correspondingly high‐energy MLCT transitions (see below). For complex [Fe^III^(ImPBr)_2_]^+^, both metal‐centered redox couples were shifted by about +0.1 V compared to the parent complex (Table 1) in line with the electron‐withdrawing nature of the Br substituents. Together with the relatively minor positive shift of the ligand‐based oxidation, these results are consistent with the moderate red‐shift of the lowest energy LMCT absorption band (see below). Also for complex [Fe^III^(ImPFur)_2_]^+^, the Fe(III/II) couple shows a positive shift of the same magnitude found for the Br‐analogue. With respect to this metal‐based reduction of the complex, the combined inductive and mesomeric effects of the furanyl substituents can hence be considered as net electron withdrawing. While the Fe(IV/III) couple is actually located at slightly lower potential, the average potential of the metal centered couples is still shifting in positive direction in line with an electron withdrawing effect of the furanyl substituents, and the reduced separation between these couples might indicate increased charge delocalization over the ligands in the higher oxidation states. In contrast to the Br‐analogue, the potential for the first ligand‐based oxidation is decreased in case of [Fe^III^(ImPFur)_2_]^+^ which is in line with the larger π‐system of the ImPFur ligand and the furanyl substituents would in this respect have electron donating character. In combination with the higher potential of the Fe(III/II) couple, it is in particular the easier ligand oxidation that accounts for the more substantially lowered LMCT excitation energy of [Fe^III^(ImPFur)_2_]^+^ (see below).

**Table 1:** Ground and excited state redox properties of the studied [Fe^III^L_2_]^+^ complexes and absorption properties of [Fe^III^L_2_]^+^, [Fe^II^L_2_], and [Fe^IV^L_2_]^2+^.

	*E*_1/2_ / V vs Fc			*λ*_max_ / nm (*ε* / 10^4^ M^−1^cm^−1^)		
Complex^[^ ^a^ ^]^	Fe^III/II^ (Fe^*III/II^)^[^ ^c^ ^]^	Fe^IV/III^ (Fe^IV/*III^)^[^ ^c^ ^]^	L^.+^/L^[^ ^b^ ^]^	[Fe^II^L_2_]	[Fe^III^L_2_]^+^	[Fe^IV^L_2_]^2+^
[Fe(ImP)_2_]^0/+/2+^ ^[d]^	−1.15 (0.75)	0.04 (−1.96)	1.22	329, 404 (0.62, 1.17)	352, 581, 618 (0.41, 0.04, 0.04)	499, 776 (0.19, 0.16)
[Fe(ImPBr)_2_]^0/+/2+^	−1.02 (0.83)	0.13 (−1.72)	1.26	336, 412 (0.49, 0.92)	344, 595, 639 (0.48, 0.05, 0.05)	504, 808 (0.18, 0.16)
[Fe(ImPFur)_2_]^0/+/2+^	−1.02 (0.61)	0.01 (−1.62)	∼0.9^[^ ^e^ ^]^	355, 468 (1.03, 2.29)	380, 668, 726 (1.89, 0.18, 0.20)	412, 558, 979 (1.20, 0.38, 1.40)

^[a]^ As PF_6_^−^ salts in acetonitrile with 0.1 M TBAPF_6_.

^[b]^ Potential of first ligand based oxidation.

^[c]^ Excited state potentials of *[Fe^III^L_2_]^+^ estimated as E*_1/2_ = *E*_1/2_ ± *E*_0‐0_ with energies *E*_0‐0_ of the vibrationless ^2^LMCT states given in Table 2, neglecting coulombic work terms.

^[d]^ Ref. [16]

^[e]^ Irreversible oxidation, DPV peak potential.

**Figure 3:**
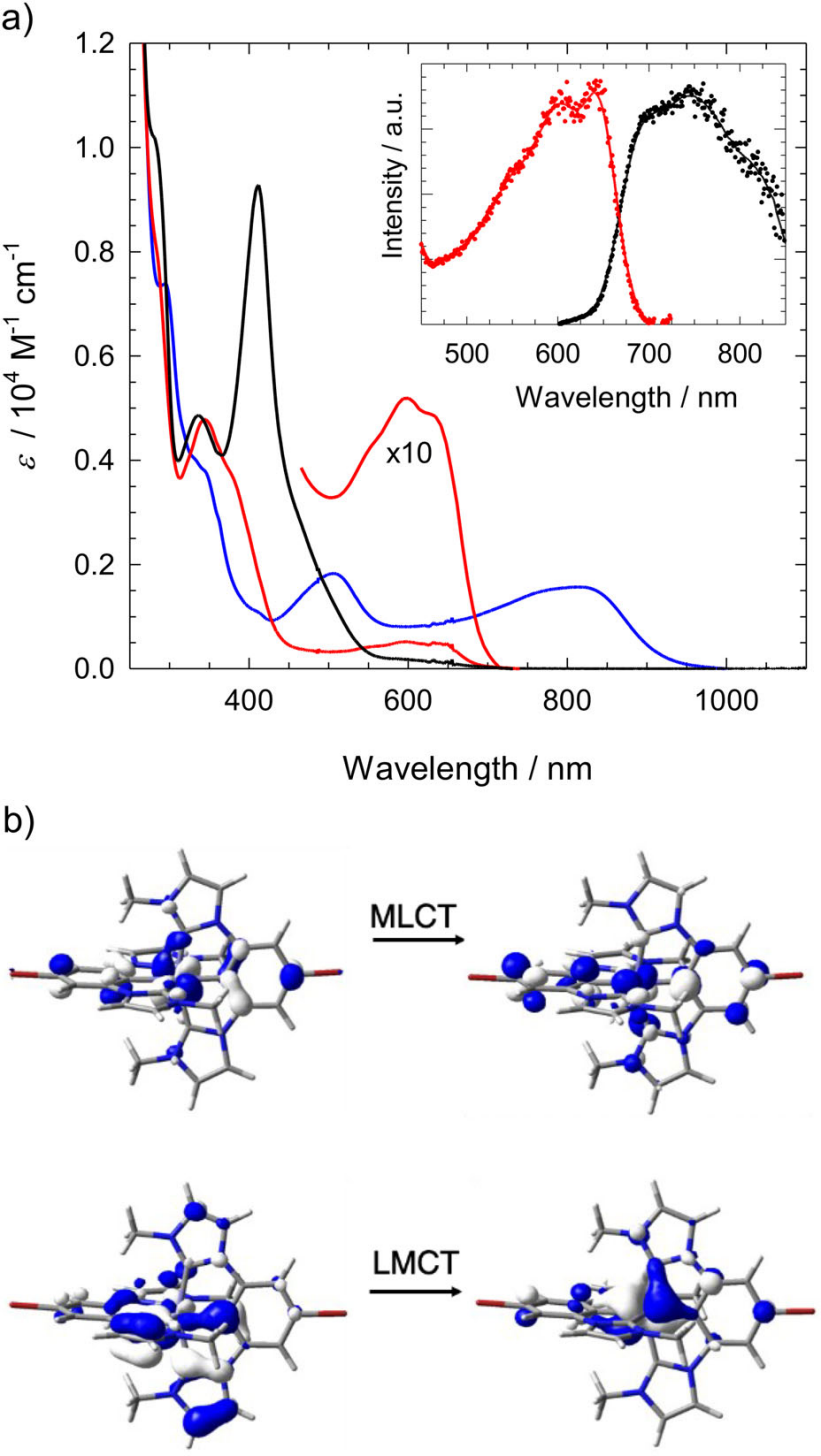
(a) Electronic absorption spectra (–) of [Fe^III^(ImPBr)_2_]^+^ in acetonitrile and spectra of [Fe^IV^(ImPBr)_2_]^2+^ (–) and [Fe^II^(ImPBr)_2_] (–) obtained by spectroelectrochemistry. Inset: Emission (black, *λ*_ex_ = 585 nm) and excitation spectra (red, *λ*_em_ = 750 nm) of [Fe^III^(ImPBr)_2_]^+^ in acetonitrile at r.t. together with its scaled absorption spectrum. (b) Computed orbitals of [Fe^III^(ImPBr)_2_]^+^ illustrating higher energy MLCT and lower energy LMCT transitions attributed to the near‐UV and visible absorption bands, respectively.

**Figure 4:**
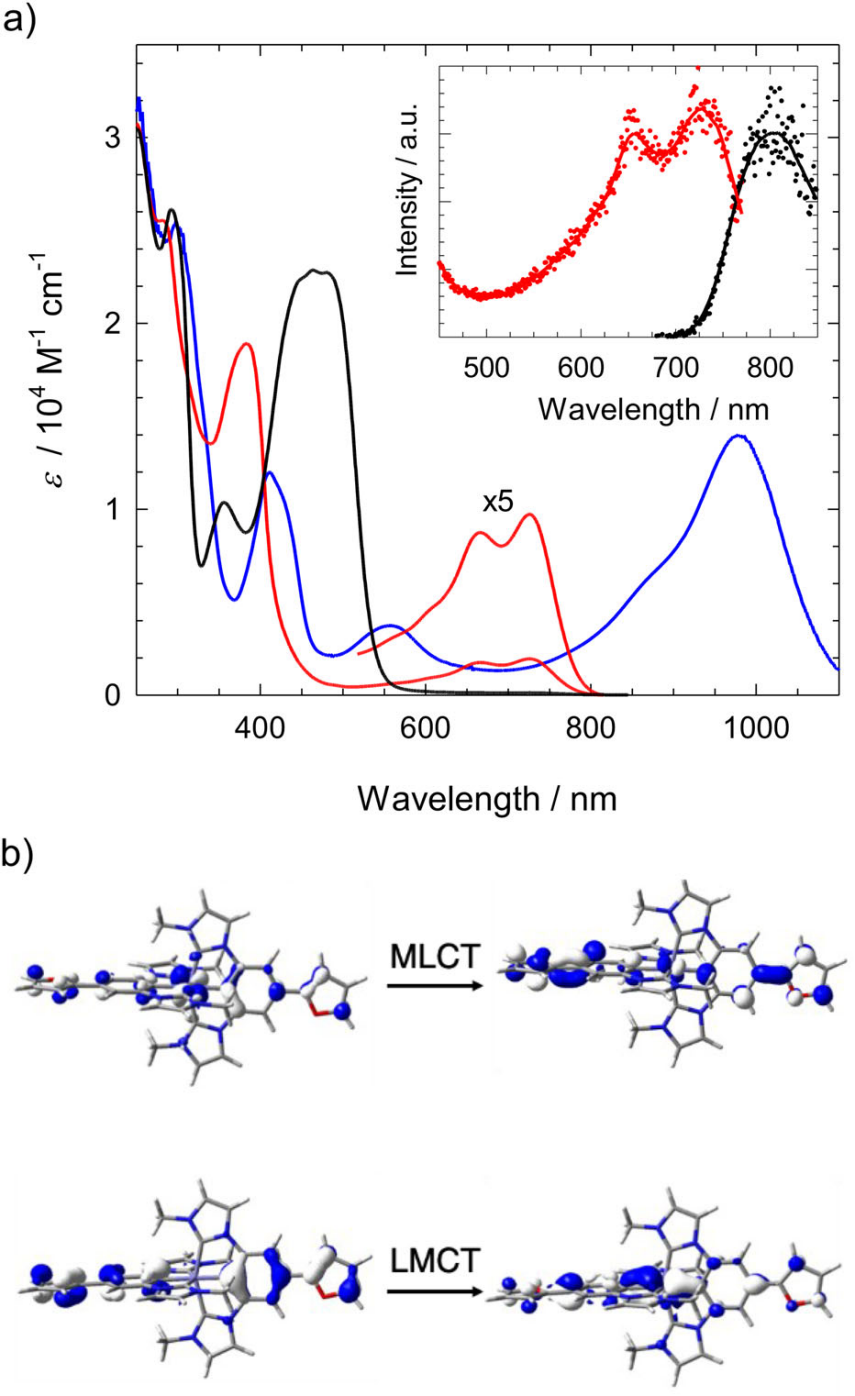
(a) Electronic absorption spectra (–) of [Fe^III^(ImPFur)_2_]^+^ in acetonitrile and spectra of [Fe^IV^(ImPFur)_2_]^2+^ (–) and [Fe^II^(ImPFur)_2_] (–) obtained by spectroelectrochemistry. Inset: Emission (black, *λ*_ex_ = 660 nm) and excitation spectra (red, *λ*_em_ = 800 nm) of [Fe^III^(ImPFur)_2_]^+^ in acetonitrile at r.t. together with its scaled absorption spectrum. (b) Computed orbitals of [Fe^III^(ImPFur)_2_]^+^ illustrating higher energy MLCT and lower energy LMCT transitions attributed to the near‐UV and visible absorption bands, respectively.

The electronic absorption spectra of [Fe^III^(ImPBr)_2_]^+^ and [Fe^III^(ImPFur)_2_]^+^ are shown in Figures 3a and 4a together with the spectra of their Fe^IV^ and Fe^II^ analogues obtained by electrochemical one‐electron oxidation and reduction, respectively. Both Fe^III^ complexes (red spectra) exhibit a narrow higher‐energy band peaking around 400 nm, and a broader less intense band extending to about 650 ([Fe^III^(ImPBr)_2_]^+^) and 750 nm ([Fe^III^(ImPFur)_2_] ^+^) similar to what is observed with the parent complex.^[^ ^15^, ^16^ ^]^ Assignment of the high‐ and low‐energy absorption bands to d^5^‐π*_L_ MLCT and π_L_‐d^5^ LMCT transitions, respectively, is corroborated by the electrochemical data that indicates relatively higher‐energy MLCT transitions and accounts for the increasing red shift of the LMCT band by the Br‐ and furanyl substituents. The above assignment of the absorption bands is further supported by DFT calculations (see ESI, Figure S57 and the molecular orbitals involved in the lowest energy MLCT and LMCT transitions depicted in Figures 3b and 4b). In good agreement with the above‐mentioned experimental data, the computational spectra (Figure S59), indicate higher oscillator strengths of [Fe^III^(ImPFur)_2_] ^+^ and a pronounced red shift of its LMCT band and computed MO energy levels (Figure S60) confirm that the latter effect can be attributed primarily to the destabilization of the ligand‐based HOMO as a result of the enlarged ligand π‐system.^16^

In their oxidized state ([Fe^IV^L_2_]^2+^) the Br‐ and furanyl‐substituted complexes are characterized by absorption bands extending well into the near infrared (NIR) region (Figures 3a and 4a, Table 1) that can be attributed to low‐energy π_L_‐d^4^ LMCT transitions while the 400–500 nm absorption of the reduced complexes [Fe^II^L_2_] is assigned to d^6^‐π*_L_ MLCT transitions. These assignments and the red shift of both types of transitions in relation to the parent complex^[^ ^15^, ^16^ ^]^ are consistent with the above interpretation of the electrochemical data. Specifically, the pronounced red shift and increased oscillator strength of the LMCT band of the furanyl‐substituted Fe^IV^ complex can be ascribed to the larger ligand π‐system and the correspondingly lower potential for ligand oxidation as discussed above.

[Fe^III^(ImPBr)_2_]PF_6_ and [Fe^III^(ImPFur)_2_]PF_6_ exhibit weak photoluminescence in the deep red to NIR region (Figures 3a and 4a, insets). With excitation spectra closely tracking the absorption spectra, the emission can be attributed to the ^2^LMCT state of the complexes. Compared to the parent complex, the emission spectra display similar redshifts as the lowest‐energy absorption bands and the energies of the vibrationless excited states estimated from the intersection of absorption and emission spectra are lowered by 0.05 and 0.27 eV by the Br‐ and furanyl‐substituents, respectively (Table 2). The emission quantum yield of [Fe^III^(ImPBr)_2_]PF_6_ is somewhat higher than that for the parent complex while a signifficantly lower yield is found in case of [Fe^III^(ImPFur)_2_]PF_6_ (Table 2) that is attributed to a faster non‐radiative decay (see below). Next to the genuine NIR luminescence from the ^2^LMCT excited states, a weak blue emission is observed upon UV excitation (Figure S57) which, based on the excitation spectrum, can be attributed to minor quantities of an impurity similar to the case for the parent complex.^[^ ^15^, ^16^ ^]^

**Table 2:** Excited state properties of the studied [Fe^III^L_2_]^+^ complexes.

Complex^[^ ^a^ ^]^	*λ*_em, max_ / nm	*E*_0‐0_ / eV	*Φ*_em_ / %	*τ*_1_ / ps (^2^MLCT’)	*τ*_2_ / ps (^2^MLCT)	*τ*_3_ / ps (^2^LMCT)	*k*_r_^[^ ^b^ ^]^/ 10^6^ s^−1^	*k*_nr_^[^ ^c^ ^]^/10^9^ s^−1^
[Fe^III^(ImP)_2_]^+^^[^ ^d^ ^]^	731	1.90	0.05	0.50	9	240	2.1	4.2
[Fe^III^(ImPBr)_2_]^+^	745	1.85	0.10	0.14	7	255	3.9	3.9
[Fe^III^(ImPFur)_2_]^+^	795	1.63	∼0.01	0.25	9	59	1.7	16.9

^[a]^ As PF_6_^−^ salts in acetonitrile at room temperature.

^[b]^ $k_{r}=\Phi_{\mathrm{em}}/\tau_{\mathrm{LMCT}}$.

^[c]^ $k_{\mathrm{nr}}=\left(1-\Phi_{\mathrm{em}}\right)/\tau_{\mathrm{LMCT}}$.

^[d]^ Ref. [16]

**Figure 5:**
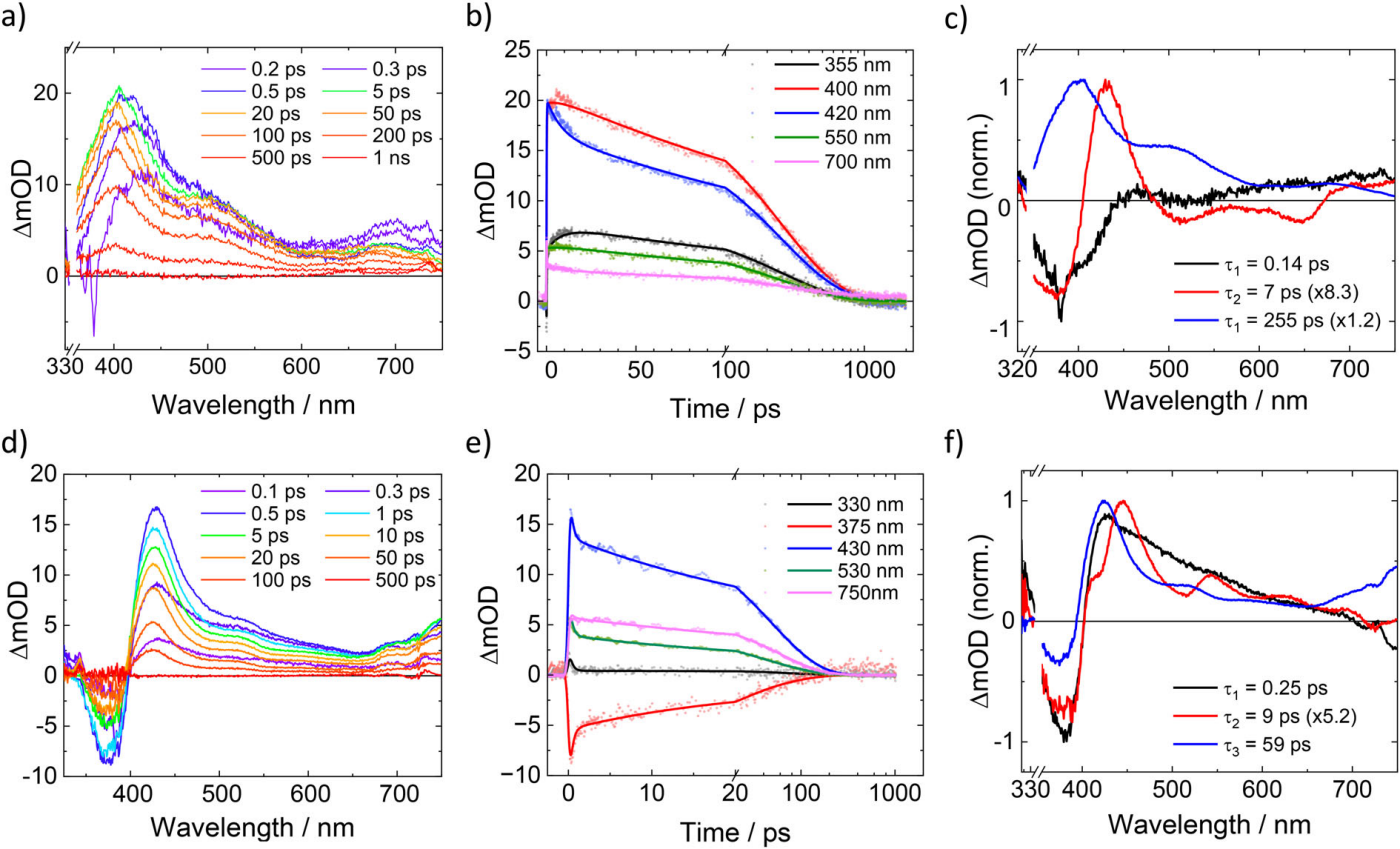
Femtosecond transient absorption spectroscopy of [Fe^III^(ImPBr)_2_]^+^ (a–c) and [Fe^III^(ImPFur)_2_]^+^ (d–f) excited at 350 nm. Left: Transient absorption spectra at indicated delay times. Middle: Kinetic traces at selected wavelengths. Right: Normalized decay‐associated spectra obtained from global analysis.

The excited state dynamics of [Fe^III^(ImPBr)_2_]^+^ and [Fe^III^(ImPFur)_2_]^+^ were investigated by femtosecond‐transient absorption spectroscopy (fs‐TAS) and selected TA spectra and kinetics obtained upon excitation into their MLCT band (*λ*_ex_ = 350 nm) are displayed in Figure 5. For complex [Fe^III^(ImPBr)_2_]^+^, excitation resulted in positive transient absorption throughout the visible spectrum, with some negative features below 400 nm during the first couple of hundred femtoseconds (Figure 5a). At later times this ground state bleach vanishes due to the rising transient absorption in the blue and near UV range, similar to what was observed with the parent complex.^[^ ^15^, ^16^ ^]^ Also in case of [Fe^III^(ImPFur)_2_]^+^, the broad excited state absorption features (Figure 5d) strongly resemble the data for the parent complex but are somewhat red‐shifted and overlapping less with the bleach of the intense ground state absorption below 400 nm. Most notably, the decay of all transient absorption and the simultaneous recovery of the ground state absorption for [Fe^III^(ImPFur)_2_] ^+^ is essentially complete in about 100 ps, i.e., significantly faster than with [Fe^III^(ImPBr)_2_] ^+^. Global analysis revealed that the excited state deactivation of both complexes involves three spectrally and kinetically distinguishable components. The corresponding time constants and the evolution‐associated spectra for a sequential fit model are compiled in Table 2 and Figures 5c,f. The sub‐ps process is assigned to the relaxation of the initially populated ^2^MLCT state (^2^MLCT’) and the relaxed ^2^MLCT states of both complexes subsequently decay within a few ps to the lower lying and longer‐lived ^2^LMCT states as previously observed with the parent complex^[^ ^15^, ^16^ ^]^ and related complexes.^[^ ^17^ ^]^ Furthermore, the assignment of the longest‐lived component in the fs‐TA data for [Fe^III^(ImPBr)_2_]^+^ and [Fe^III^(ImPFur)_2_] ^+^ to their ^2^LMCT state is clearly evidenced by complementary fs‐TAS data obtained upon excitation into the lower‐energy LMCT band that resulted in spectra and lifetimes basically identical with the transient ultimately populated upon MLCT excitation (Figures S54–S56). Overall, the excited state dynamics of the substituted complexes are hence analogous to the parent complex but differ most significantly in terms of the ^2^LMCT lifetimes. While the ^2^LMCT state of [Fe^III^(ImPBr)_2_]^+^ is somewhat longer lived relative to the parent complex, its lifetime is substantially shorter in case of [Fe^III^(ImPFur)_2_]^+^. With the radiative decay contributing negligibly to the ^2^LMCT deactivation, the variation in lifetime arises primarily from differences in the non‐radiative rate constants (Table 2). The slightly slower nonradiative decay of [Fe^III^(ImPBr)_2_]^+^ can be tentatively attributed to the somewhat lower LMCT energy, which would disfavor the deactivation via metal‐centered (MC) states, specifically the ^4^MC state. On the other hand, the particularly fast nonradiative decay for complex [Fe^III^(ImPFur)_2_]^+^ suggests that its even lower excited state energy leads to faster deactivation straight to the GS, as governed by the energy gap law.^[^ ^23^, ^24^, ^25^ ^]^ This notion is in line with the even faster deactivation (6.5 ps) of the exceedingly stabilized ^2^LMCT state (1.41 eV) of the N(*p*‐anisyl)_2_ analogue previously reported by Wellauer et al.^[^ ^17^ ^]^)

**Scheme 2:**
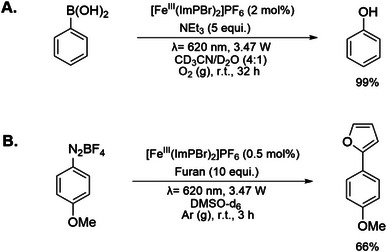
Photoredox catalysis reactions driven by red‐light irradiation of [Fe^III^(ImPBr)_2_]^+^. **A** Photocatalytic aerobic oxidative hydroxylation of phenylboronic acid in an oxygen‐saturated deuterated acetonitrile/water mixture. **B** Photocatalytic reductive C─H arylation of 4‐methoxyphenyl diazonium tetrafluoroborate with furan in nitrogen purged DMSO‐*d*_6_. The reactions were performed in an NMR tube irradiated with a red LED (620 nm, output power 3.47 W). The yields were determined based on ^1^H NMR spectroscopy using trimethyl(phenyl)silane and 1,3,5‐trimethoxybenzene, respectively, as the internal standards.

In terms of the potential use of this type of complexes in applications as photocatalysts, it was already demonstrated with the phenyl‐substituted complex, [Fe^III^(ImPP)_2_]PF_6_ (ImPP = bis(2,6‐bis(3‐methylimidazol‐2‐ylidene−1‐yl)biphenylene) studied by Wellauer et al., that it can be employed in photoredox catalysis and triplet‐triplet upconversion.^[^ ^17^ ^]^ The [Fe^III^(ImPP)_2_]^+^ complex benefits from an excited state with good oxidizing (0.75 V versus Fc^0/+^) and reducing properties (−1.96 V versus Fc^0/+^)^[^ ^17^, ^26^ ^]^ and its [Fe^III^(ImPBr)_2_]^+^ analogue offers comparable characteristics with a somewhat more oxidizing yet slightly less reducing ^2^LMCT state (0.83, −1.72 V), that should enable its application in similar photoredox catalysis reactions. The oxidizing properties of [Fe^III^(ImPBr)_2_]^+^ were demonstrated in the photocatalytic hydroxylation of phenylboronic acid (Scheme 2A). Under an oxygen atmosphere that ensures regeneration of the reduced [Fe^II^(ImPBr)_2_] photosensitizer, the reaction reached a 99% ^1^H NMR yield. Furthermore, the ^2^LMCT state of [Fe^III^(ImPBr)_2_]PF_6_ is still sufficiently reducing to enable oxidative quenching by 4‐methoxyphenyl diazonium tetrafluoroborate, which paved the way for a C‐H arylation with furan as the coupling partner in 66% yield (Scheme 2B). For both reactions, control experiments using previously published parent complex [Fe^III^(ImP)_2_]PF_6_ and phenyl substituted [Fe^III^(ImPP)_2_]PF_6_^[^ ^17^ ^]^ under the exact same conditions resulted in identical conversions and yields (see Tables S10 and S11).

## 3 Conclusions

We show that Br‐ and furanyl‐substituents have opposing effects on the excited state lifetime of cyclometaled Fe(III) carbene complexes based on the established [Fe(ImP)_2_]^+^ motif. The purely inductive EW effect of bromo‐substituents attached to the cyclometalating part of the ImP ligands affects primarily the potential of the Fe(III/II) couple, which lowers the energy of the ^2^LMCT state to a value of 1.85 eV. This moderate stabilization (0.05 eV) results in a somewhat extended lifetime (255 ps) compared to the parent complex (240 ps), presumably due to an increased barrier for intersystem crossing from the ^2^LMCT state to the ^4^MC state. The resulting combination of excited state lifetime and energy enables applications of the Br‐substituted complex in photoredox catalysis. In contrast, the furanyl groups drastically decrease the ^2^LMCT state lifetime to 59 ps despite its substantially lowered energy of 1.63 eV. The pronounced stabilization of the ^2^LMCT by the furanyl substituents arises primarily from the facile oxidation of the ligand with its extended π‐system, and the rapid ^2^LMCT deactivation is presumably governed by internal conversion directly to the ground state that is accelerated by the decreased energy gap. In general terms, these results illustrate the critical limitations of lowered LMCT energies as an approach to improved excited state lifetimes. Similar limitations need to be considered when tuning ground and excited state redox properties by means of electronic substituent effects that inevitably alter the energetics of the charge transfer states. Specifically, our findings show that for complexes based on the [Fe(ImP)_2_]^+^ motif the critical threshold for the ^2^LMCT energy required to avoid rapid internal conversion is located well above 1.65 eV.

## Supporting Information

Experimental details and additional experimental (NMR, HRMS, XRD, EPR, Mössbauer spectroscopy, magnetic susceptibility and magnetization, fs‐TAS, photoredox catalysis) and computational data. The authors have cited additional references within the Supporting Information.^[^ ^13^, ^27^, ^28^, ^29^, ^30^, ^31^, ^32^, ^33^, ^34^, ^35^, ^36^, ^37^, ^38^, ^39^, ^40^, ^41^, ^42^, ^43^, ^44^, ^45^, ^46^, ^47^ ^]^

## Conflict of Interest

The authors declare no conflicts of interest.

## Supporting information



Supporting Information

Supporting Information

## Data Availability

Deposition numbers 2455513 (for [HImPBr](PF_6_)_2_), 2455514 (for [Fe(ImPFur)_2_]PF_6_), 2455515 (for [HImPFur](PF_6_)_2_), and 2455516 (for [Fe(ImPBr)_2_]PF_6_) contain the supplementary crystallographic data for this paper. These data are provided free of charge by the joint Cambridge Crystallographic Data Centre and Fachinformationszentrum Karlsruhe Access Structures service.
