# Supplementary material for: Tuning the 2LMCT Deactivation of Cyclometalated Iron Carbene Complexes with Electronic Substituent Effects
Source: Chemistry. 2025 Jul 25;31(47):e01985. doi: 10.1002/chem.202501985 (PMC12376266; doi:10.1002/chem.202501985)
Supplement: Supplementary file 1 — Supporting Information [file CHEM-31-e01985-s001.pdf]

Supporting Information

Tuning the ^{2}LMCT Deactivation of Cyclometalated Iron Carbene Complexes with Electronic Substituent Effects

Abhishek Mishra,[a] Kumkum Sharma,[a] Catherine Ellen Johnson,[b] Emmanuel Adu Fosu,[c] Jesper Schwarz,[a] Om Prakash,[a] Arvind Kumar Gupta,[a] Ping Huang,[b] Fredrik Lindgren,[d] Lennart Häggström,[d] Jesper Bendix,[e] Elena Jakubikova,*[c] Reiner Lomoth,*[b] and Kenneth Wärnmark*[a]

[a]*Centre for Analysis and Synthesis, Department of Chemistry, Lund University, Box 124, SE-22100 Lund, Sweden*

[b]*Department of Chemistry – Ångström Laboratory, Uppsala University, Box 523, SE-75120 Uppsala, Sweden*

[c]*Department of Chemistry, North Carolina State University, Raleigh, North Carolina 27695, United States*

[d]*Department of Physics – Ångström Laboratory, Uppsala University, Box 538, SE-75121 Uppsala, Sweden*

[e]*Department of Chemistry, University of Copenhagen, Universitetsparken 5, DK-2100 Copenhagen, Denmark*

*Corresponding Authors

E-mail: kenneth.warnmark@chem.lu.se

Contents

S1. Experimental Section **3**

Compound Characterizations *3*

Photoredox Catalysis Reactions *3*

Cyclic Voltammetry and UV-Vis Spectroelectrochemistry Measurements *3*

Steady State Spectroscopy Measurements *4*

Femtosecond Transient Absorption Measurements *4*

Global Analysis *4*

DFT Methodology *4*

S2. Synthesis and Characterization **5**

S2.1 Synthesis of [Bis(2,6-bis(3-methylimidazol-2-ylidene-1-yl)4-bromophenylene) iron (III)] hexafluorophosphate, [Fe(ImPBr)$_2$]PF$_6$ *5*

S2.2 Synthesis of [Bis(2,6-bis(3-methylimidazol-2-ylidene-1-yl)4-(furan-2-yl)phen-4 yl) iron(III)] hexafluorophosphate, [Fe(ImPFur)$_2$]PF$_6$ *7*

S3. NMR Spectra **10**

S4. HRMS **29**

S5. Single Crystal X-ray Structure Determination **32**

S6. Electron paramagnetic resonance measurements **36**

S7. Magnetic susceptibility and magnetization measurements **36**

S8. Mössbauer Measurements **38**

S9. Fs-TAS and Global Analysis **40**

S10. Quantum Chemical Calculations **41**

S11. Photoredox Catalysis Reactions **43**

S11. References **47**

S1. Experimental Section

Compound Characterizations

NMR spectra were recorded on a Bruker Avance III 500 MHz or Bruker Avance Neo 600 MHz NMR spectrometer. Chemical shifts (δ) are referenced with respect to the residual NMR solvent; CD_3OD (3.31 ppm for ^{1}H NMR; 49.00 ppm for ^{13}C NMR spectra spectra), CD_3CN (1.94 ppm for ^{1}H NMR spectra; 1.32 and 118.36 ppm for ^{13}C NMR spectra) and DMSO-d_6 (2.50 ppm for ^{1}H NMR; 39.52 ppm for ^{13}C NMR spectra spectra). All samples were recorded in solution phase at 298 K. Coupling constants were indicated by *J* in Hz. Electrospray ionization–high resolution mass spectrometry (ESI–HRMS) data were recorded on a Waters Xevo-G2 QTOF spectrometer. Melting points of the compounds were measured on a Stuart Scientific Melting Point Apparatus-SMP3. Elemental analyses were performed by A. Kolbe, Mikroanalytisches Laboratorium, Germany.

Acetonitrile (≥99.95%, HiPerSolv CHROMANORM® Chemicals BDH, VWR) was air-removed by purging nitrogen gas prior to dissolve the Fe-containing complexes to a final concentration of 1.0 mM. A volume of 150 μL of each solution was transferred to an EPR tube and stored in 77 K dewar before EPR exam. EPR measurement was performed on a Bruker EMX-micro spectrometer equipped with an EMX-Premium bridge and an ER4119HS resonator in connection with an Oxford Instruments continuous flow cryostat. Measuring temperatures were achieved using liquid helium flow through an ITC 503 temperature controller (Oxford Instruments). EPR recording settings: 10 K; microwave frequency 9.31 GHz, microwave power 0.2 mW, modulation frequency 100 MHz, modulation amplitude 10 G.

Mössbauer measurements were carried out in an Oxford Instrument flow cryostat at 85 K and at 295 K using a ^{57}CoRh source held at room temperature. The studied powder materials were pressed and formed as disc absorbers with a concentration of ~ 35 mg/cm^2 (Complex **1**) and ~ 80 mg/cm^2 (Complex **2**). Calibration spectra were recorded from a natural a-Fe metal foil held at 295 K. The resulting spectra were analyzed using a least square Mössbauer fitting program.

Photoredox Catalysis Reactions

All reactions were carried out in nitrogen atmosphere using Schlenk line. Reaction preparation for photoredox reaction was carried out inside the glove box under N_2 environment. THF was dried over Na/benzophenone and was subsequently distilled under nitrogen prior to use. All the solvents used for reactions were purchased from Thermo Scientific, Honeywell in reagent grade and used as received. Extra dry acetone was purchased from Thermo Scientific with 99.8% purity. All the deuterated solvents were purchased in high purity from Sigma Aldrich and Acros Organics. Commercially available reagents and starting materials were purchased from Sigma Aldrich, Acros, Fischer Scientific, and used as received without any further treatment.

Cyclic Voltammetry and UV-Vis Spectroelectrochemistry Measurements

All electrochemical experiments were performed in acetonitrile (spectroscopic grade Uvasol®, ≥99.9%, Merck; dried over 3Å molecular sieves activated at 300 °C for 15 hours) with 0.1 M tetrabutylammonium hexafluorophosphate (electrochemical grade, Sigma Aldrich; dried at 80 °C under vacuum) and purged with solvent-saturated argon.

Cyclic voltammetry (CV) measurements were carried out in a three-electrode electrochemical cell, using an AUTOLAB potentiostat (PGSTAT302) controlled with GPES software (Version 4.9). The working electrode was a freshly polished (with Buehler alumina paste) glassy carbon electrode (CH Instruments, 1 mm diameter); the reference electrode was a non-aqueous Ag/$AgNO_3$ (CH Instruments; 10 mM of $AgNO_3$ dissolved in dried acetonitrile; 0.091 V vs. ferrocene) and a Pt wire in a separate compartment was used as counter electrode. UV-Vis spectroelectrochemistry measurements were performed in a diode array spectrophotometer (Agilent 8453) with an optically transparent thin-layer cell (1 mm optical path length) equipped with a platinum mesh working electrode and the same reference and counter electrodes used

for voltammetry. Time-resolved spectra were recorded during controlled potential electrolysis using an AUTOLAB potentiostat (PGSTAT302).

Steady State Spectroscopy Measurements

Samples were prepared in acetonitrile (spectroscopic grade Uvasol®, ≥99.9%, Merck) in either a 1 mm or a 10 mm quartz cuvette. UV-Vis absorption spectra were recorded on Varian Cary 50 or Cary 5000 spectrophotometers. Steady-state emission and excitation measurements were performed on a Fluorolog-3 (Horiba) fluorimeter with 5 nm spectral resolution. Samples were prepared in 10 mm cuvettes and measurements were performed with right-angle geometry. Emission and excitation spectra were all background subtracted and corrected for detector response.

Femtosecond Transient Absorption Measurements

Femtosecond transient absorption spectroscopy (fs-TAS) measurements were performed probing in the UV-Vis region on a Newport TAS system with a Coherent Libra Ti:sapphire amplifier (800 nm, 1.5 mJ, 3 kHz repetition rate, FWHM 45 fs). Different excitation wavelengths were generated by optical parametric amplifiers (TOPAS-Prime and NIRUVVIS, Light Conversion) and then focused and centered on the 1 mm cuvette with corresponding pump powers. The white light supercontinuum probe light was generated using a CaF_2 crystal (Crystran) and was detected by a silicon diode array (Newport custom made). A mechanical chopper blocked every other pump pulse, and the transient absorption at each time point was calculated for an average of 300 ms chopped/un-chopped pulse pairs. To record the transient absorption spectra at different time points, an optical delay line was used to scan the delay of the probe beam relative to the pump beam. Prior to analysis, the data was corrected for the spectral chirp using Surface Xplorer v4, where single wavelength fits were also performed.

Global Analysis

All fs-TAS data were further analyzed with Global Analysis using the software Glotaran (Version 1.5.1),[1 1] which is a Java-based graphical user interface to the R package TIMP. All data were fitted with the necessary number of components and the decay-associated spectra (DAS) were exported from the software and analyzed in Origin.

DFT Methodology

All calculations were performed with the Gaussian 16 software package Revision A.03[2] utilizing the B3LYP[3–5] hybrid functional with Grimme's D2 dispersion corrections.[6] Solvation effects were accounted for using the PCM implicit solvation model[7] with acetonitrile as the solvent. Geometry optimization and frequency calculations were done using the split-valence triple-zeta 6-311G* basis sets for C, N, O, and H atoms, and the SDD pseudopotential with the accompanying basis sets for Fe.[8,9] An ultrafine grid was used for all calculations. Full optimization of all minima on the PES was done without symmetry constraints. The nature of all optimized structures was verified by frequency calculations, that is, all optimized structures corresponded to minima with no imaginary frequencies. Wavefunction stability tests were performed on all complexes, and only complexes with stable wavefunctions are reported. The electronic states of all true minima were further characterized using the natural orbital (NO) analysis.[10] The frontier molecular orbitals have been analyzed using fragment molecular orbital analysis as implemented in the AOMix software.[11] UV-Vis spectra were calculated utilizing the time-dependent density functional theory (TD-DFT)[12,13] with the same model chemistry as the ground state optimizations. The stick spectra were broadened using Lorentzian functions with a half-width-at-half-maximum (HWHM) of 0.14 eV. The hole and particle pairs for each transition in the calculated absorption spectra were characterized using natural transition orbitals (NTO).[14] One-dimensional potential energy curves versus the average Fe-C bond distances were constructed for the various electronic states of the studied Fe(III) complexes to understand the excited-state dynamics. Single point energy calculations utilizing the ground state DFT approach were carried out at the fully optimized structures, to obtain their corresponding ^{2}GS, ^{4,6}MC states for Fe(III) states.

S2. Synthesis and Characterization

S2.1 Synthesis of [Bis(2,6-bis(3-methylimidazol-2-ylidene-1-yl)4-bromophenylene) iron (III)] hexafluorophosphate, [Fe(ImPBr)$_2$]PF$_6$

Synthesis of 1,1'-(5-bromo-1,3-phenylene)bis(1H-imidazole)

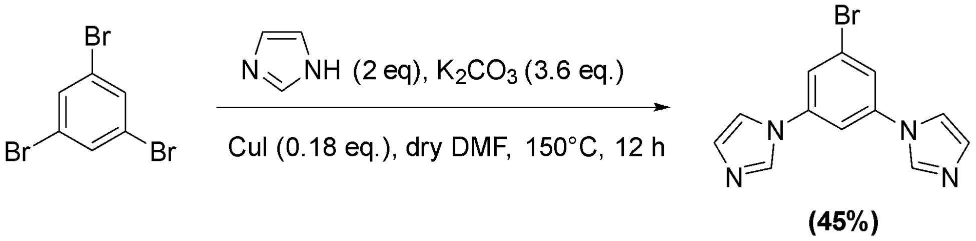

A pressure tube was charged with 1,3,5-tribromobenzene (5.25 g, 16.7 mmol), imidazole (2.25 g, 33 mmol), potassium carbonate (8.29 g, 60 mmol), and copper iodide (0.57 g, 3 mmol) in 40 mL dry DMF under N_2 flow and the mixture was continuously stirred while heating to 150 °C for 12 h. The reaction mixture was kept cooling at room temperature. The brown suspension was filtered on a celite pad using suction and the solid was washed with DCM. The organic phase was further washed with water and brine and dried over Na_2SO_4. The organic phase was evaporated under vacuum resulting into yellow solid. The residue was passed through silica column (DCM/MeOH, 30/1) and the compound was collected as off-white solid. Yield: 2.15 g (45 %). R_f = 0.74 (MeOH/DCM: 1/9), M.P. 190-193°C.

^{1}H NMR (500 MHz, CD$_3$OD) δ 8.32 (s, 2H, H_{Im-f}), 7.88 (t, *J* = 1.9 Hz, 1H, H_{Ph-g}), 7.85 (d, *J* = 2.0 Hz, 2H, H_{Ph-b}), 7.73 (t, *J* = 1.4 Hz, 2H, H_{Im-d}), 7.17 (s, 2H, H_{Im-e}).

^{13}C{^{1}H} NMR (126 MHz, CD$_3$OD) δ 140.6 (H_{Ph-c}), 137.2 (H_{Im-f}), 130.7 (H_{Im-e}), 125.4 (H_{Ph-a}), 123.5 (H_{Ph-b}), 119.6 (H_{Im-d}), 113.4 (H_{Ph-g}).

ESI-HRMS (m/z): calcd for $[(C_{12}H_{10}N_4Br)+H]^+$ 289.0089 found 289.0080

Elemental analysis (% calcd, % found for $C_{12}H_{10}N_4Br$): C (49.68, 49.71), H (3.47, 3.20), N (19.31, 19.31).

Synthesis of 1,1'-(5-bromo-1,3-phenylene)bis(3-methyl-1H-imidazol-3-ium) dibromide [HImPBr]Br$_2$

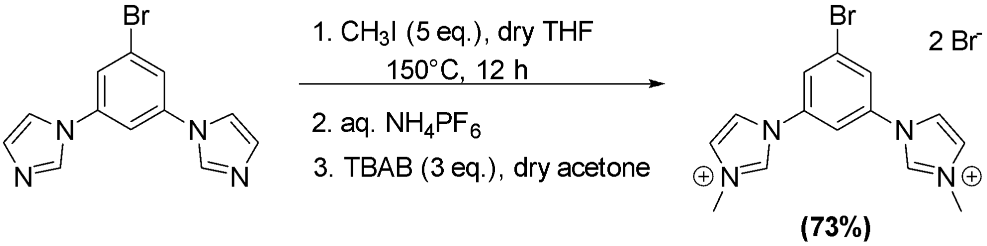

To a solution of 3,5-di(1H-imidazol-1-yl) bromobenzene (578 mg, 2 mmol) in dry THF (15 mL), in a pressure tube, methyl iodide (0.6 mL, 10 mmol) was added dropwise under N_2 flow. The resulting mixture was stirred and heated to 150°C for 12 h. After cooling it down to room temperature, diethyl ether was added to the suspension and the precipitate obtained was filtered on a sintered funnel (porosity 3). The precipitate was taken into the aq. NH_4PF_6 and further stirred for 15 mins. The precipitate was filtered and wash well with water and diethyl ether. The off-white solid was dried under vacuum and x-ray quality crystals were obtained for [HImPBr][PF$_6$]$_2$ from slow diffusion of diethyl ether into the acetonitrile solution of the compound. The compound was further dissolved in dry acetone (15 ml) followed by the addition of tetrabutylammonium bromide (1.93 g, 6 mmol), resulting into instant precipitation. The precipitate was filtered on a sintered funnel (porosity 3) and washed with dry acetone (3×10 mL). The compound was dried under vacuum and collected as off-white solid. Yield: 0.7 g (73%). R_f = 0.61 (Acetone/water/aq. KNO_3 : 8/3/1), M.P. 300-303°C (dec.).

^{1}H NMR (500 MHz, DMSO-d_6) δ 10.18 (s, 2H, H_{Im-g}), 8.55 (s, 2H, H_{Im-d}), 8.51 (s, 1H, H_{Ph-h}), 8.36 (d, J = 2.0 Hz, 2H, H_{Ph-b}), 8.04 (s, 2H, H_{Im-e}), 3.99 (s, 6H, H_{Im-f}).

^{13}C{^{1}H} NMR (126 MHz, DMSO-d_6) δ 136.8 (H_{Ph-c}), 136.5 (H_{Im-g}), 125.1 (H_{Ph-b}), 124.6 (H_{Im-e}), 123.6 (H_{Ph-a}), 120.9 (H_{Im-d}), 114.6 (H_{Ph-h}), 36.4 (H_{Im-f}).

ESI-HRMS (m/z): calcd for $[(C_{14}H_{15}N_4Br_3)+H]^+$ 476.8925 found 476.8932

Elemental analysis: (% calcd, % found for $C_{14}H_{15}N_4Br_3$): C (35.10, 35.09), H (3.16, 3.16), N (11.70, 11.68).

Synthesis of 2,6-bis(3-methylimidazol-2-ylidene-1-yl)(4-bromophen-1-yl) iron(II) hexafluorophosphate *[Fe(ImPBr)$_2$]PF$_6$*

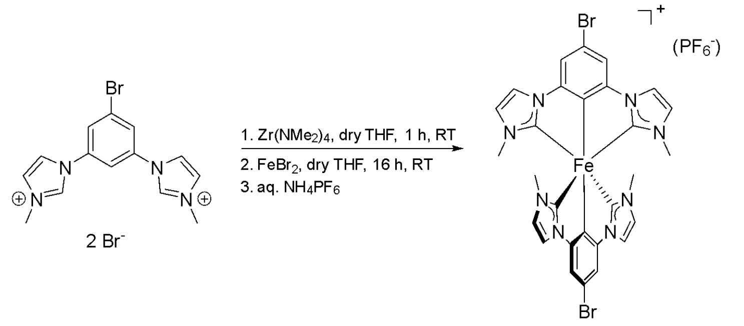

(41%)

A Schlenk flask was charged with 1,1'-(5-bromo-1,3-phenylene)bis(3-methyl-1H-imidazol-3-ium) dibromide (479 mg, 1 mmol) and tetrakis(dimethylamido)zirconium(IV) (668 mg, 2.5 mmol) in the glove box under N_2 flow. Dry THF (20 ml) was added to the Schlenk flask, and the mixture was stirred for 1 h under N_2 flow at room temperature. Iron (II) bromide (130 mg, 0.6 mmol) was dissolved in the dry THF (10 ml) which was then added to the yellow suspension. The resulting dark green suspension was stirred under N_2 for 16 h. The reaction was quenched with 2 mL methanol and reaction mixture was exposed to air while stirring for another 1 h. The reaction mixture was filtered through a sintered funnel (porosity 3) using suction and washed with acetonitrile until color disappears from the frit. The filtrate was evaporated under vacuo to give dark green solid. The solid was reprecipitated by pouring into aq. NH_4PF_6 (650 mg in 15 mL water). The dark green precipitate was filtered, washed with water, and redissolved in acetonitrile. The complex was isolated and purified using silica column chromatography (Acetone/water/aq. KNO_3 : 8/3/1). The fraction was collected and evaporated under vacuum followed by the precipitation from aq. NH_4PF_6 (320 mg in 20 mL). The solid obtained were washed well with water, hexane, and diethyl ether. Later, the complex was passed through size exclusion chromatography (CH_3CN/Toluene: 1/1) and the filtrate evaporated to yield the pure Fe (III) complex. X-ray quality crystals were obtained by the slow vapor diffusion of diethyl ether into the acetonitrile solution. Yield: 170 mg (41%). R_f = 0.86 (Acetone/water/aq. KNO_3 : 8/3/1), M.P. 265-268°C (dec.).

^{1}H NMR (600 MHz, CD_3CN) δ 25.35 (s, 4H, H_{Ph-a}), 10.37 (s, 12H, H_{Im-d}), 2.86 (s, 4H, H_{Im-c}), -3.04 (s, 4H, H_{Im-b}).

^{13}C{^{1}H} NMR (151 MHz, CD_3CN) δ 562.7 (H_{Ph-c}), 513.1 (H_{Ph-a}), 89 (H_{Im-d}), 77.5 (H_{Im-e}), 1.4 (H_{Im-f}), -202.5 (H_{Ph-b}).

ESI-HRMS (m/z): calcd for $[(C_{28}H_{24}FeN_8Br_2PF_6)-PF_6]^+$ 685.9840 found 685.9850

Elemental analysis: (% calcd, % found for $C_{28}H_{24}FeN_8Br_2PF_6$): C (40.36, 40.31), H (2.90, 2.89), N (13.45, 13.43).

S2.2 Synthesis of [Bis(2,6-bis(3-methylimidazol-2-ylidene-1-yl)4-(furan-2-yl)phen-4 yl) iron(III)] hexafluorophosphate, $[Fe(ImPFur)_2]PF_6$

Synthesis of 1,1'-(5-(furan-2-yl)-1,3-phenylene)bis(1H-imidazole)

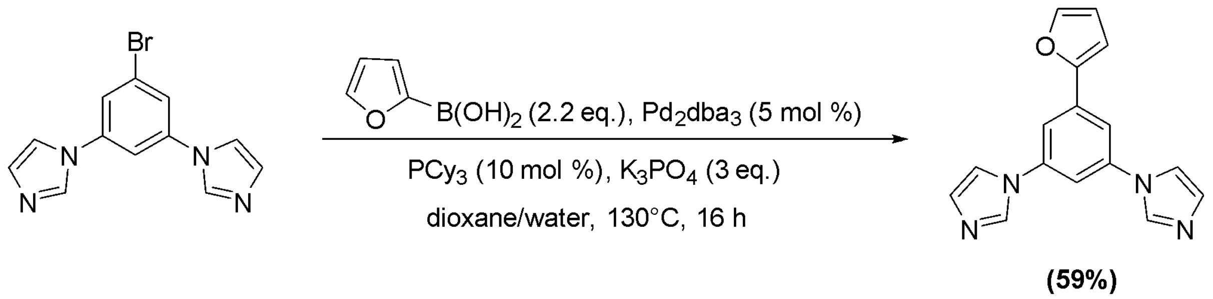

A solution of potassium phosphate (636 mg, 3 mmol) in dioxane/water (1:1, 10 ml), was purged with N_2 while stirring for 1 h. A pressure tube was charged with 2-Furanylboronic acid (246 mg, 2.2 mmol), tris(dibenzylideneacetone)dipalladium (0) (46 mg, 5 mol %) tricyclohexylphosphine (28 mg, 10 mol %), and 1,1'-(5-bromo-1,3-phenylene)bis(1*H*-imidazole) (289 mg, 1 mmol) under N_2 flow. The potassium phosphate solution was added to the pressure tube and the resulting mixture was stirred and heated to 130°C for 16 h before cooling it down to room temperature. Pale greenish suspension was filtered through celite pad using suction and washed with ethyl acetate. The organic phase was washed with water and brine and later dried over Na_2SO_4. The organic phase was evaporated under vacuum, resulting into off-white solid. The compound was dried under vacuum and collected. Yield: 163 mg (59%). R_f = 0.75 (Methanol / DCM : 1/9), M.P. 172-175°C.

^{1}H NMR (500 MHz, CD_3OD) δ 8.32 (s, 1H, H_{Im-j}), 7.85 (d, *J* = 2.0 Hz, 2H, H_{Ph-f}), 7.74 (t, *J* = 1.4 Hz, 2H, H_{Im-i}), 7.70 (t, *J* = 2.1 Hz, 1H, H_{Ph-k}), 7.64 (d, *J* = 2.4 Hz, 1H, H_{Fur-c}), 7.21 – 7.15 (m, 2H, H_{Im-h}), 7.08 (d, *J* = 3.8 Hz, 1H, H_{Fur-a}), 6.58 (dd, *J* = 3.5, 1.8 Hz, 1H, H_{Fur-b}).

^{13}C{^{1}H} NMR (126 MHz, CD_3OD) δ 152.8 (H_{Fur-d}), 144.9 (H_{Fur-c}), 140.3 (H_{Ph-g}), 137.2 (H_{Im-j}), 135.6 (H_{Ph-e}), 130.5 (H_{Im-h}), 119.7 (H_{Im-i}), 115.4 (H_{Ph-f}), 113.3 (H_{Fur-b}), 112.9 (H_{Ph-k}), 108.3 (H_{Fur-a}).

ESI-HRMS (m/z): calcd for $[(C_{16}H_{12}N_4O)+H]^+$ 277.1089 found 277.1089

Elemental analysis (% calcd, % found for $C_{16}H_{12}N_4O$): C (69.55, 68.57), H (4.38, 4.56), N (20.28, 19.94).

Synthesis of 1,1'-(5-(furan-2-yl)-1,3-phenylene)bis(3-methyl-1H-imidazol-3-ium) dibromide [HImPFur]Br$_2$

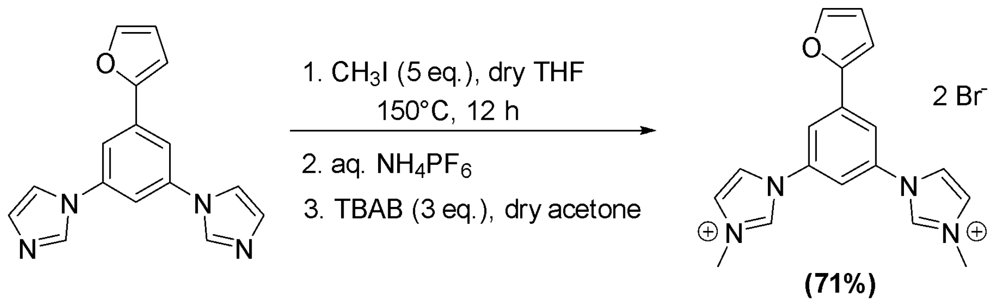

To a solution of 1,1'-(5-(furan-2-yl)-1,3-phenylene)bis(1H-imidazole) (276 mg, 1 mmol) in dry THF (10 mL), in a pressure tube, methyl iodide (0.3 mL, 5 mmol) was added dropwise under N_2 flow. The resulting mixture was stirred and heated to 150°C for 12 h. After cooling it down to room temperature, diethyl ether was added to the suspension and the precipitate obtained was filtered on a sintered funnel (porosity 3). The precipitate was taken into the aq. NH_4PF_6 and further stirred for 15 mins. The precipitate was filtered and wash well with water and diethyl ether. The pale-yellow solid was dried under vacuum and x-ray quality crystals were obtained for $[HImPFur][PF_6]_2$ from slow diffusion of diethyl ether into the acetonitrile solution of the compound. The compound was further dissolved in dry acetone (15 ml) followed by the addition of tetrabutylammonium bromide (966 mg, 3 mmol), resulting into instant precipitation. The precipitate was filtered on a sintered funnel (porosity 3) and washed with dry acetone (3×10 mL). The compound was dried

under vacuum and collected as pale-yellow solid. Yield: 330 mg (71%). R_f = 0.64 (Acetone/water/aq. KNO_3 : 8/3/1), M.P. 250-253°C (dec.).

^{1}H NMR (500 MHz, DMSO-*d_6*) δ 10.14 (s, 2H, H_{Im-k}), 8.53 (s, 2H, H_{Im-h}), 8.33 (s, 2H, H_{Ph-f}), 8.28 (s, 1H, H_{Ph-l}), 8.05 (s, 2H, H_{Im-i}), 7.98 (s, 1H, H_{Fur-c}), 7.38 (d, *J* = 3.5 Hz, 1H, H_{Fur-a}), 6.78 (s, 1H, H_{Fur-b}), 4.01 (s, 6H, H_{Im-j}).

^{13}C{^{1}H} NMR (126 MHz, DMSO-*d_6*) δ 150.3 (H_{Fur-d}), 144.9 (H_{Fur-c}), 136.6 (H_{Im-k}), 136.4 (H_{Ph-g}), 133.6 (H_{Ph-e}), 124.6 (H_{Im-i}), 120.9 (H_{Im-h}), 116.7 (H_{Ph-f}), 113.4 (H_{Ph-l}), 112.9 (H_{Fur-b}), 109.9 (H_{Fur-a}), 36.4 (H_{Im-j}).

ESI-HRMS (m/z): calcd for $[(C_{18}H_{18}N_4Br_2O)+H]^+$ 464.9926 found 464.9920

Elemental analysis: (% calcd, % found for $C_{16}H_{18}N_4Br_2O_2$): C (46.38, 45.91), H (3.89, 3.95), N (12.02, 11.91).

Synthesis of 2,6-bis(3-methylimidazol-2-ylidene-1-yl)(4-(furan-2-yl)-phen-1-yl) iron(II) hexafluorophosphate *[Fe(ImPFur)$_2$]PF$_6$*

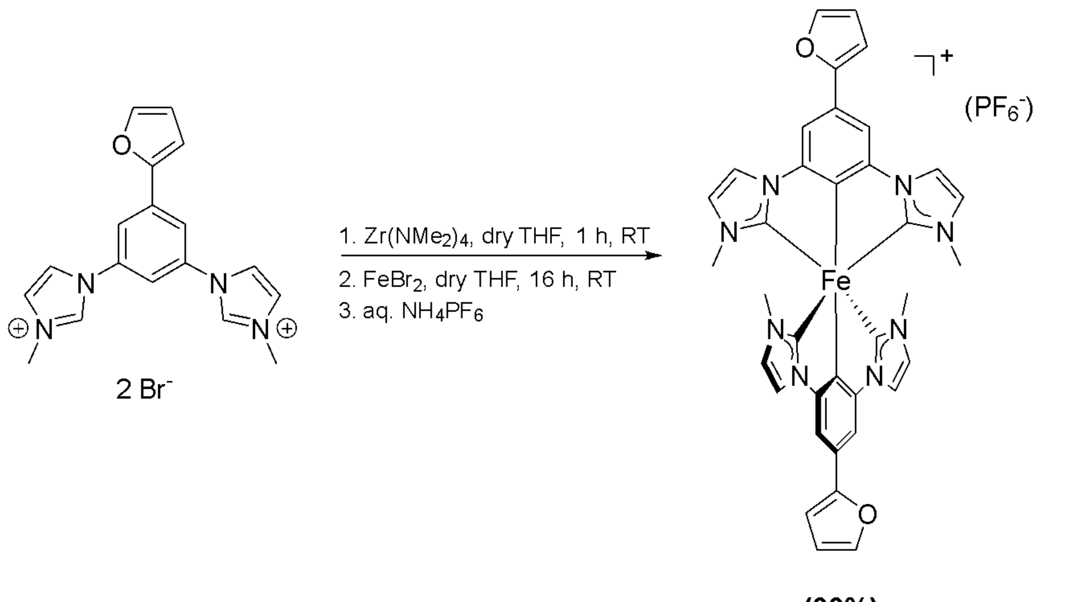

(33%)

A Schlenk flask was charged with 1,1'-(5-(furan-2-yl)-1,3-phenylene)bis(3-methyl-1H-imidazol-3-ium) dibromide (466 mg, 1 mmol) and tetrakis(dimethylamido)zirconium(IV) (668 mg, 2.5 mmol) in the glove box under N_2 flow. Dry THF (10 ml) was added to the Schlenk flask, and the mixture was stirred for 1 h under N_2 flow at room temperature. Iron (II) bromide (130 mg, 0.6 mmol) was dissolved in the dry THF (10 ml) which was then added to the yellow suspension. The resulting orange suspension was stirred under N_2 for 16 h. The reaction was quenched with 2 mL methanol and reaction mixture was exposed to air while stirring for another 1 h. The reaction mixture turned dark green which was then filtered through a sintered funnel (porosity 3) using suction and washed with acetonitrile until colour disappears from the frit. The filtrate was evaporated under vacuo (at 30°C) to give dark green solid. The solid was reprecipitated by pouring into aq. NH_4PF_6 (650 mg in 15 mL water). The dark green precipitate was filtered, washed with water, and redissolved in acetonitrile. The complex was isolated and purified using silica column chromatography (Acetone/water/aq. KNO_3: 8/3/1). The fraction was collected and evaporated under vacuum followed by the precipitation from aq. NH_4PF_6 (320 mg in 20 mL). The solid obtained were washed well with water, hexane, and diethyl ether. Later, the complex was passed through size exclusion chromatography (CH_3CN/Toluene: 1/1) and the filtrate evaporated to yield the pure Fe (III) complex. X-ray quality crystals were obtained by the slow vapor diffusion of diethyl ether into the acetonitrile solution. Yield: 133 mg (33%). R_f = 0.53 (Acetone/water/aq. KNO_3 : 8/3/1), M.P. 275-278°C (dec.).

^{1}H NMR (600 MHz, CD_3CN) δ 20.28 (s, 4H, H_{Ph-c}), 12.52 (s, 12H, H_{Im-f}), 7.94 (s, 12H, H_{Am-a}), 6.0 (s, 12H, H_{Am-b}), 2.47 (s, 4H, H_{Im-e}), -5.08 (s, 4H, H_{Im-d}).

^{13}C{^{1}H} NMR (151 MHz, CD_3CN) δ 485.8 (H_{Ph-f}), 452.5 (H_{Ph-d}), 90.0 (H_{Im-g}), 83.9 (H_{Im-h}), 59.6 (H_{Am-a}), 58.9, 44.5 (H_{Am-b}), 1.9 (H_{Im-i}), -171.5 (H_{Ph-e}).

ESI-HRMS (m/z): calcd for $[(C_{36}H_{30}FeN_8O_2PF_6)\text{-}PF_6]^+$ 663.1919 found 663.1857

Elemental analysis: (% calcd, % found for $C_{36}H_{30}FeN_8O_2PF_6$): C (53.55, 53.74), H (3.74, 3.79), N (13.88, 13.51).

S3. NMR Spectra

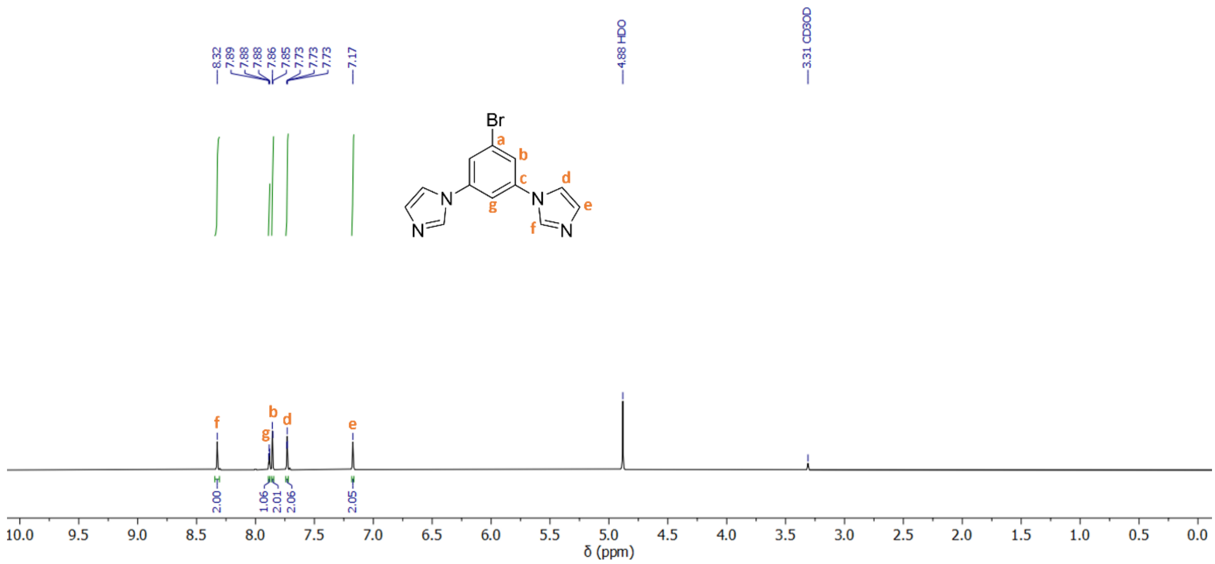

Figure S1: ^{1}H NMR spectrum of 1,1'-(5-bromo-1,3-phenylene)bis(1*H*-imidazole) (20 mM) in CD_3OD.

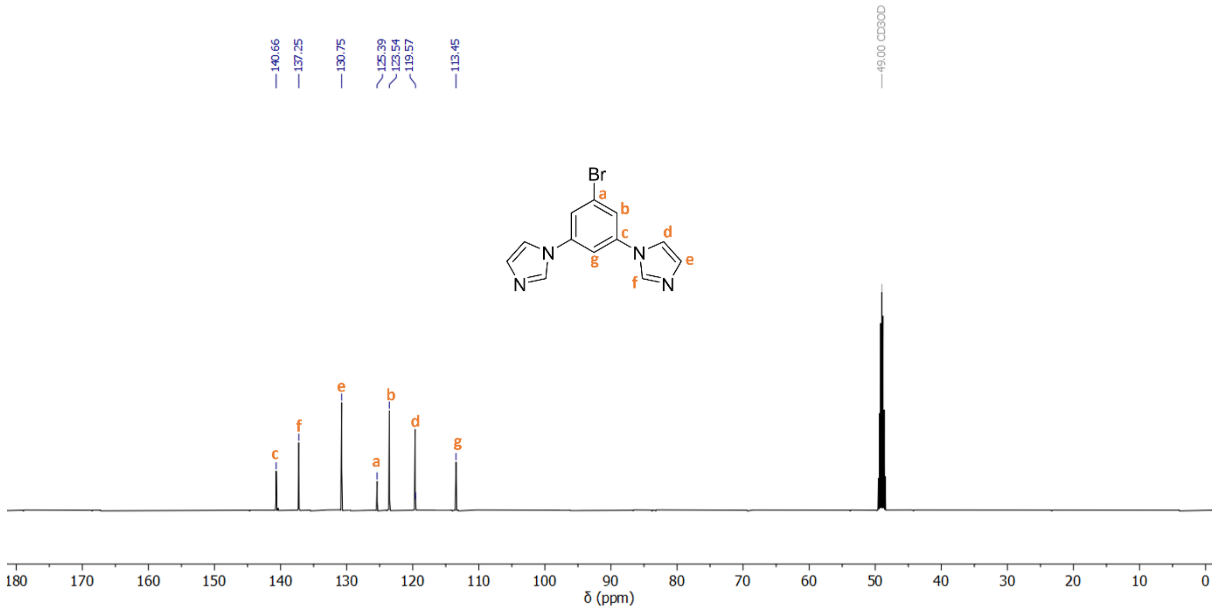

Figure S2: ^{13}C NMR spectrum of 1,1'-(5-bromo-1,3-phenylene)bis(1*H*-imidazole) (20 mM) in CD_3OD.

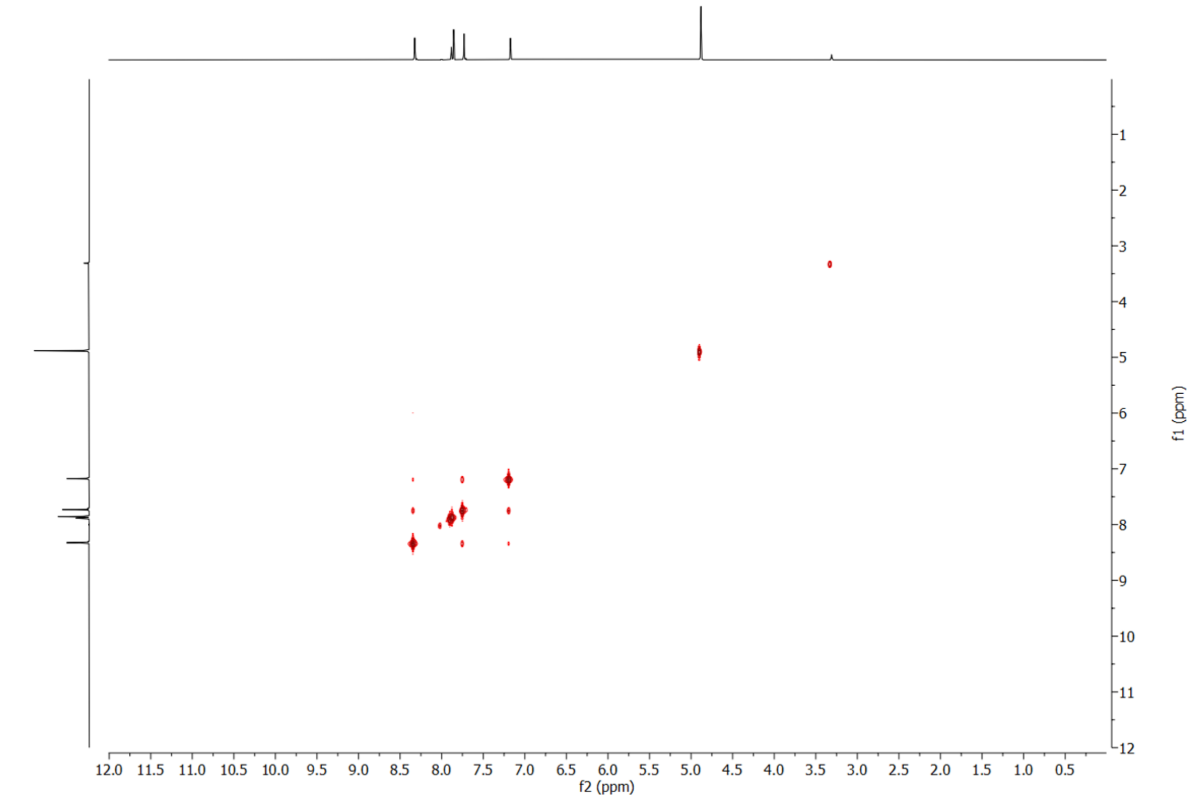

Figure S3: COSY spectrum of 1,1'-(5-bromo-1,3-phenylene)bis(1*H*-imidazole*)* in CD_3OD (500 MHz, 500 MHz).

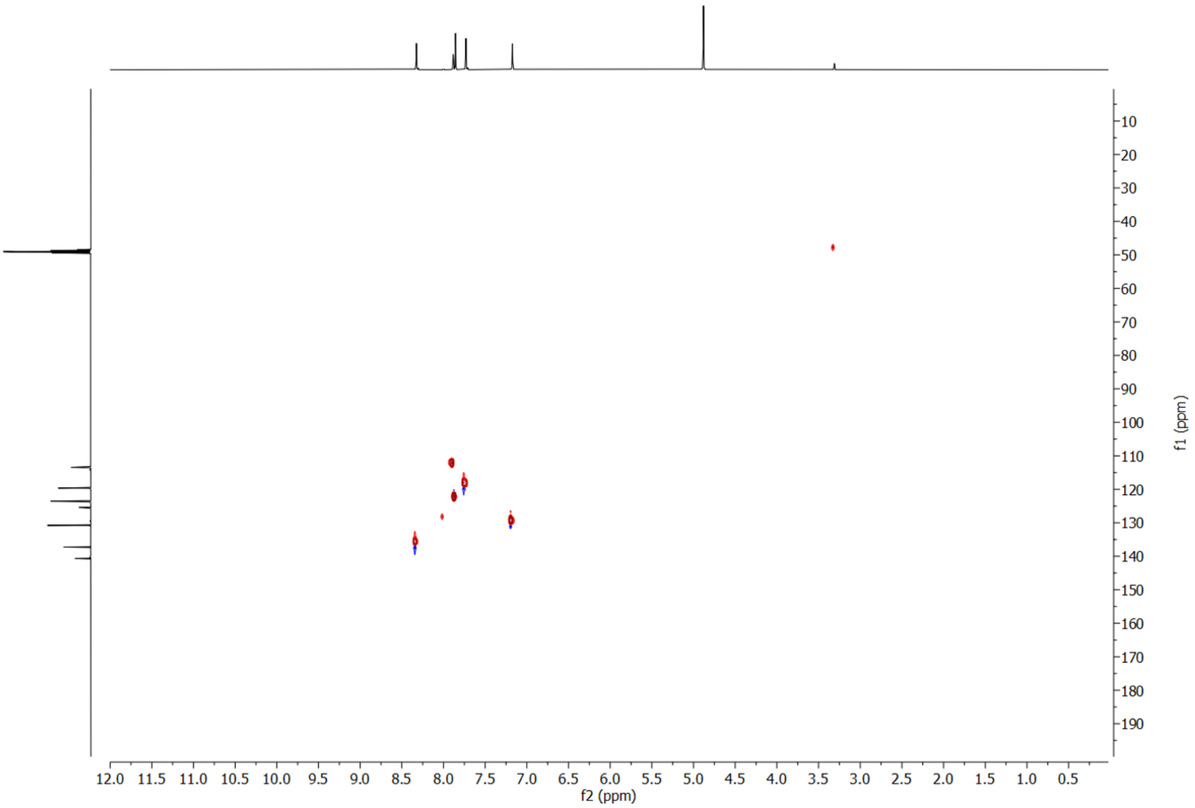

Figure S4: HSQC spectrum of 1,1'-(5-bromo-1,3-phenylene)bis(1*H*-imidazole) in CD_3OD (500 MHz, 126 MHz).

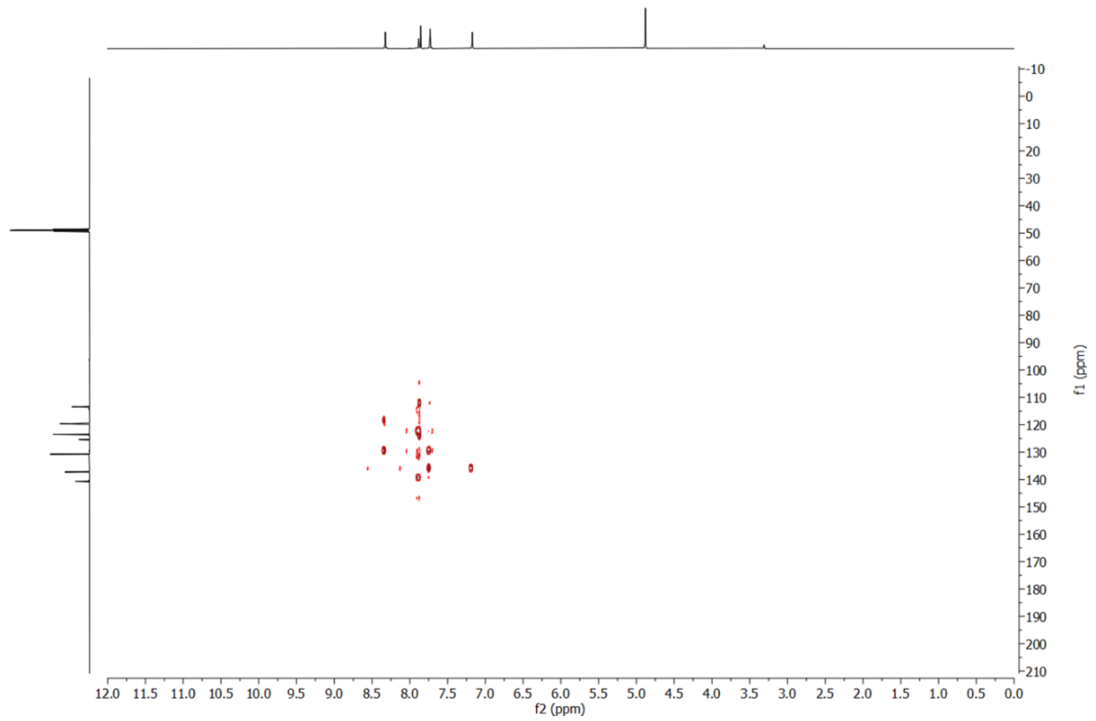

Figure S5: HMBC spectrum of 1,1'-(5-bromo-1,3-phenylene)bis(1H-imidazole) in CD_3OD (500 MHz, 126 MHz).

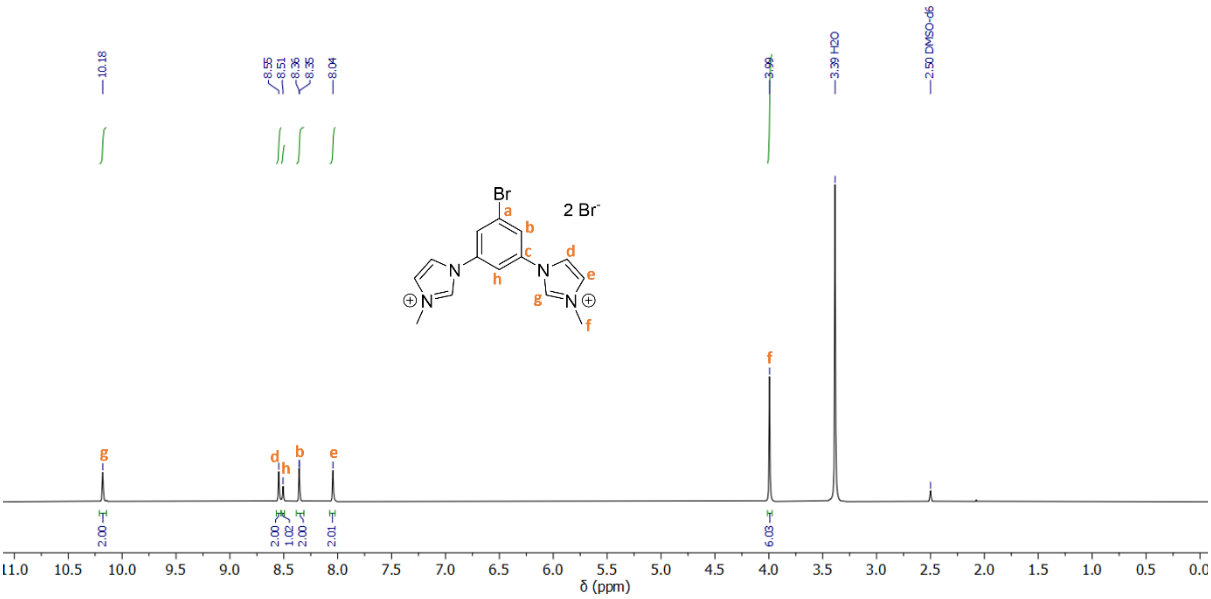

Figure S6: ^{1}H NMR spectrum of $[HImPBr]Br_2$ (15 mM) in DMSO-d_6.

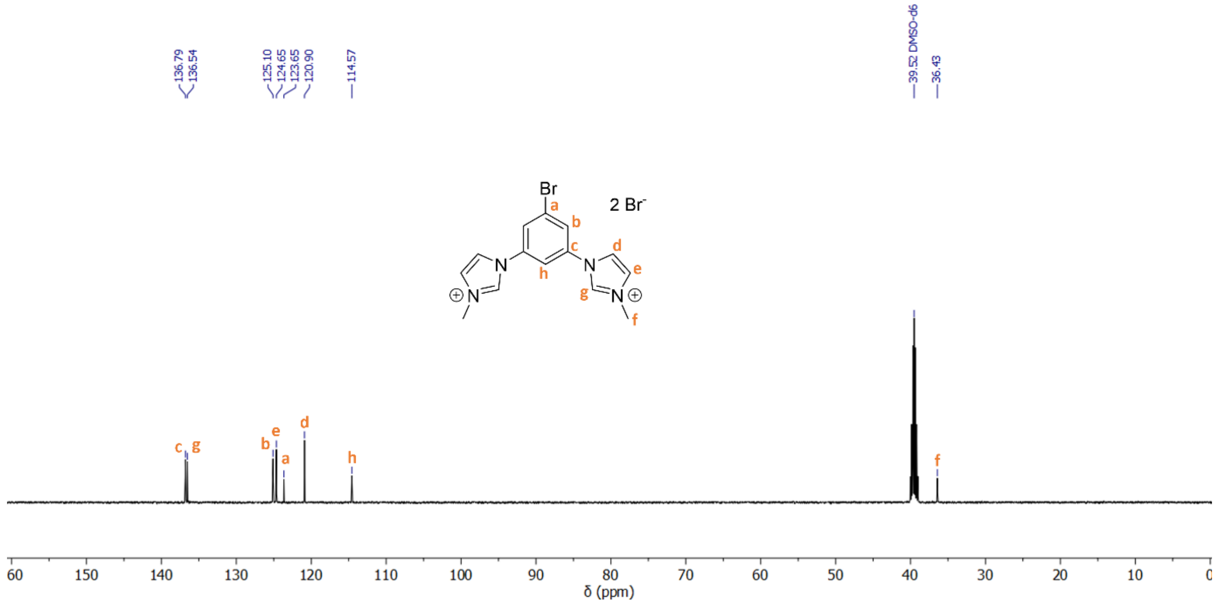

Figure S7: ^{13}C NMR spectrum of $[HImPBr]Br_2$ (15 mM) in DMSO-d_6.

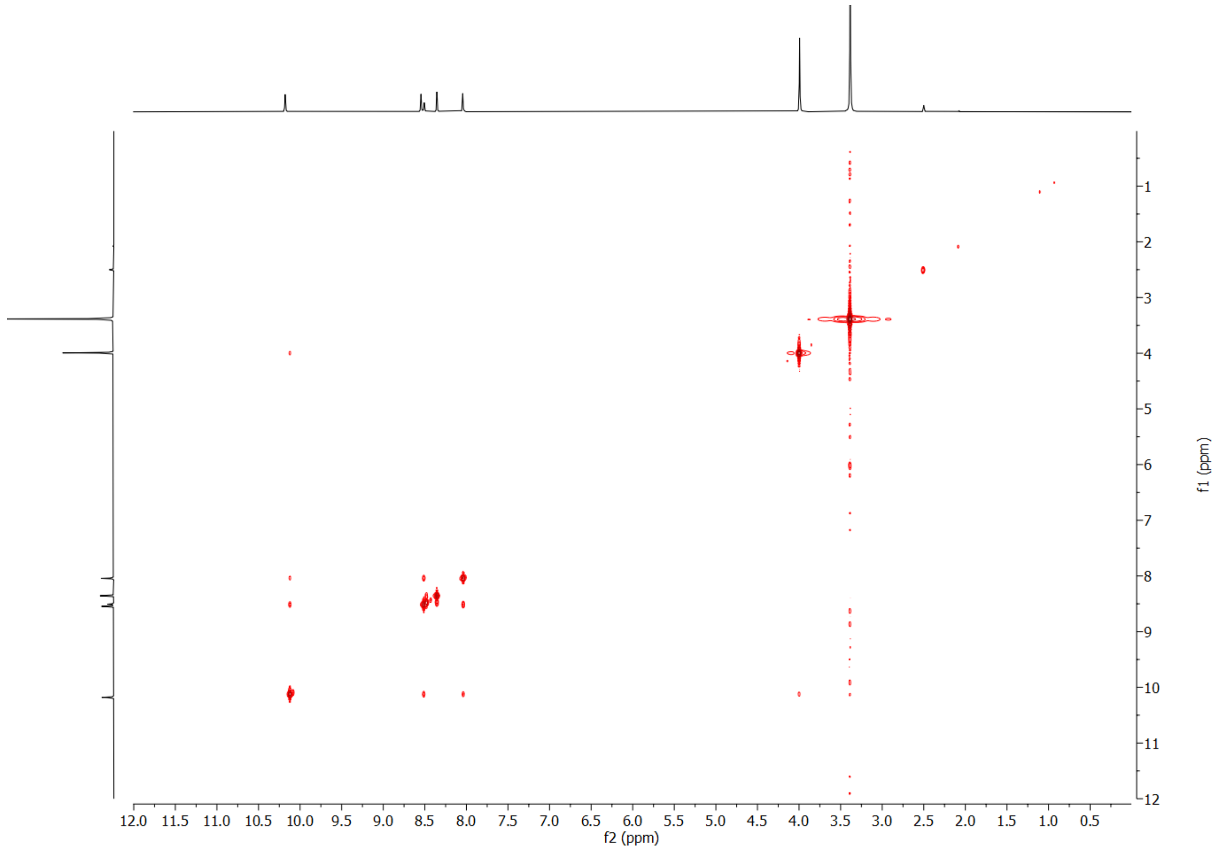

Figure S8: COSY spectrum of $[HImPBr]Br_2$ in DMSO-d_6 (500 MHz, 500 MHz).

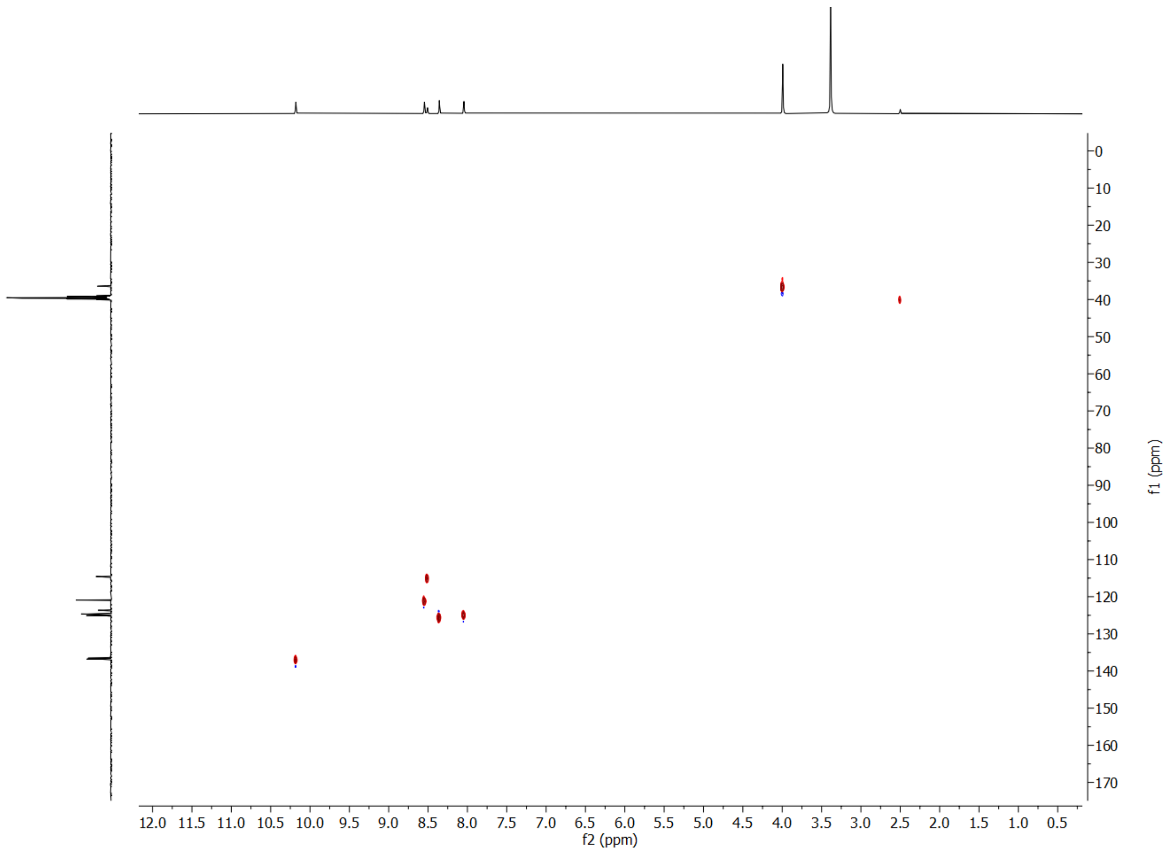

Figure S9: HSQC spectrum of $[HImPBr]Br_2$ in DMSO-d_6 (500 MHz, 126 MHz).

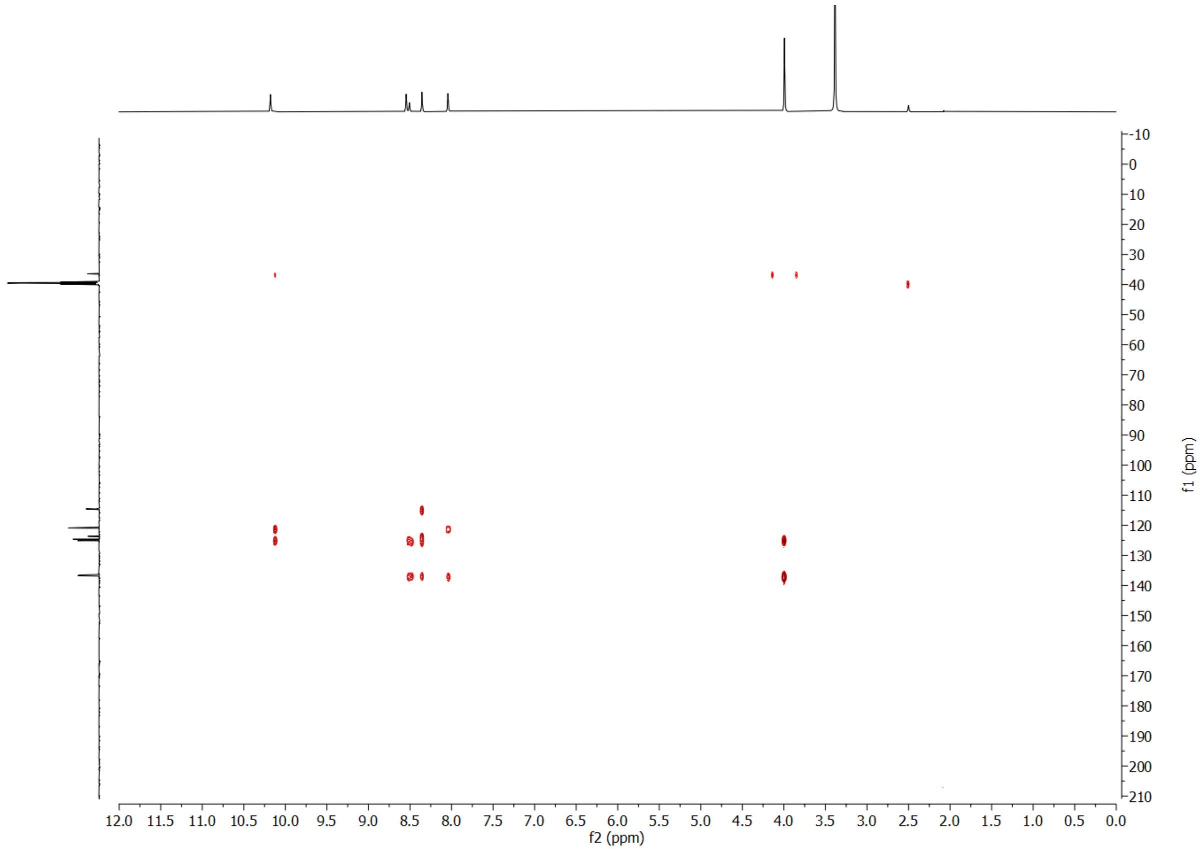

Figure S10: HMBC spectrum of $[HImPBr]Br_2$ in DMSO-d_6 (500 MHz, 126 MHz).

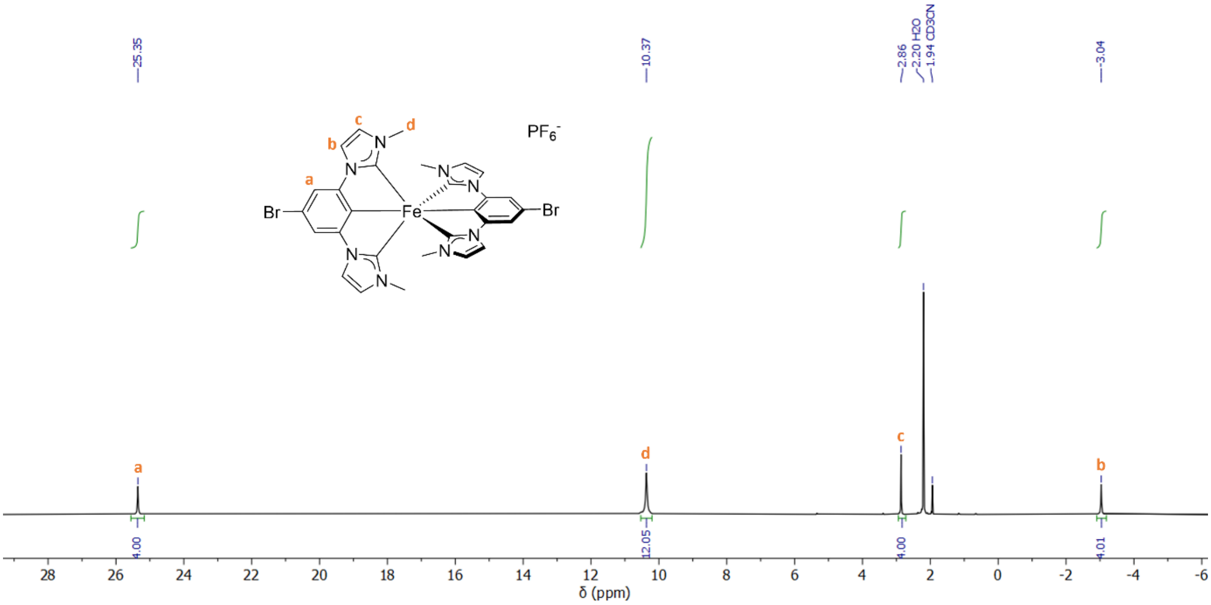

Figure S11: ^{1}H NMR spectrum of $[Fe(ImPBr)_2]PF_6$ (35 mM) in CD_3CN.

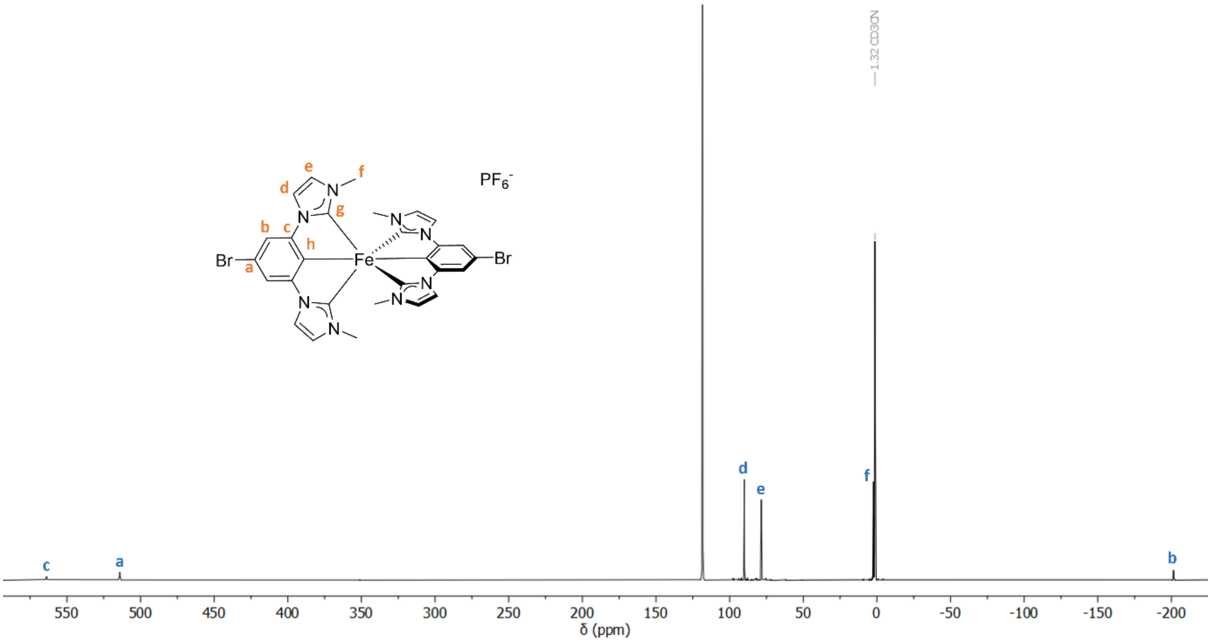

Figure S12: ^{13}C NMR spectrum of $[Fe(ImPBr)_2]PF_6$ (35 mM) in CD_3CN.

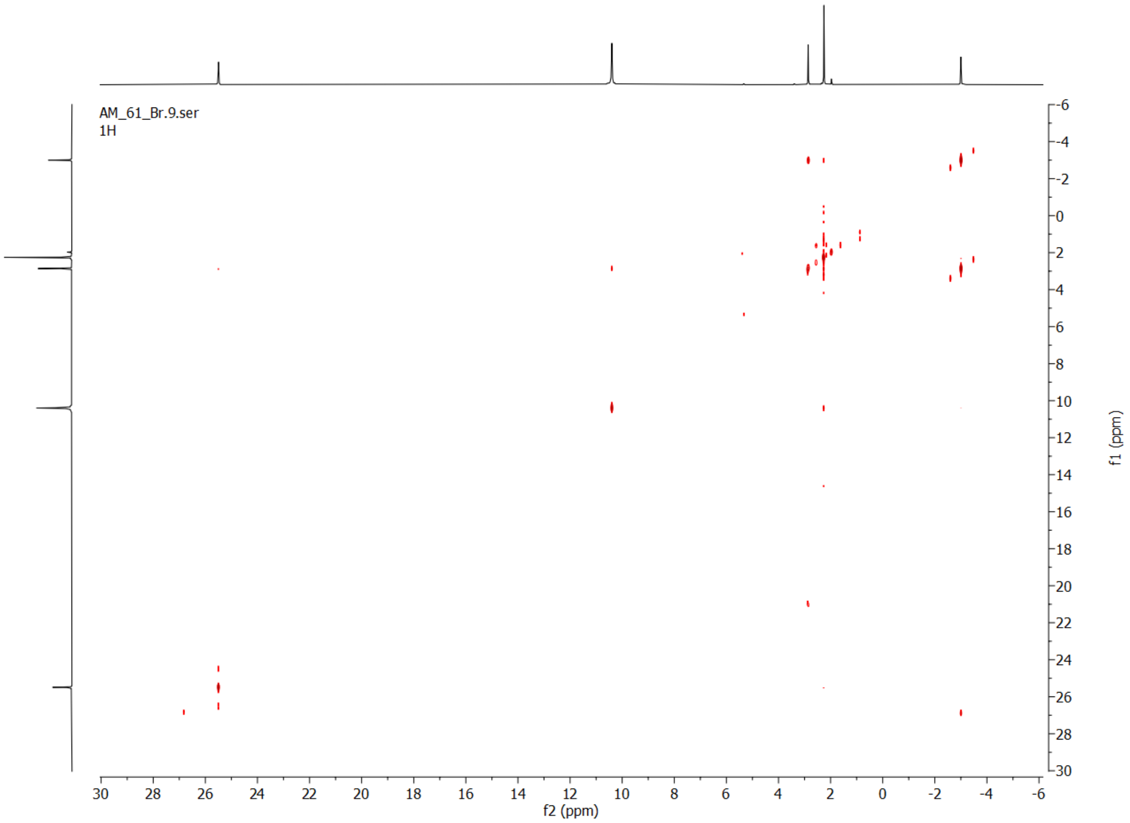

Figure S13: COSY spectrum of $[Fe(ImPBr)_2]PF_6$ in CD_3CN (600 MHz, 151 MHz).

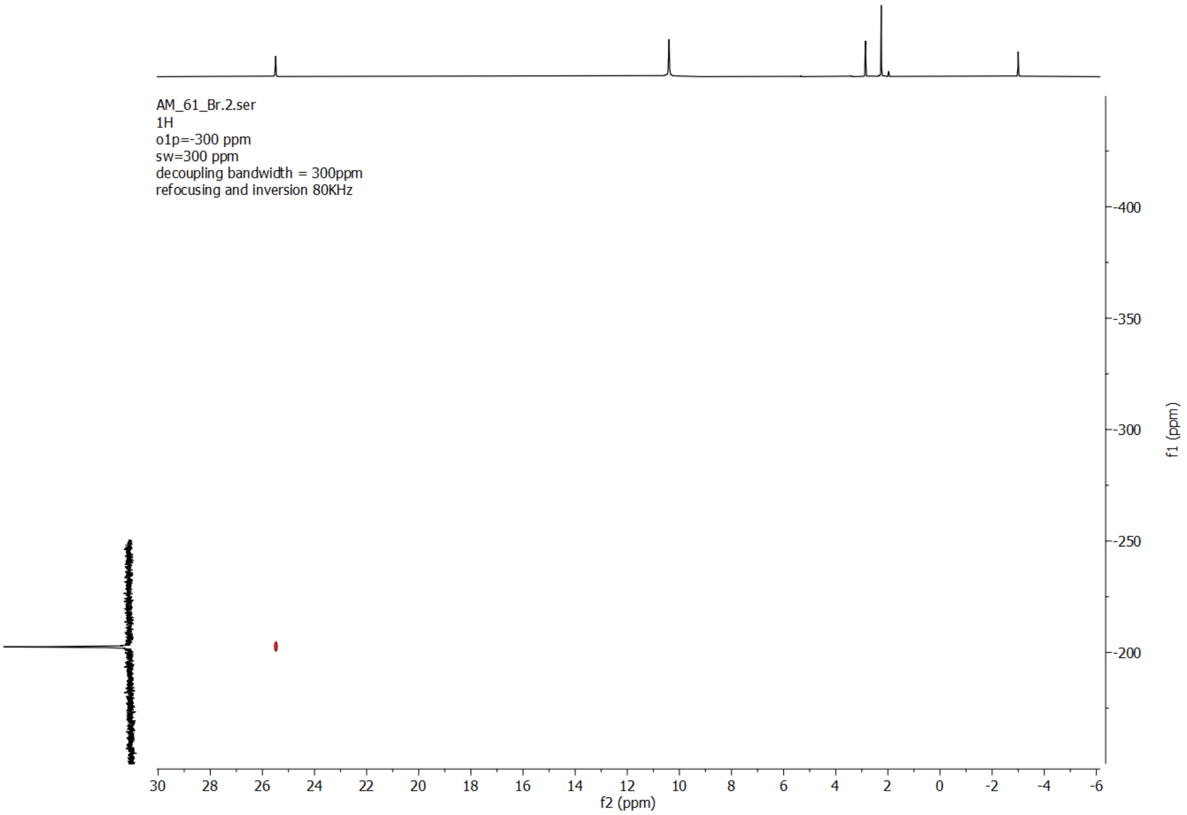

Figure S14: HSQC spectrum of $[Fe(ImPBr)_2]PF_6$ in CD_3CN (600 MHz, 151 MHz).

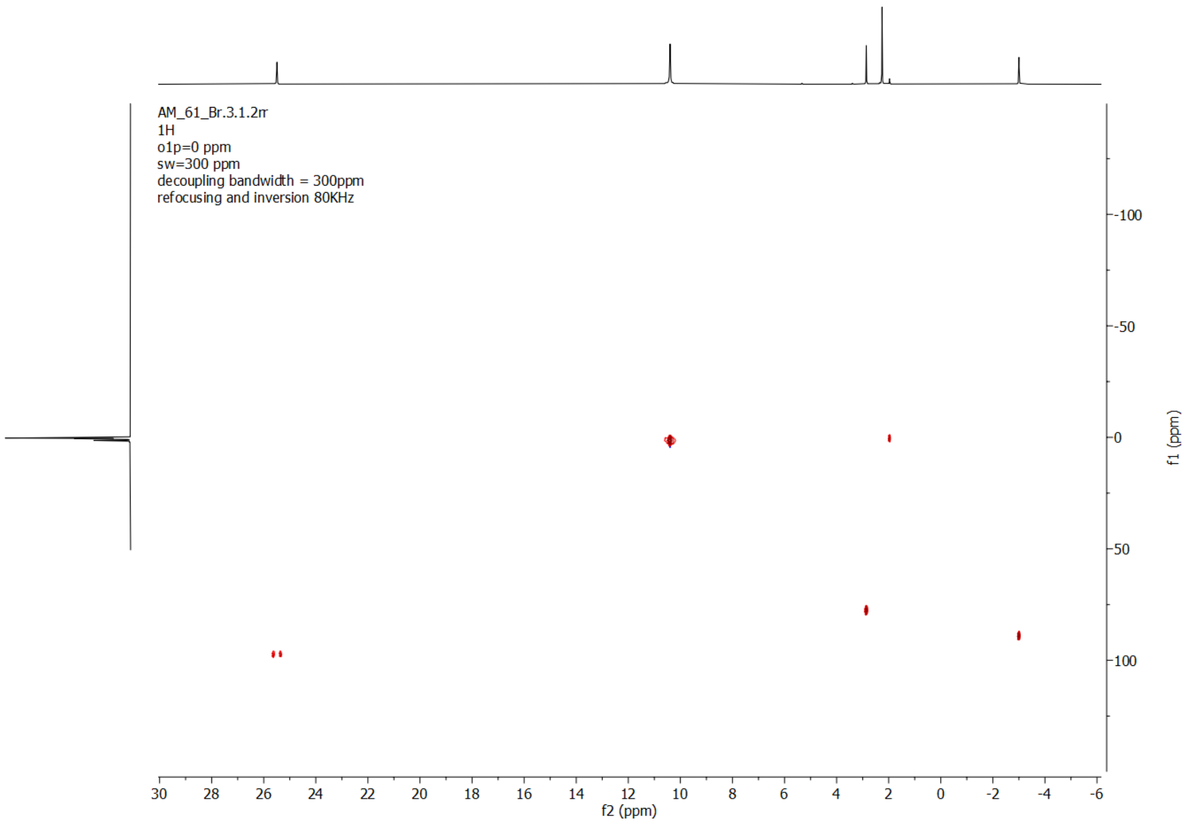

Figure S15: HSQC spectrum of $[Fe(ImPBr)_2]PF_6$ in CD_3CN (600 MHz, 151 MHz).

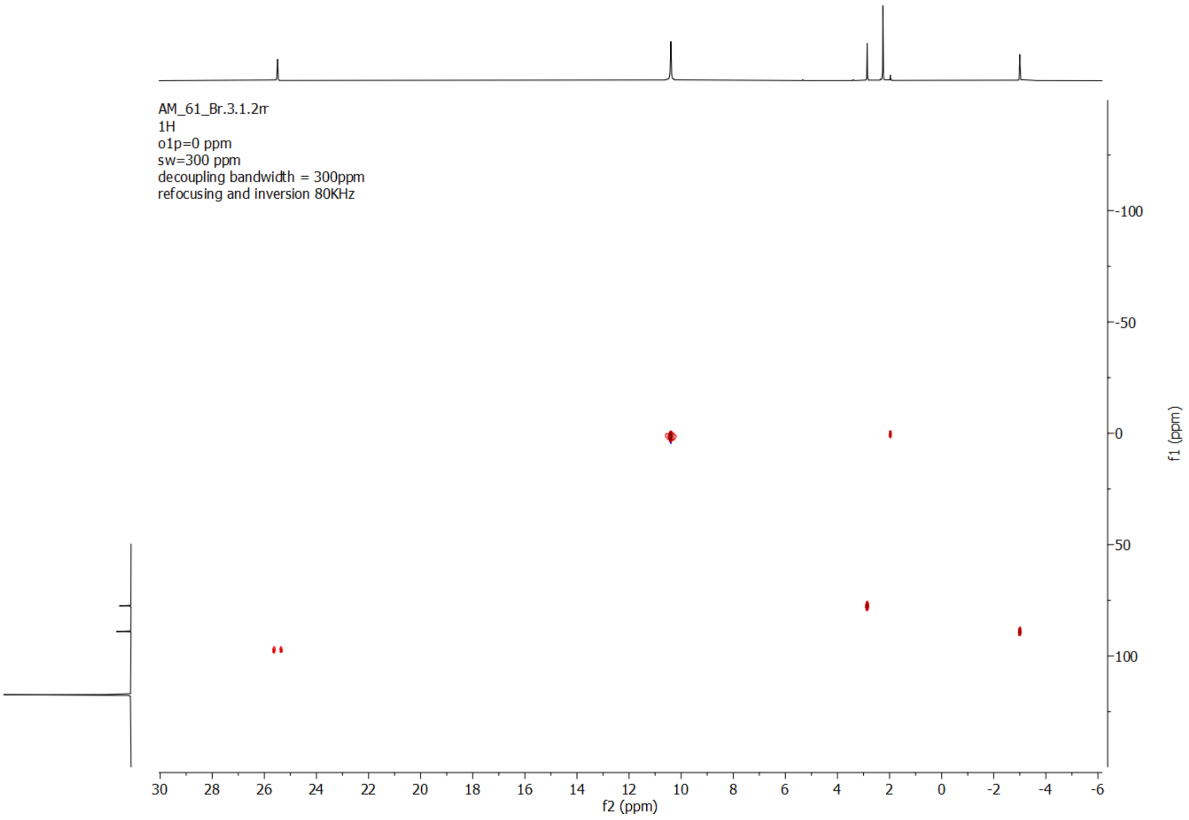

Figure S16: HSQC spectrum of $[Fe(ImPBr)_2]PF_6$ in CD_3CN (600 MHz, 151 MHz).

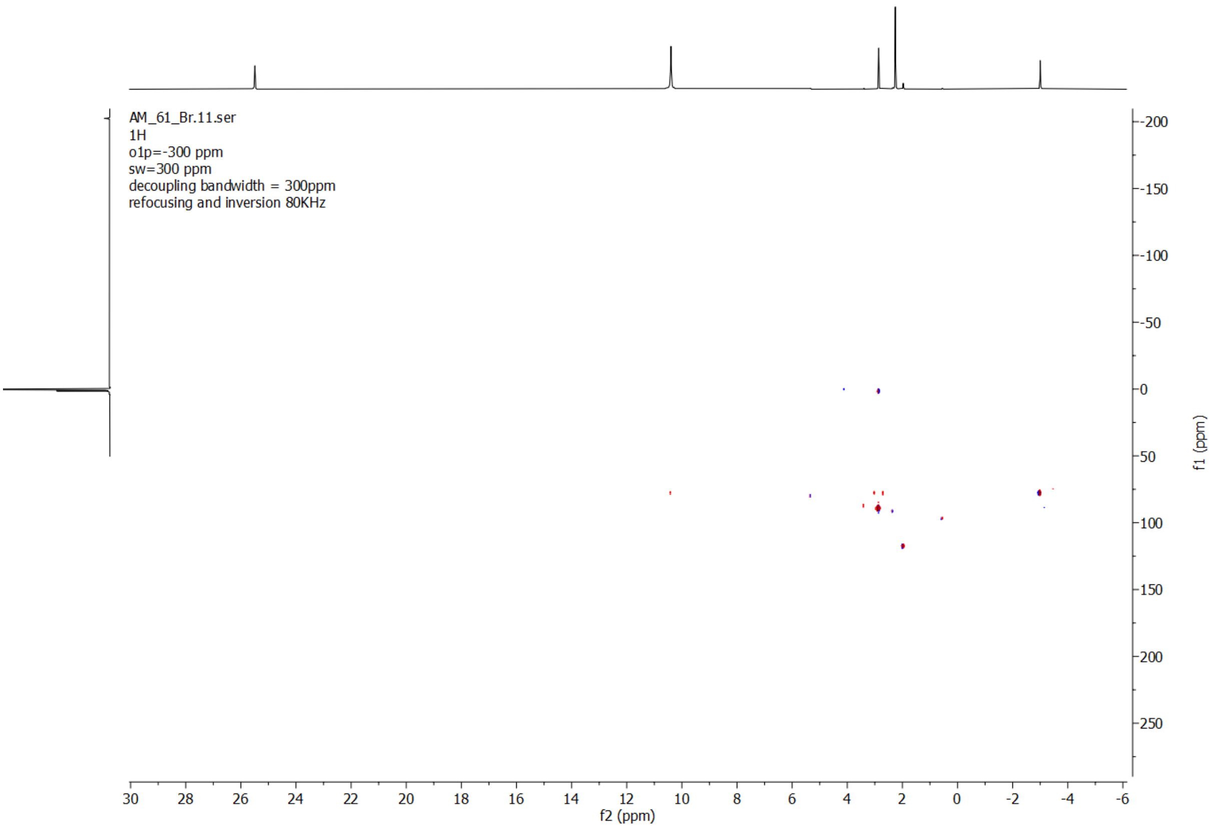

Figure S17: HMBC spectrum of $[Fe(ImPBr)_2]PF_6$ in CD_3CN (600 MHz, 151 MHz).

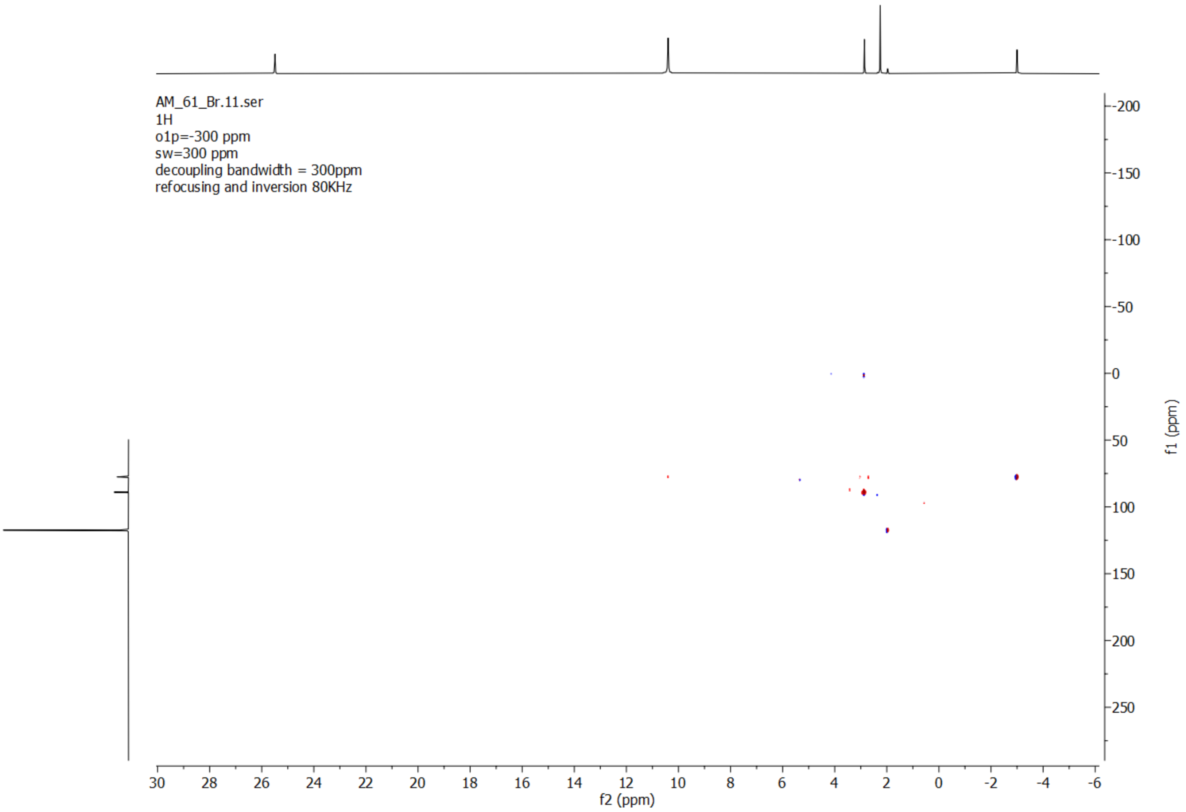

Figure S18: HMBC spectrum of $[Fe(ImPBr)_2]PF_6$ in CD_3CN (600 MHz, 151 MHz).

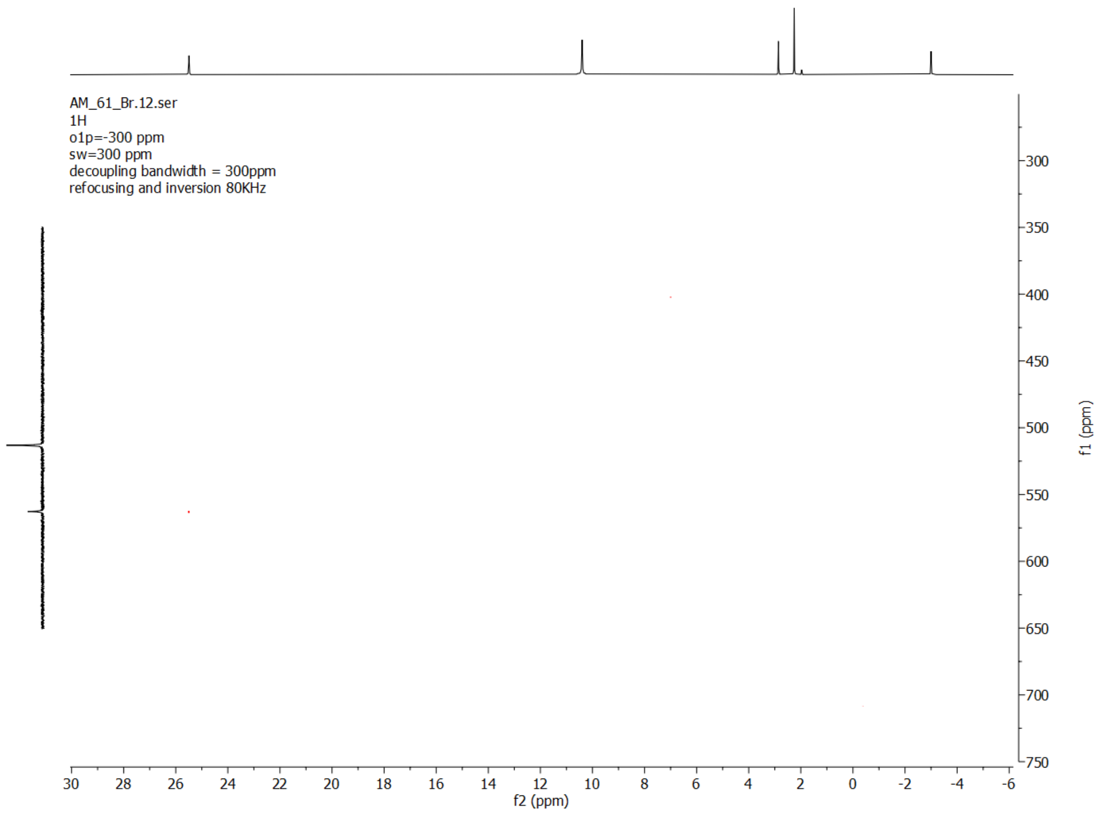

Figure S19: HMBC spectrum of $[Fe(ImPBr)_2]PF_6$ in CD_3CN (600 MHz, 151 MHz).

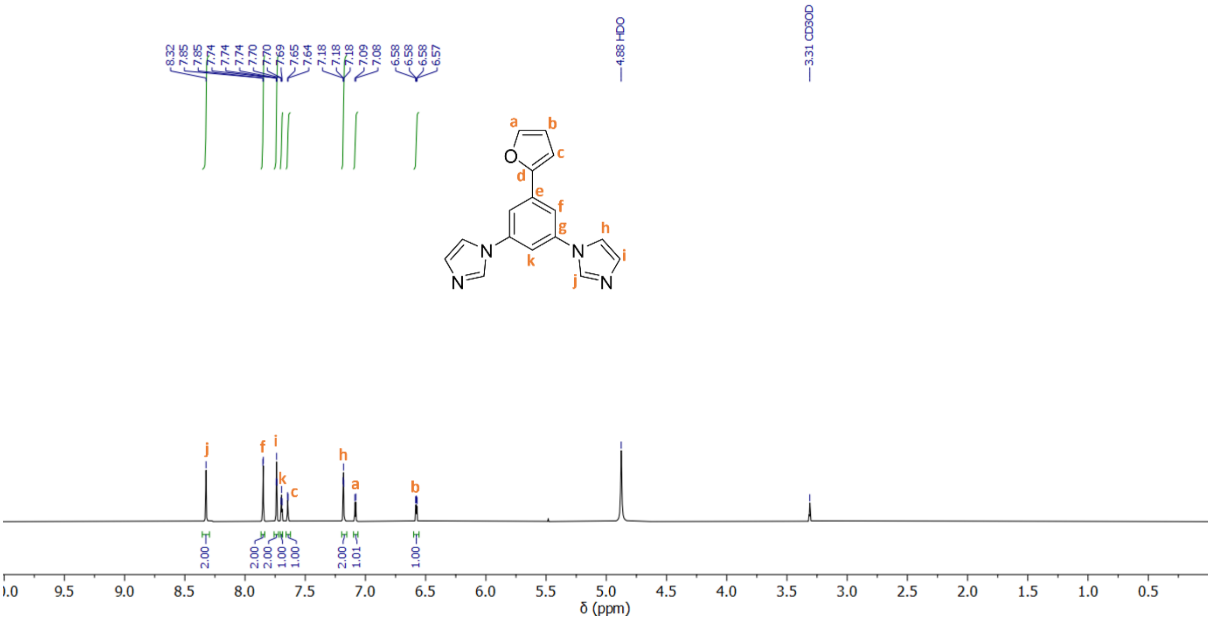

Figure S20: ^{1}H NMR spectrum of *of* 1,1'-(5-(furan-2-yl)-1,3-phenylene)bis(1*H*-imidazole) (20 mM) in CD_3OD.

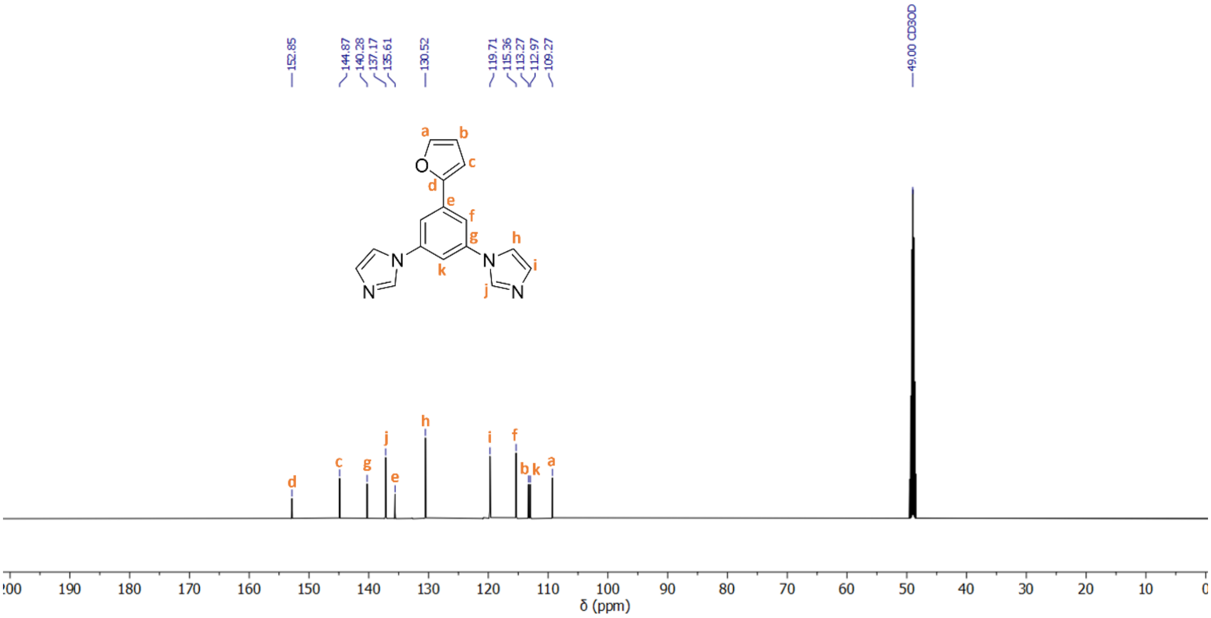

Figure S21: ^{13}C NMR spectrum of 1,1'-(5-(furan-2-yl)-1,3-phenylene)bis(1*H*-imidazole) (20 mM) in CD_3OD.

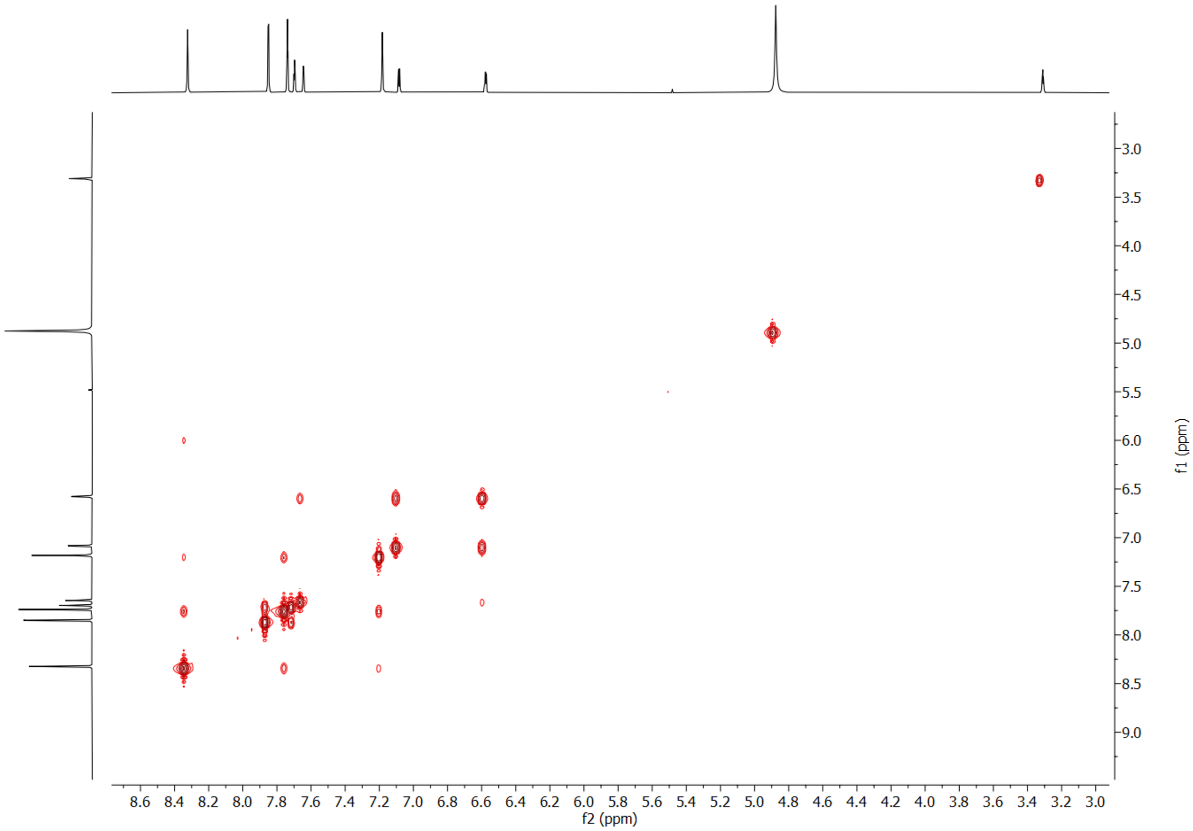

Figure S22: COSY spectrum of 1,1'-(5-(furan-2-yl)-1,3-phenylene)bis(1*H*-imidazole) in CD_3OD (500 MHz, 500 MHz).

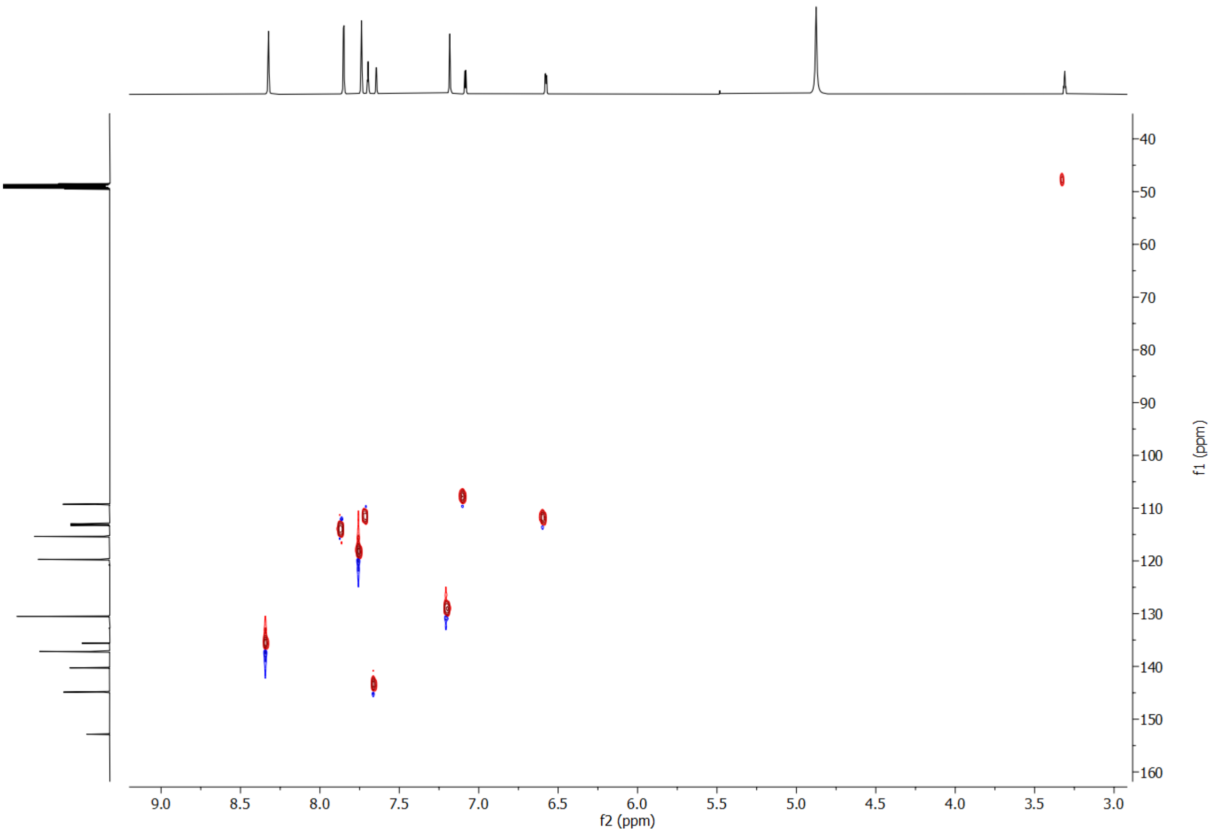

Figure S23: HSQC spectrum of 1,1'-(5-(furan-2-yl)-1,3-phenylene)bis(1*H*-imidazole) in CD_3OD (500 MHz, 126 MHz).

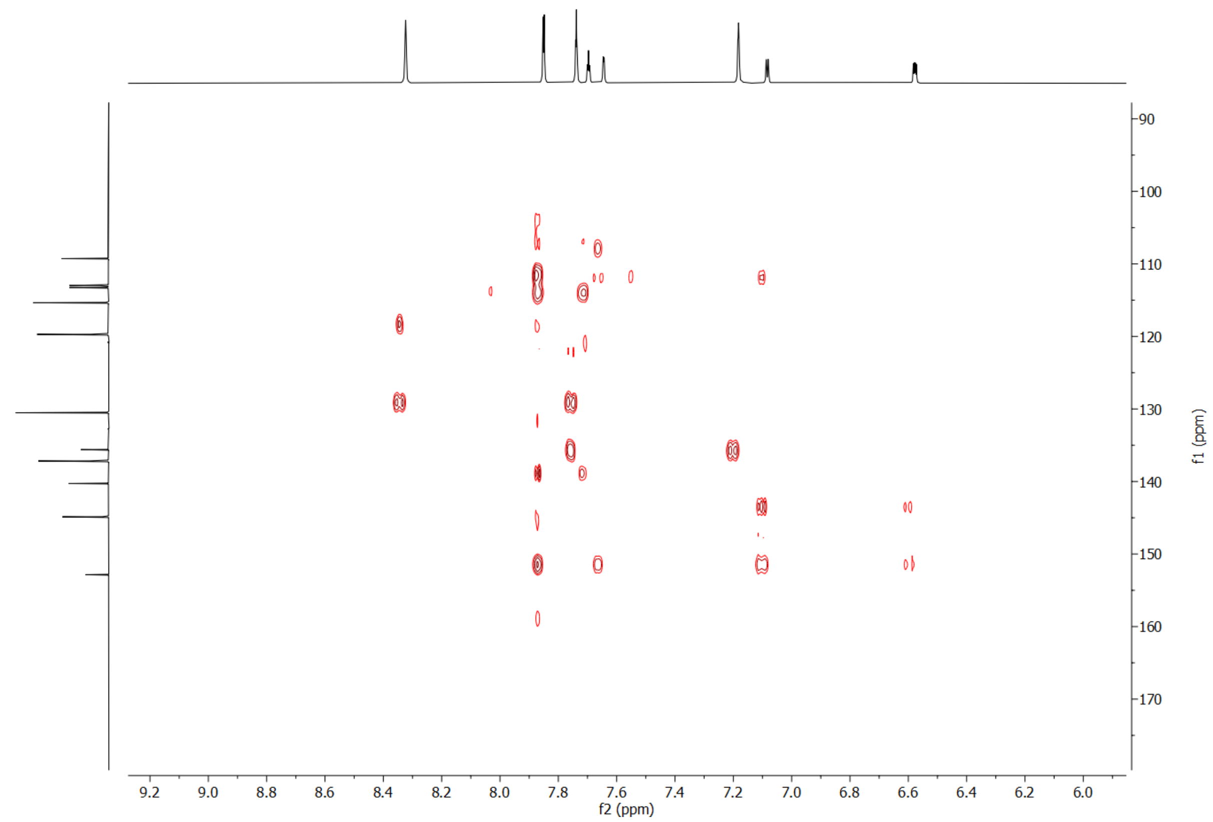

Figure S24: HMBC spectrum of 1,1'-(5-(furan-2-yl)-1,3-phenylene)bis(1*H*-imidazole)in CD_3OD (500 MHz, 126 MHz).

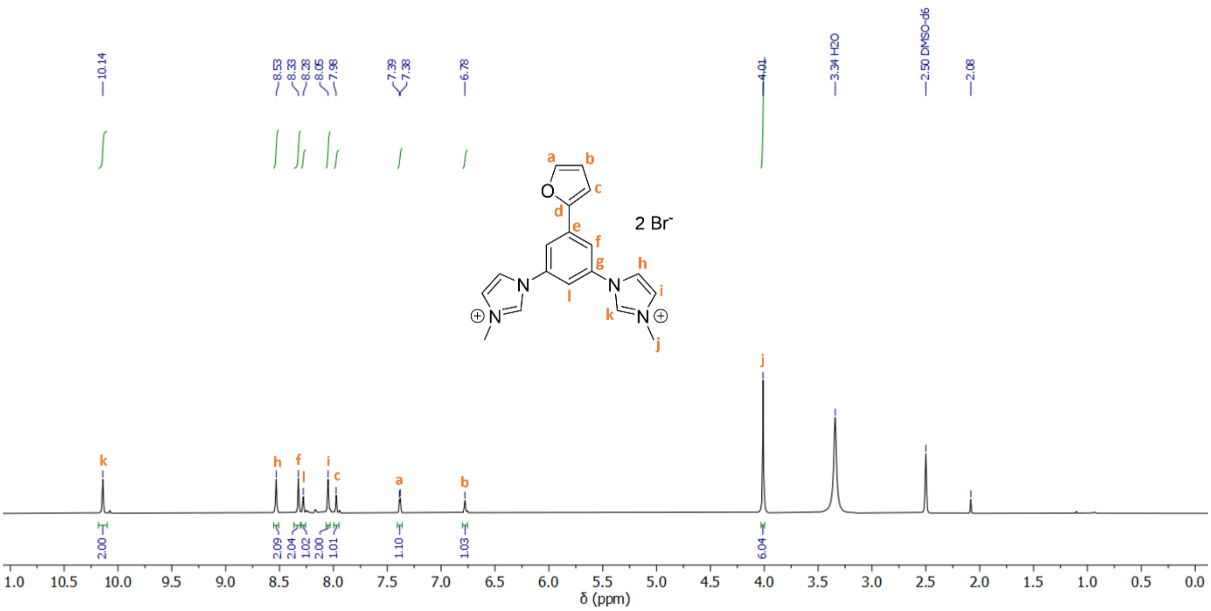

Figure S25: ^{1}H NMR spectrum of [HImPFur]Br_2 (15 mM) in DMSO-d_6.

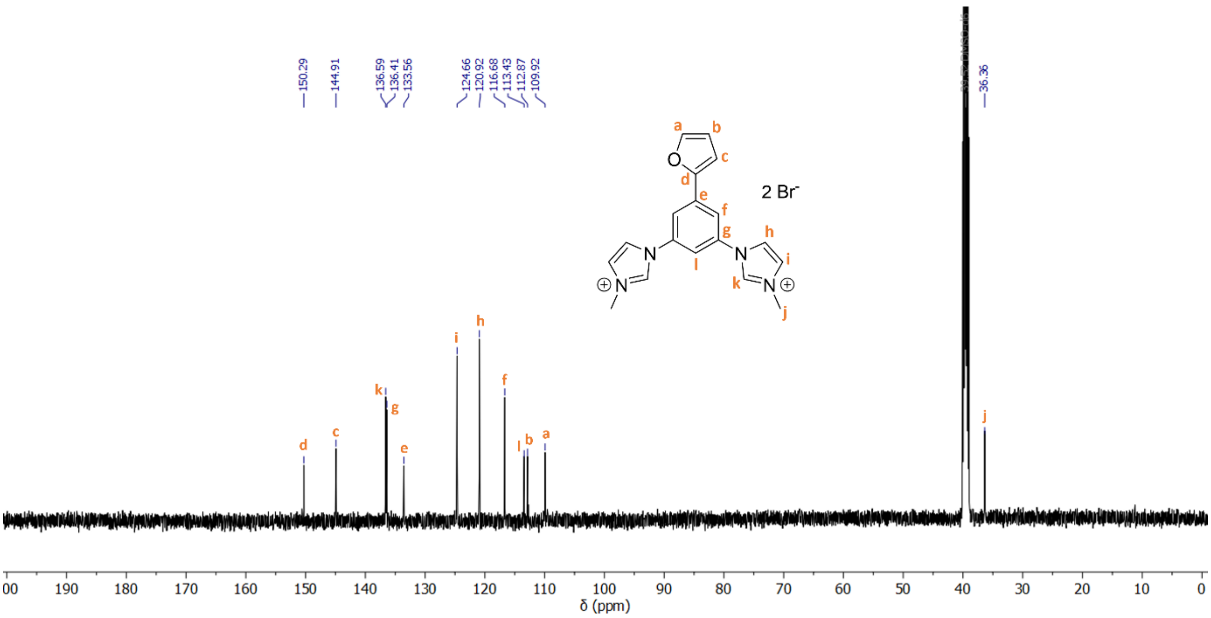

Figure S26: ^{13}C NMR spectrum of [HImPFur]Br_2 (15 mM) in DMSO-d_6.

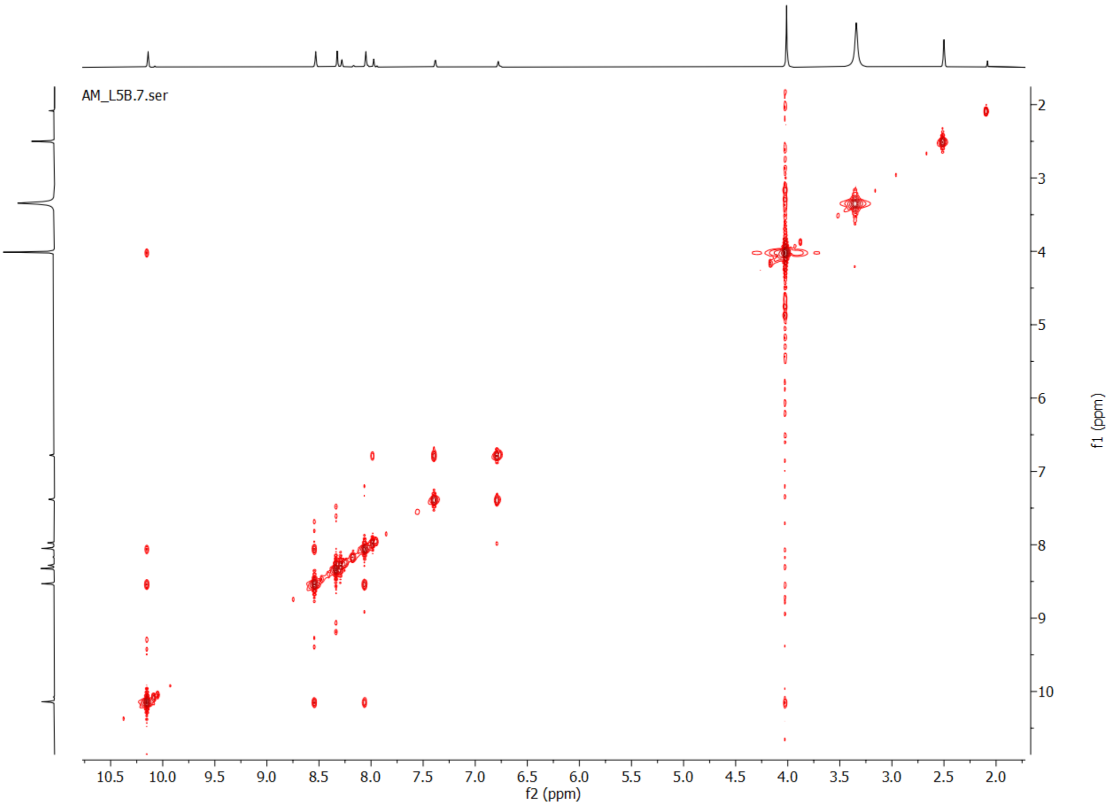

Figure S27: COSY spectrum of $[HImPFur]Br_2$ in DMSO-d_6 (500 MHz, 500 MHz).

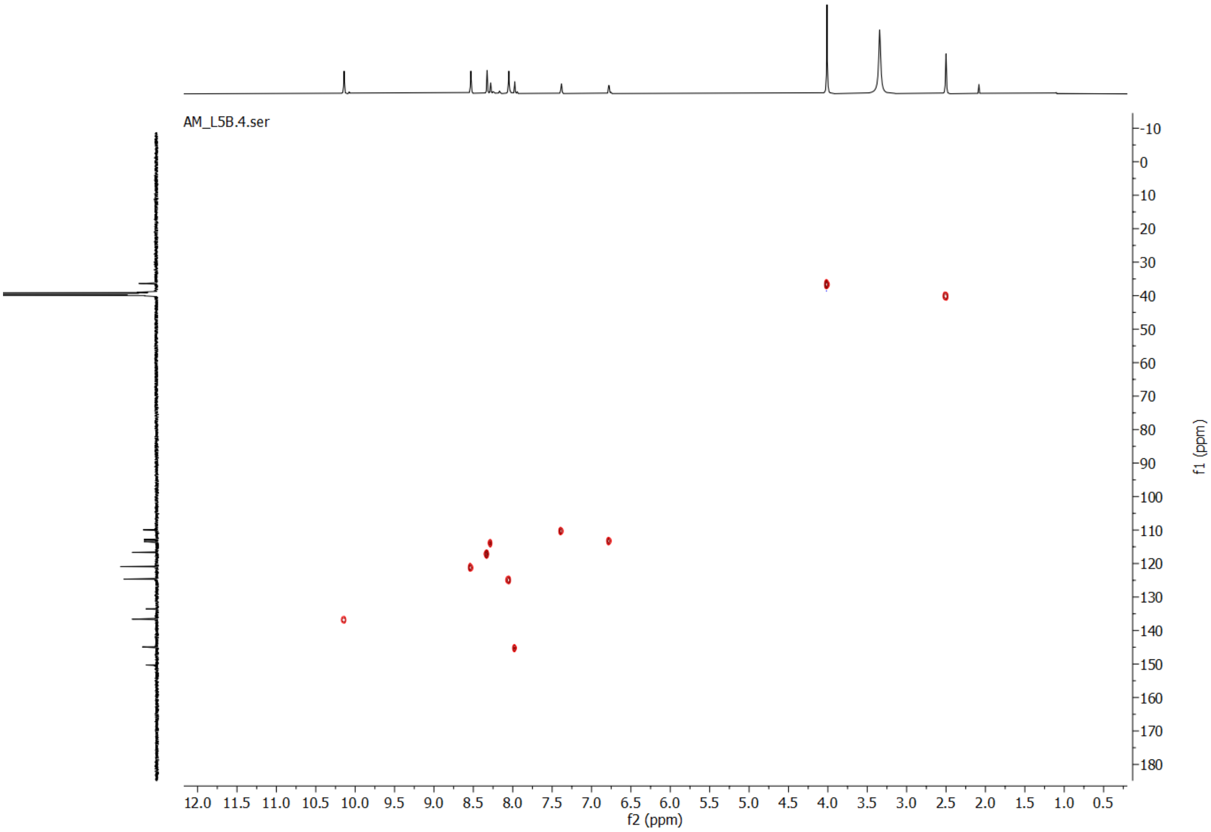

Figure S28: HSQC spectrum of $[HImPFur]Br_2$ in DMSO-d_6 (500 MHz, 126 MHz).

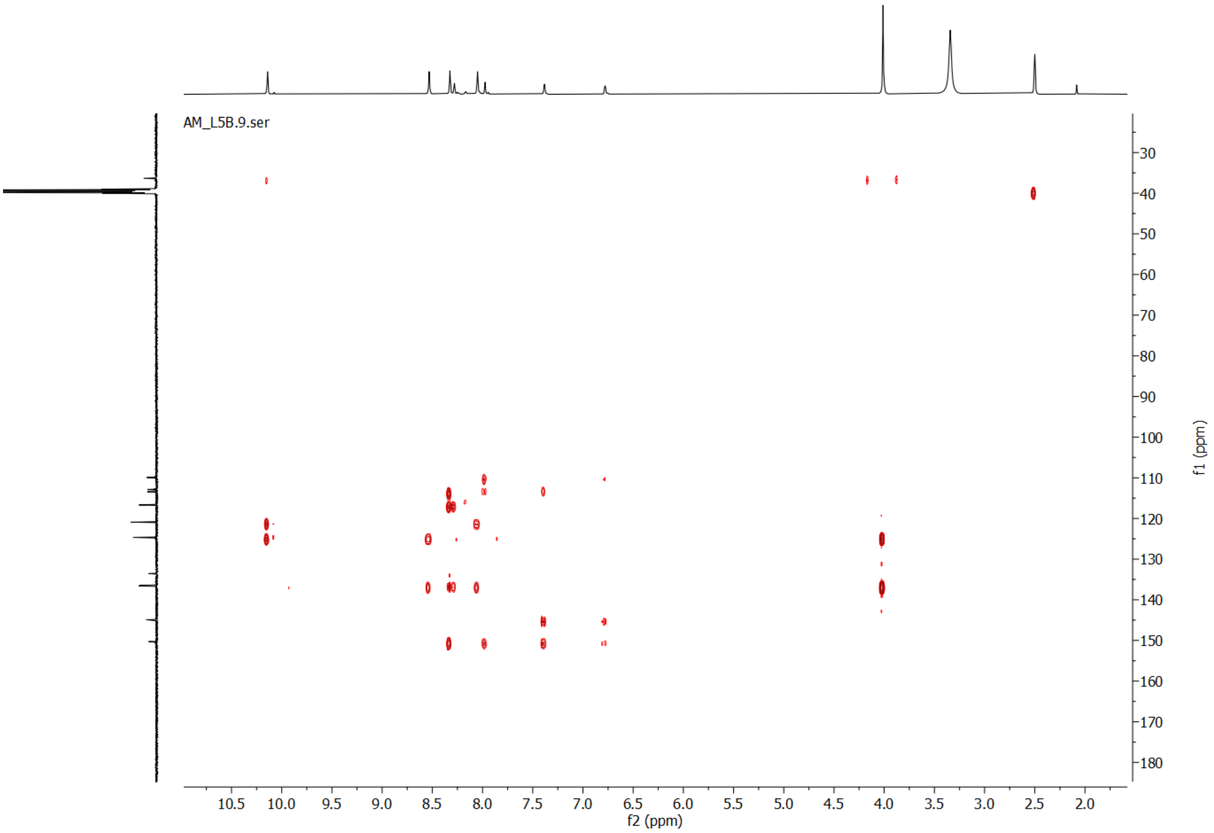

Figure S29: HMBC spectrum of $[HImPFur]Br_2$ in DMSO-d_6 (500 MHz, 126 MHz).

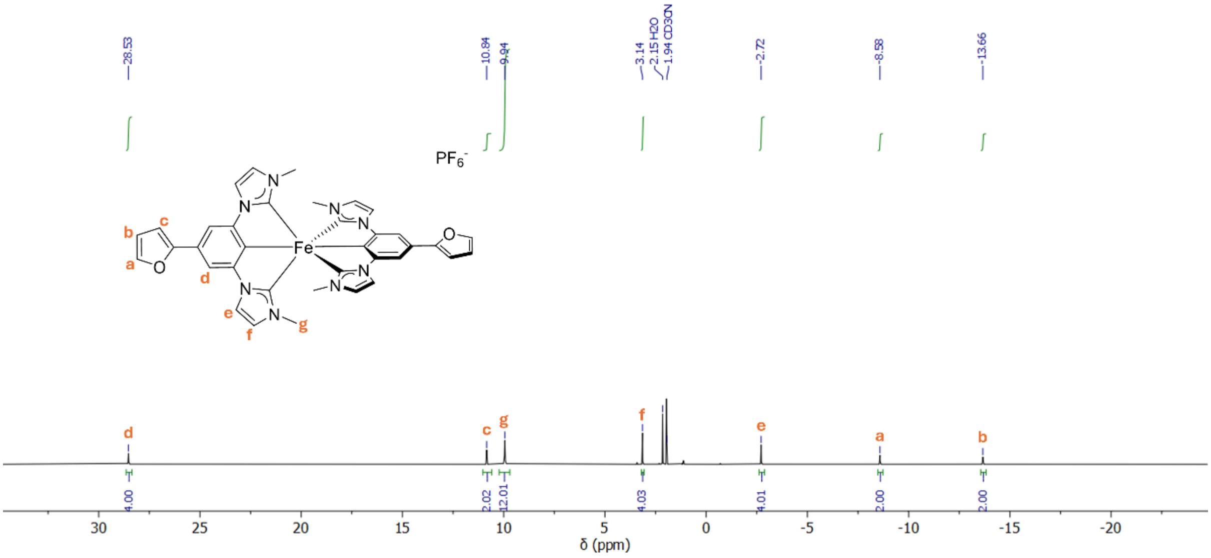

Figure S30: ^{1}H NMR spectrum of $[Fe(ImPFur)_2]PF_6$ (35 mM) in CD_3CN.

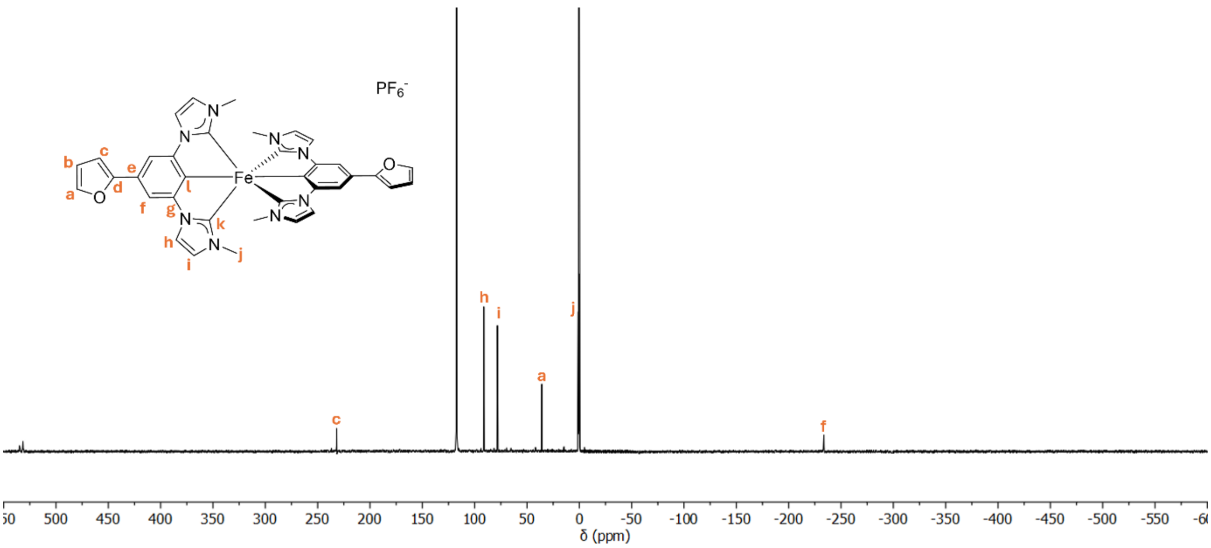

Figure S31: ^{13}C-NMR spectrum of $[Fe(ImPFur)_2]PF_6$ **(2)** (35 mM) in CD_3CN.

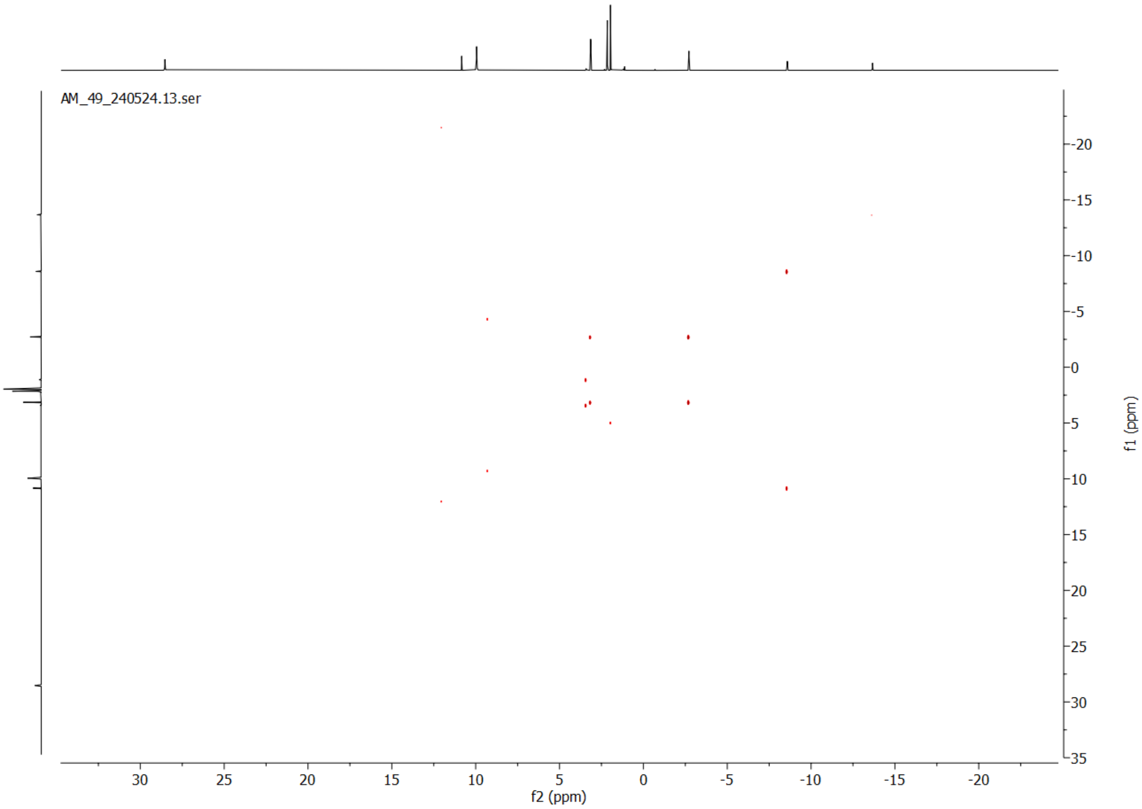

Figure S32: COSY spectrum of $[Fe(ImPFur)_2]PF_6$ in CD_3CN (600 MHz, 600 MHz).

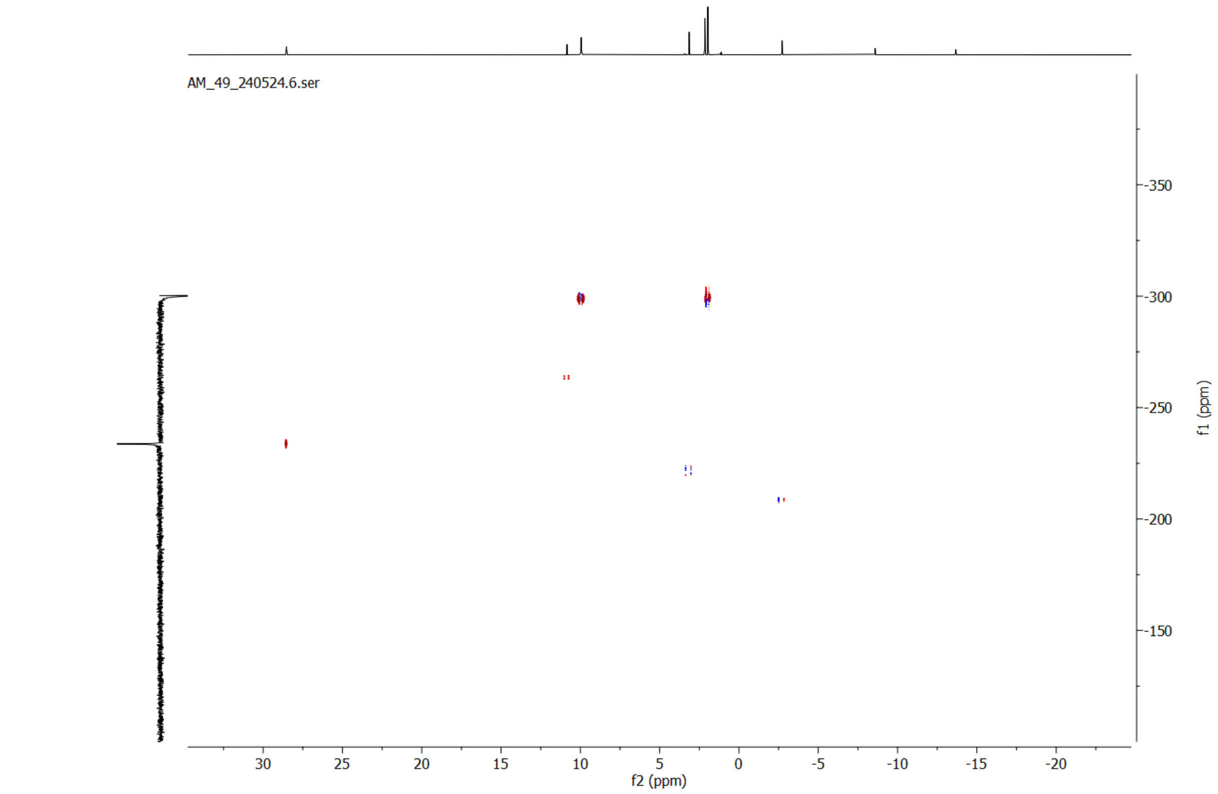

Figure S33: HSQC spectrum of $[Fe(ImPFur)_2]PF_6$ in CD_3CN (600 MHz, 151 MHz).

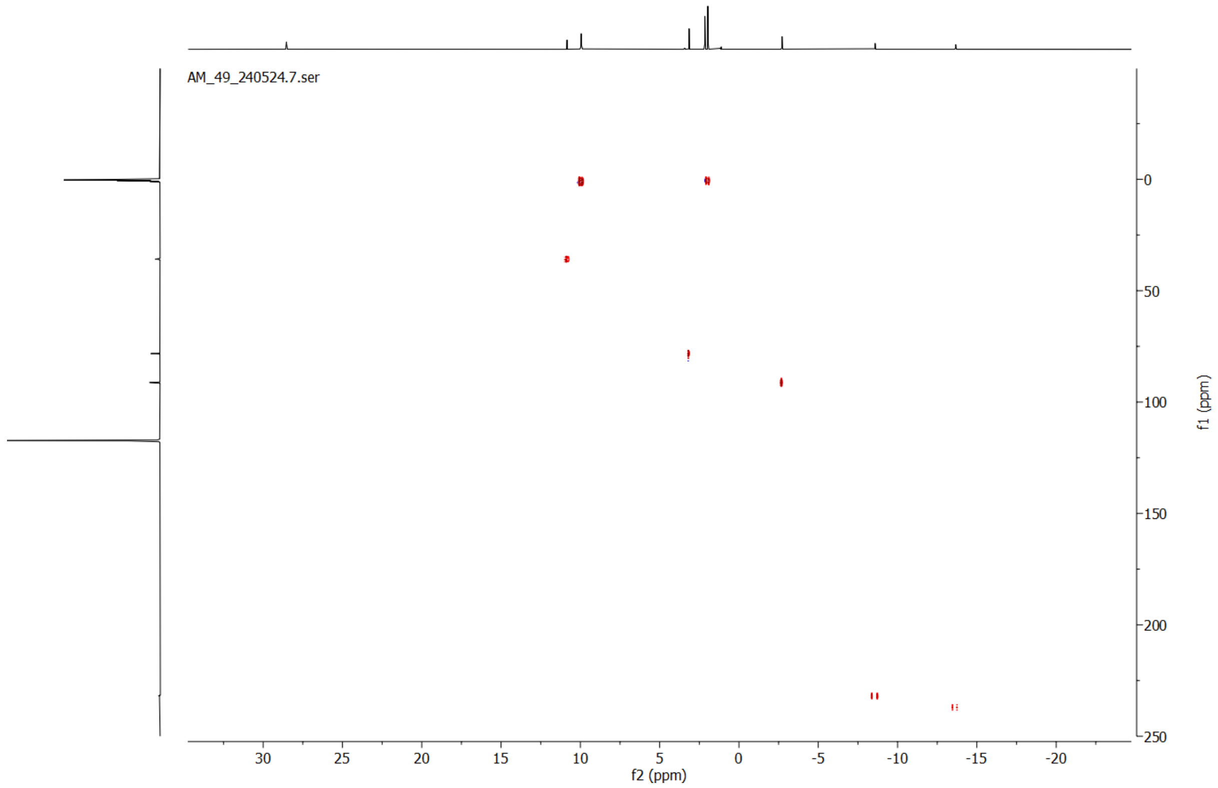

Figure S34: HSQC spectrum of $[Fe(ImPFur)_2]PF_6$ in CD_3CN (600 MHz, 151 MHz).

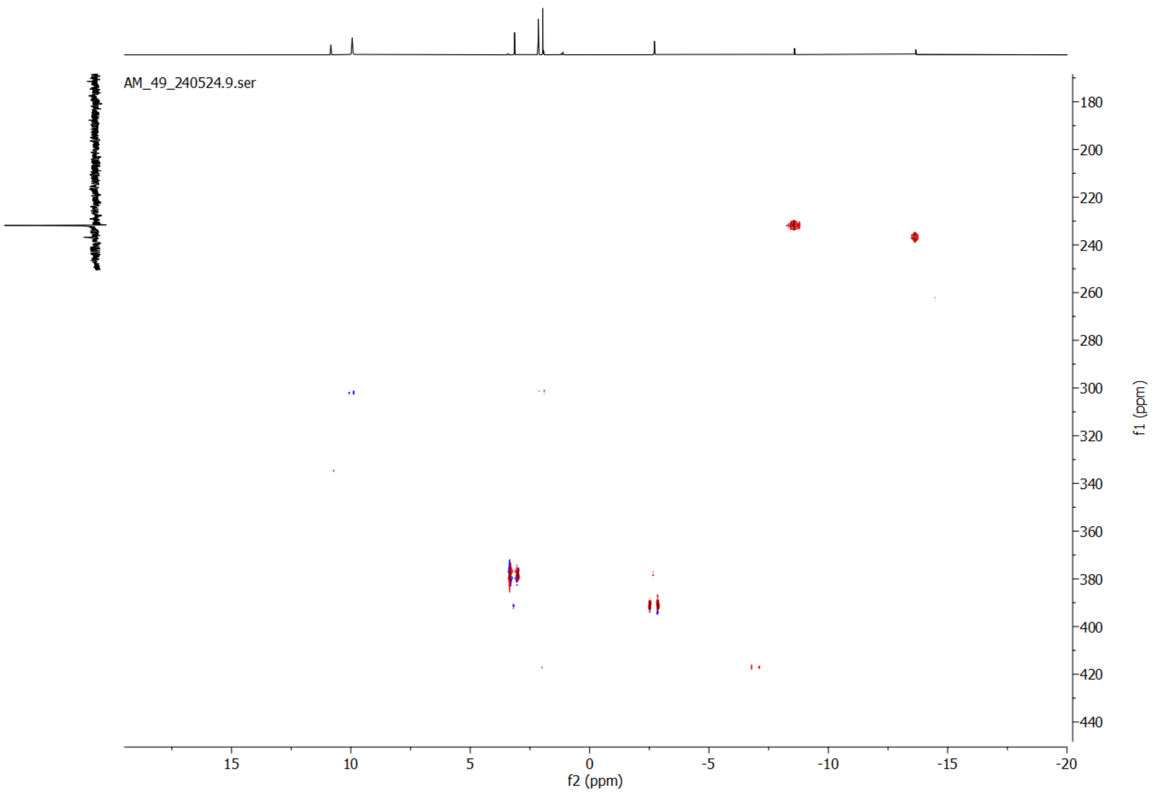

Figure S35: HSQC spectrum of $[Fe(ImPFur)_2]PF_6$ in CD_3CN (600 MHz, 151 Hz).

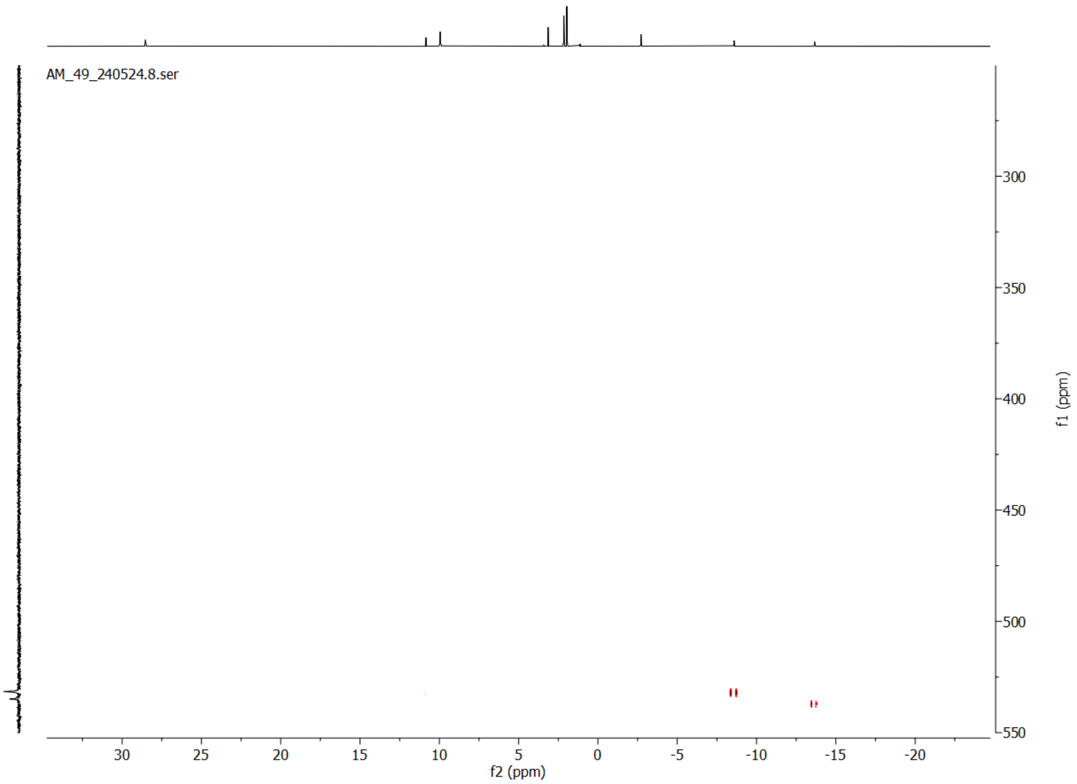

Figure S36: HSQC spectrum of $[Fe(ImPFur)_2]PF_6$ in CD_3CN (600 MHz, 151 Hz).

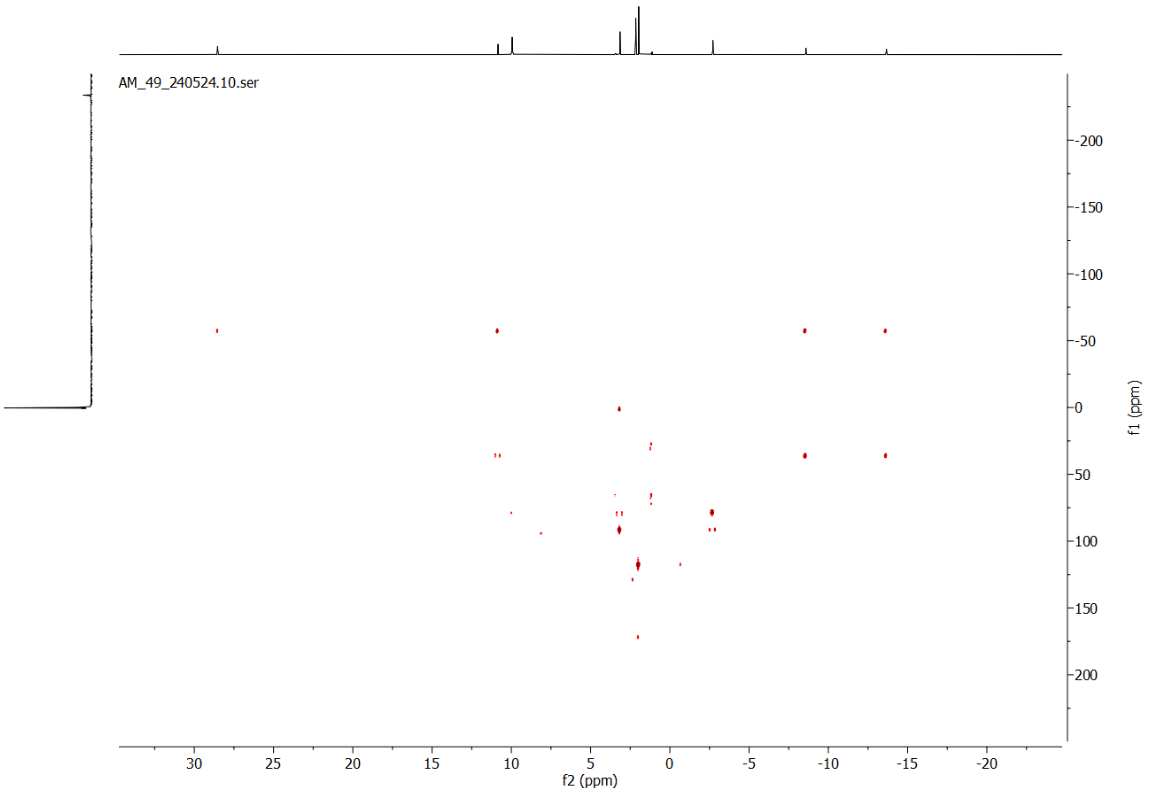

Figure S37: HMBC spectrum of $[Fe(ImPFur)_2]PF_6$ in CD_3CN (600 MHz, 151 Hz).

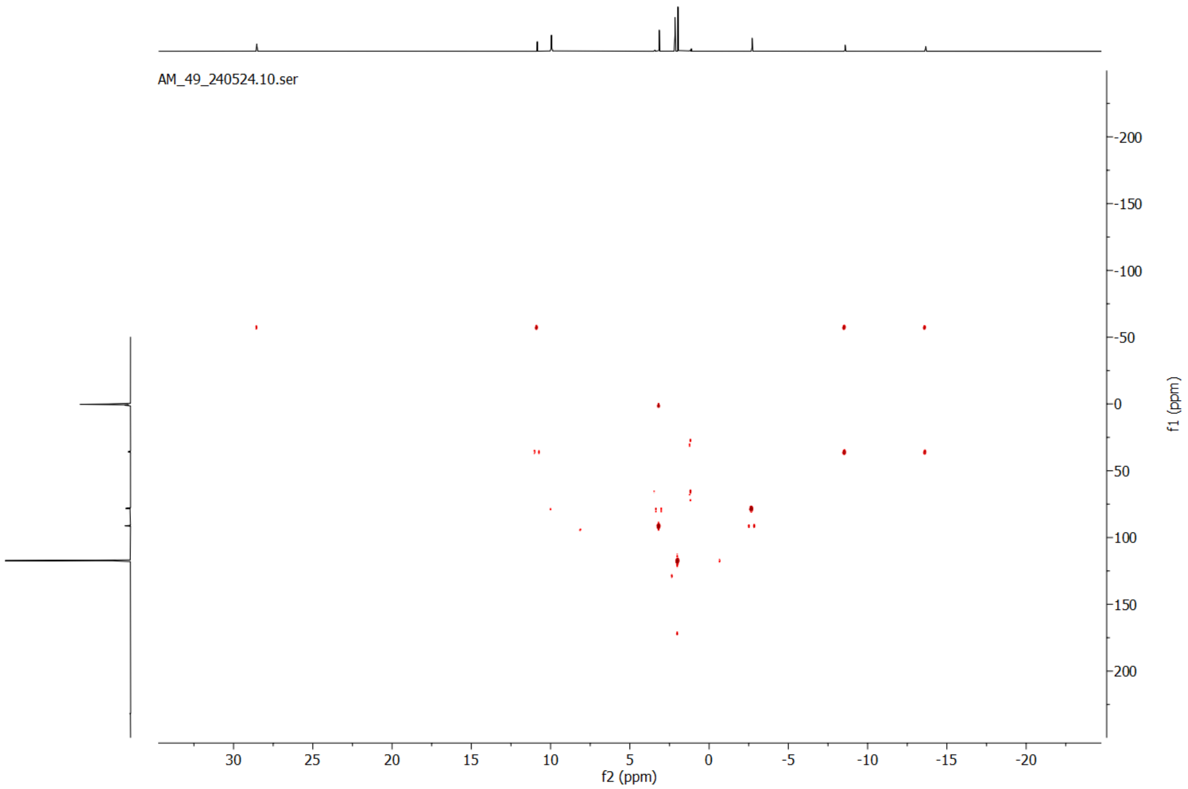

Figure S38: HMBC spectrum of $[Fe(ImPFur)_2]PF_6$ in CD_3CN (600 MHz, 151 MHz).

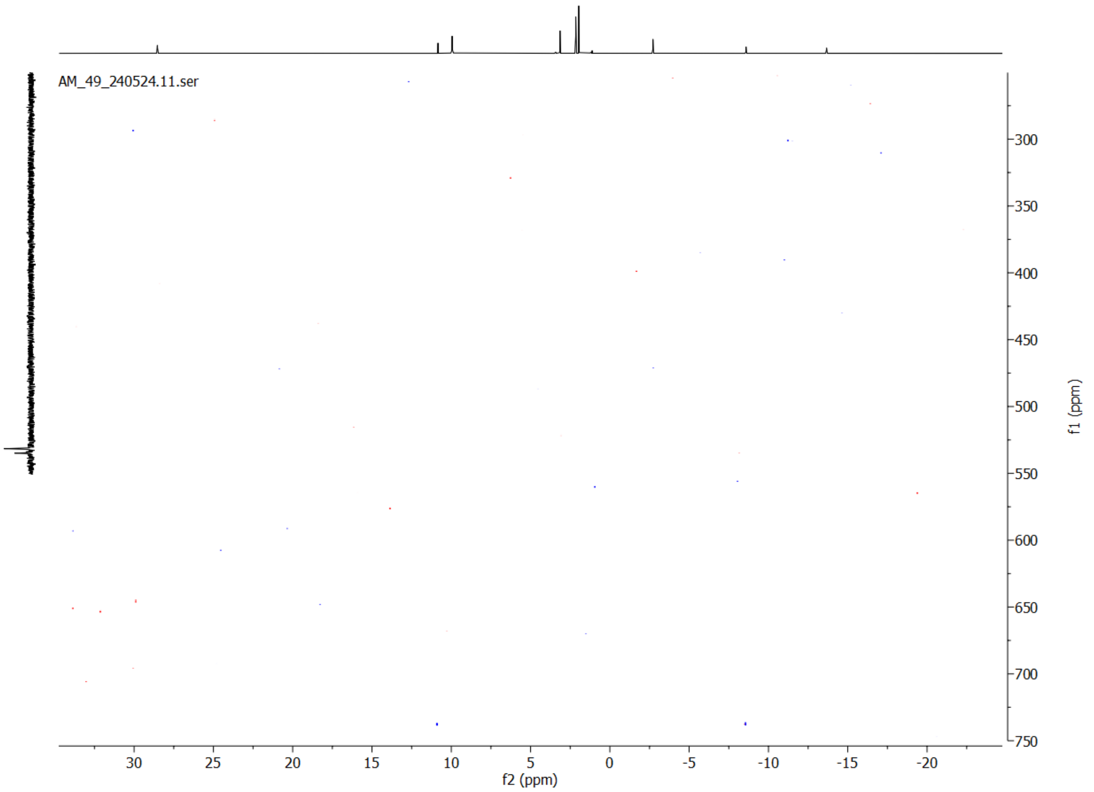

Figure S39: HMBC spectrum of $[Fe(ImPFur)_2]PF_6$ in CD_3CN (600 MHz, 151 MHz).

S4. HRMS

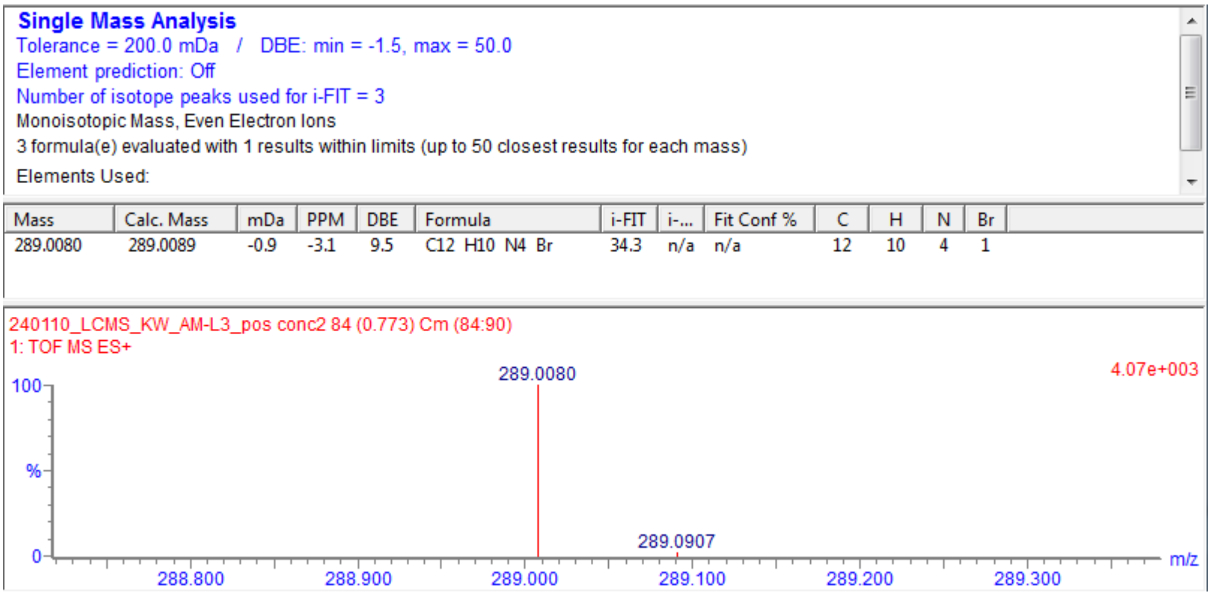

Figure S40: ESI-HRMS spectrum of 1,1'-(5-bromo-1,3-phenylene)bis(1*H*-imidazole).

Single Mass Analysis
Tolerance = 200.0 mDa / DBE: min = -1.5, max = 50.0
Element prediction: Off
Number of isotope peaks used for i-FIT = 3
Monoisotopic Mass, Even Electron Ions
7 formula(e) evaluated with 1 results within limits (up to 50 closest results for each mass)
Elements Used:

Mass	Calc. Mass	mDa	PPM	DBE	Formula	i-FIT	i-FIT N...	Fit C...	C	H	N	Br
476.8932	476.8925	0.7	1.5	7.5	C14 H16 N4 Br3	75.1	n/a	n/a	14	16	4	3

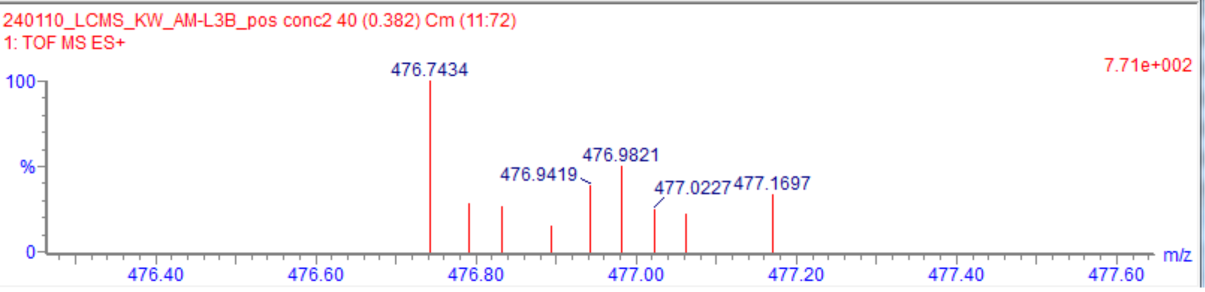

Figure S41: ESI-HRMS spectrum of pre-ligand [HImPBr]Br_2.

Single Mass Analysis
Tolerance = 100.0 mDa / DBE: min = -1.5, max = 100.0
Element prediction: Off
Number of isotope peaks used for i-FIT = 2
Monoisotopic Mass, Odd and Even Electron Ions
2 formula(e) evaluated with 1 results within limits (all results (up to 1000) for each mass)
Elements Used:

Mass	Calc. Mass	mDa	PPM	DBE	Formula	i-FIT	i-FIT No...	F...	C	H	N	Fe	Br
685.9850	685.9840	1.0	1.5	20.0	C28 H24 N8 Fe Br2	75.7	n/a	n...	28	24	8	1	2

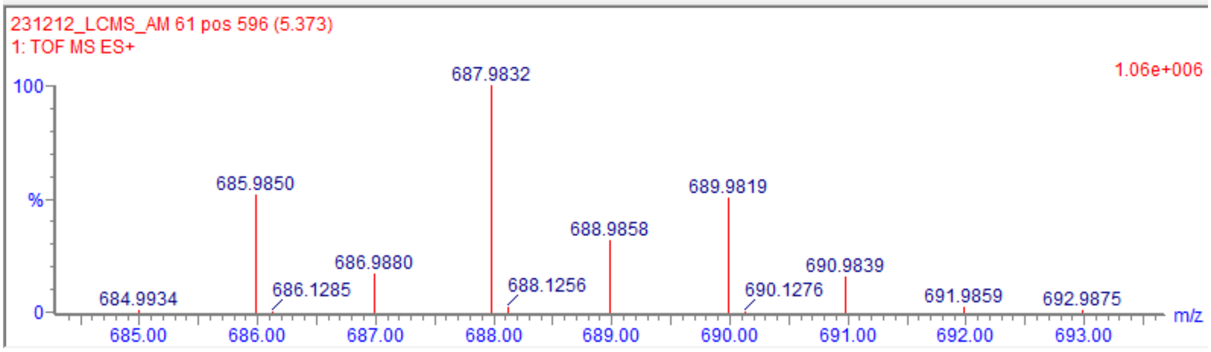

Figure S42: ESI-HRMS spectrum of the complex $[Fe(ImPBr)_2]PF_6$.

Single Mass Analysis
Tolerance = 10.0 mDa / DBE: min = -1.5, max = 100.0
Element prediction: Off
Number of isotope peaks used for i-FIT = 2
Monoisotopic Mass, Even Electron Ions
92 formula(e) evaluated with 2 results within limits (all results (up to 1000) for each mass)
Elements Used:
C: 0-60 H: 0-100 N: 0-5 O: 0-3

Mass	Calc. Mass	mDa	PPM	DBE	Formula	i-FIT	i-...	Fit Conf %	C	H	N	O
277.1089	277.1089	0.0	0.0	12.5	C16 H13 N4 O	54.4	0...	86.32	16	13	4	1
	277.1017	7.2	26.0	16.5	C22 H13	56.3	1...	13.68	22	13		

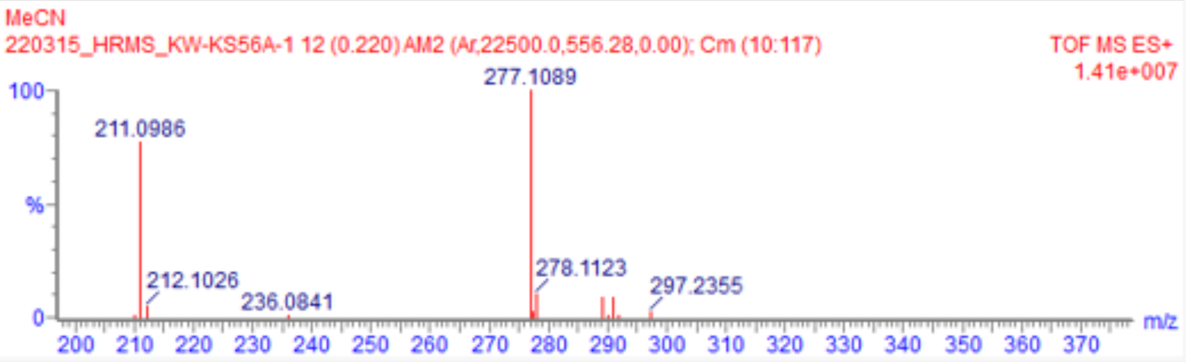

Figure S43: ESI-HRMS spectrum of 1,1'-(5-(furan-2-yl)-1,3-phenylene)bis(1*H*-imidazole).

Single Mass Analysis
Tolerance = 20.0 mDa / DBE: min = -1.5, max = 50.0
Element prediction: Off
Number of isotope peaks used for i-FIT = 3
Monoisotopic Mass, Even Electron Ions
10 formula(e) evaluated with 1 results within limits (up to 50 closest results for each mass)
Elements Used:

Mass	Calc. Mass	mDa	PPM	DBE	Formula	i-FIT	i-FIT...	Fit C...	C	H	N	O	Br
464.9920	464.9926	-0.6	-1.3	10.5	C18 H19 N4 O Br2	76.5	n/a	n/a	18	19	4	1	2

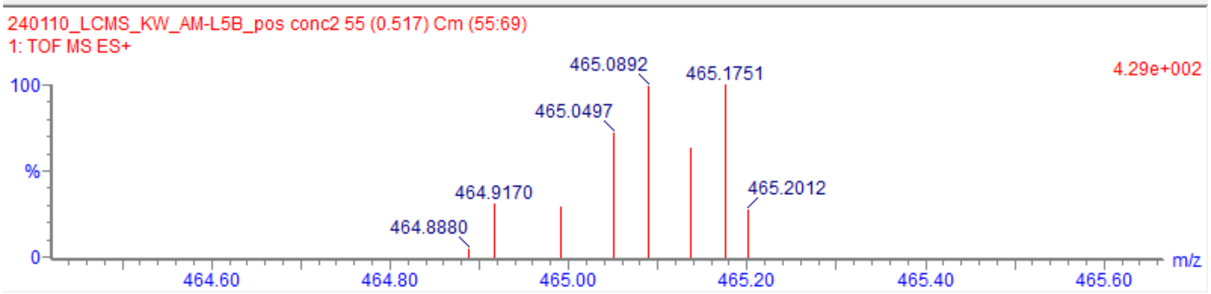

Figure S44: ESI-HRMS spectrum of the pre-ligand [HImPFur]Br$_2$.

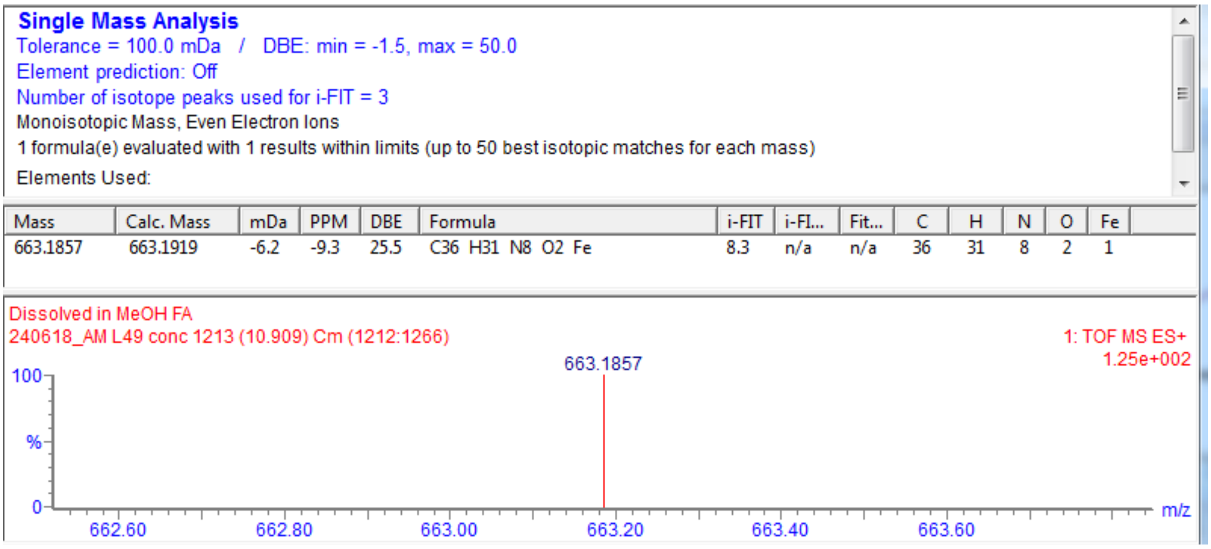

Figure S45: ESI-HRMS spectrum of the complex $[Fe(ImPFur)_2]PF_6$.

S5. Single Crystal X-ray Structure Determination

Suitable crystals for SC-XRD measurements were loaded on Agilent Xcalibur Sapphire3 diffractometer high-brilliance IμS radiation source using graphite-monochromatized Mo Kα radiation (λ = 0.71073 Å). All the structures were solved by direct methods using SHELXS-97 and refined by full-matrix least squares on F^2 using SHELXL-97.[15,16] The details pertaining to the data collection and refinement for ligands $[HImPBr](PF_6)_2$ and $[HImPFur](PF_6)_2$ are given in Table S1 while for complexes $[Fe(ImPBr)_2]PF_6$ and $[Fe(ImPFur)_2]PF_6$ are given in Table S2. Non-hydrogen atoms were refined with anisotropic displacement parameters. All the hydrogen atoms were included in idealized positions and their positions were refined isotropically by a riding model. A solvent mask was calculated for complex $[Fe(ImPBr)_2]PF_6$, and 292 electrons were found in a volume of 4733 Å^3 in 1 void per unit cell. This is consistent with the presence of 1.75 $[CH_3CN]$ per formula unit which account for 308 electrons per unit cell. The OLEX[2] solvent masking was used to treat diffuse scattering in complex $Fe(ImPFur)_2]PF_6$ which showed the presence of 18 electrons in a volume of 779 Å^3 in 1 void per unit cell. This is consistent with the presence of 0.4 $[CH_3CN]$ per formula unit which accounts for 18 electrons per unit cell.

Table S1. Crystal data and structure refinement for $[HImPBr](PF_6)_2$ and $[HImPFur](PF_6)_2$.

Identification code	$[HImPBr](PF_6)_2$	$[HImPFur](PF_6)_2$
Empirical formula	$C_{16}H_{18}BrF_{12}N_5P_2$	$C_{20}H_{21}F_{12}N_5OP_2$
Formula weight	650.20	637.36
Temperature/K	298.00	298
Crystal system	triclinic	triclinic
Space group	P-1	P-1
a/Å	10.6514(5)	8.4638(13)
b/Å	10.9773(6)	13.4692(17)
c/Å	12.6156(8)	13.7580(19)
α/°	87.413(5)	119.135(14)
β/°	75.200(6)	100.258(13)
γ/°	62.151(6)	95.586(12)
Volume/Å^3	1256.20(14)	1316.8(4)
Z	2	2
ρ_{calc}g/cm^3	1.719	1.607
μ/mm^{-1}	1.868	0.275

F(000)	644.0	644.0
Crystal size/mm^3	0.18 × 0.17 × 0.12	0.27 × 0.23 × 0.14
Radiation	Mo Kα (λ = 0.71073)	Mo Kα (λ = 0.71073)
2Θ range for data collection/°	6.706 to 58.844	6.728 to 50
Index ranges	-13 ≤ h ≤ 14, -14 ≤ k ≤ 15, -17 ≤ l ≤ 16	-10 ≤ h ≤ 10, -16 ≤ k ≤ 16, -16 ≤ l ≤ 16
Reflections collected	24467	23528
Independent reflections	6072 [R_{int} = 0.0291, R_{sigma} = 0.0352]	4623 [R_{int} = 0.1624, R_{sigma} = 0.0909]
Data/restraints/parameters	6072/340/316	4623/181/352
Goodness-of-fit on F^2	1.065	1.128
Final R indexes [I>=2σ (I)]	R_1 = 0.0762, wR_2 = 0.2223	R_1 = 0.1099, wR_2 = 0.2947
Final R indexes [all data]	R_1 = 0.1045, wR_2 = 0.2485	R_1 = 0.1432, wR_2 = 0.3362
Largest diff. peak/hole / e Å^{-3}	1.20/-0.72	0.73/-0.55
CCDC	2455513	2455515

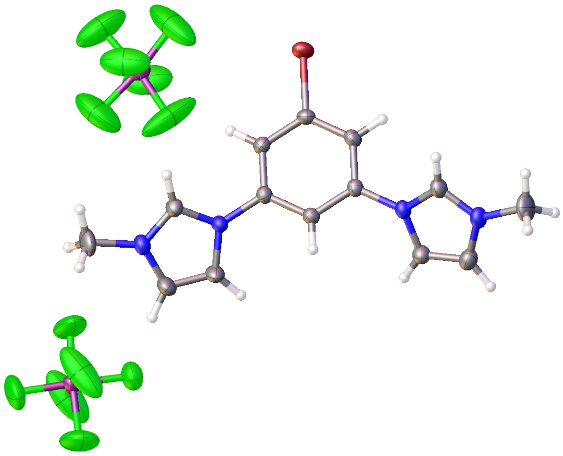

Figure S46: Molecular structure of the pre-ligand [HImPBr](PF_6)$_2$. Carbon atoms are displayed by gray ellipsoids, nitrogen by blue, oxygen by red, hydrogen by white, bromine by dark red, phosphorus by purple and fluorine by green.

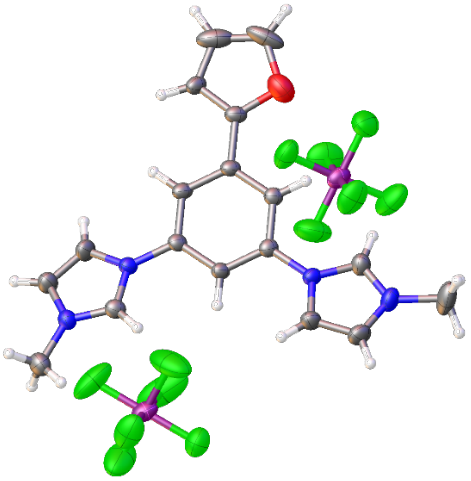

Figure S47: Molecular structure of the pre-ligand [HImPFur](PF_6)$_2$. Carbon atoms are displayed by gray ellipsoids, nitrogen by blue, oxygen by red, hydrogen by white, phosphorus by purple and fluorine by green.

Table S2. Crystal data and structure refinement for complexes [Fe(ImPBr)$_2$]PF_6 and Fe(ImPFur)$_2$]PF_6.

Identification code	[Fe(ImPBr)$_2$]PF_6	[Fe(ImPFur)$_2$]PF_6
Empirical formula	$C_{28}H_{24}Br_2F_6FeN_8P$	$C_{36}H_{30}F_6FeN_8O_2P$

Formula weight	3332.76	807.50
Temperature/K	150	293
Crystal system	tetragonal	triclinic
Space group	P4/mnc	P-1
a/Å	21.0423(5)	11.5584(8)
b/Å	21.0423(5)	11.9479(8)
c/Å	20.5164(8)	17.2521(9)
α/°	90	75.974(5)
β/°	90	70.980(6)
γ/°	90	63.021(7)
Volume/Å^3	9084.2(6)	1994.6(3)
Z	8	2
ρ_{calc}g/cm^3	1.218	1.344
μ/mm^{-1}	2.175	0.488
F(000)	3304.0	826.0
Crystal size/mm^3	0.3 × 0.24 × 0.16	0.32 × 0.28 × 0.2
Radiation	MoKα (λ = 0.71073)	MoKα (λ = 0.71073)
2Θ range for data collection/°	6.766 to 50	6.668 to 59.068
Index ranges	-25 ≤ h ≤ 24, -25 ≤ k ≤ 25, -24 ≤ l ≤ 24	-15 ≤ h ≤ 15, -15 ≤ k ≤ 16, -23 ≤ l ≤ 21
Reflections collected	130816	41164
Independent reflections	4119 [R_{int} = 0.2155, R_{sigma} = 0.0656]	9762 [R_{int} = 0.0820, R_{sigma} = 0.0955]
Data/restraints/parameters	4119/45/236	9762/105/524
Goodness-of-fit on F^2	1.044	0.973
Final R indexes [I>=2σ (I)]	R_1 = 0.0675, wR_2 = 0.1792	R_1 = 0.0802, wR_2 = 0.2220
Final R indexes [all data]	R_1 = 0.1006, wR_2 = 0.1952	R_1 = 0.1417, wR_2 = 0.2671
Largest diff. peak/hole / e Å^{-3}	0.93/-0.53	0.80/-0.39
CCDC	2455516	2455514

Table S3. Selected Bond Lengths for complex Fe(ImPBr)$_2$]PF$_6$.

**Atom**	**Length/Å**
Fe1-C4	1.944(5)
Fe1-C9	2.011(5)
Fe1-C13	1.980(5)

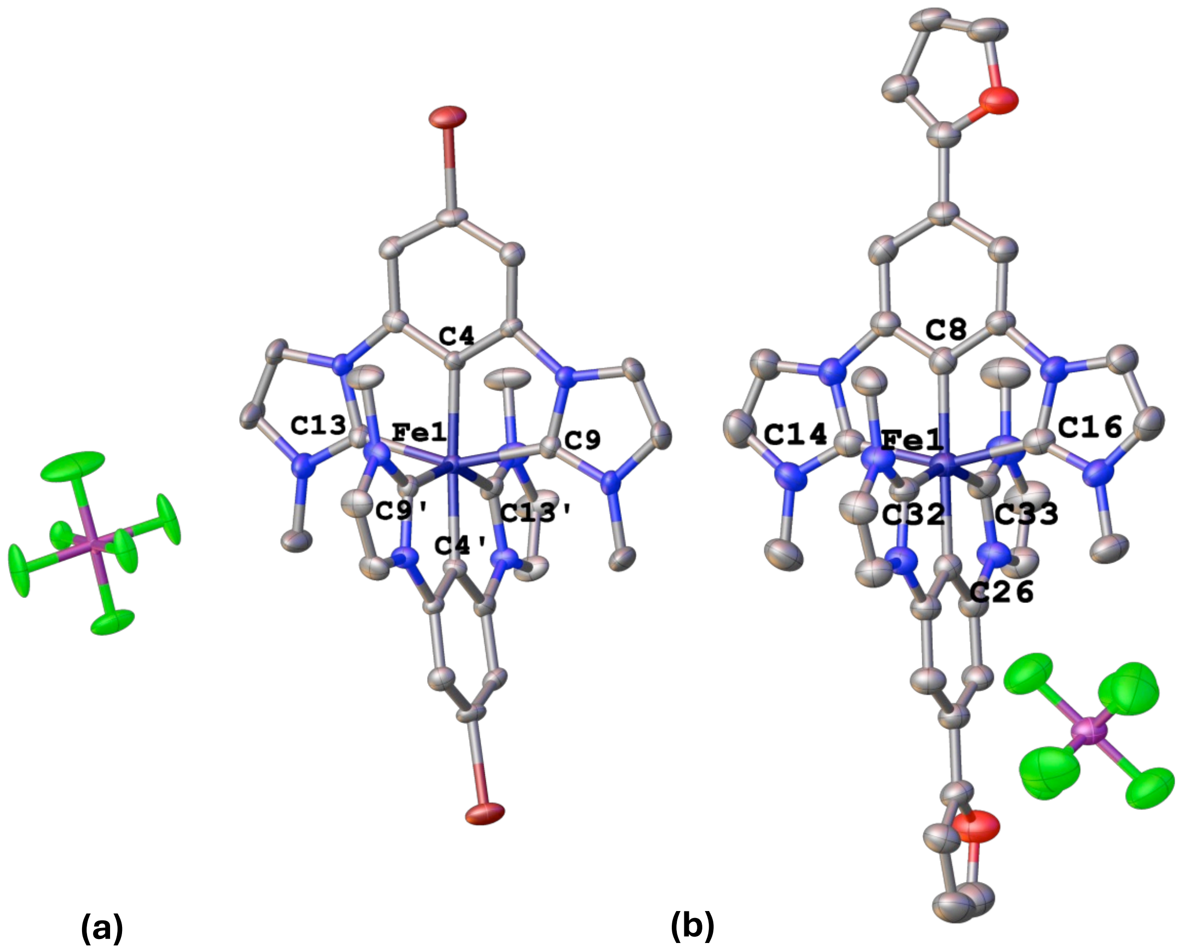

Figure S48: Molecular structure of the complex $[Fe(ImPBr)_2](PF_6)$ and $[Fe(ImPFur)_2](PF_6)$. Carbon atoms are displayed by gray ellipsoids, nitrogen by blue, oxygen by red, bromine by dark red, iron by dark blue, phosphorus by purple and fluorine by green. Hydrogens atoms and solvent of crystallization were omitted for clarity.

Table S4. Selected Bond Angles for complex $Fe(ImPBr)_2]PF_6$.

Atom	Angle/°	Atom	Angle/°
C4- Fe1- C9	77.3(2)	C4- Fe1- C13'	107.4(2)
C4- Fe1- C9'	97.8(2)	C4- Fe1- C13	77.8(2)
C4- Fe1- C4'	172.9(3)	C9- Fe1- C9'	94.0(3)
C4'- Fe1- C9	97.8(2)	C13- Fe1- C9'	92.5(2)
C9- Fe1- C13	154.9(2)	C13- Fe1- C13'	91.7(3)

Table S5. Selected Bond Lengths for complex $Fe(ImPFur)_2]PF_6$.

Atom	Length/Å	Atom	Length/Å
Fe1-C8	1.945(4)	Fe1-C26	1.938(4)
Fe1-C14	1.988(4)	Fe1-C32	1.984(4)
Fe1-C16	1.991(4)	Fe1-C33	1.997(4)

Table S6. Selected Bond Angles for complex Fe(ImPFur)$_2$]PF$_6$.

Atom	Angle/°	Atom	Angle/°
C8- Fe1- C14	77.75(17)	C26- Fe1- C14	103.20(16)
C8- Fe1- C16	77.77(16)	C26- Fe1- C16	101.28(16)
C8- Fe1- C32	103.42(16)	C26- Fe1- C32	77.22(16)
C8- Fe1- C33	101.70(17)	C26- Fe1- C33	77.68(16)
C14- Fe1- C16	155.52(18)	C32- Fe1- C14	93.19(16)
C14- Fe1- C33	90.97(16)	C32- Fe1- C16	92.43(16)
C16- Fe1- C33	93.98(17)	C32- Fe1- C33	154.85(17)
C26- Fe1- C8	178.85(16)		

S6. Electron paramagnetic resonance measurements

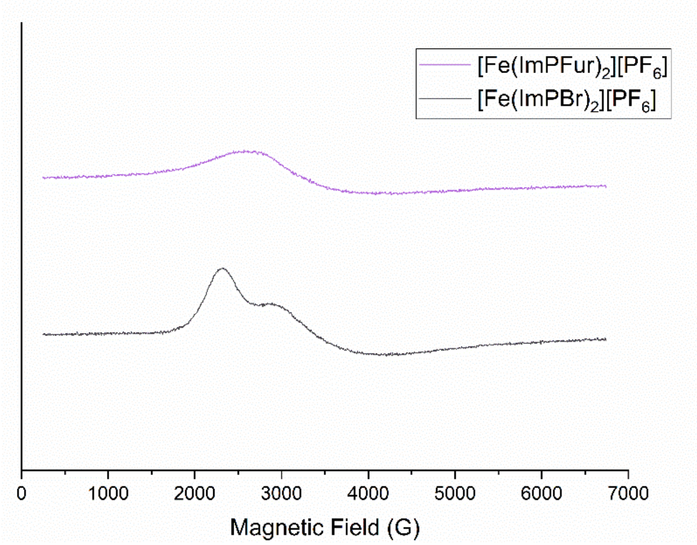

Figure S49: EPR Spectrum of complexes [Fe(ImPBr)$_2$]PF$_6$ and [Fe(ImPFur)$_2$]PF$_6$ showing the paramagnetic nature of the complexes.

S7. Magnetic susceptibility and magnetization measurements

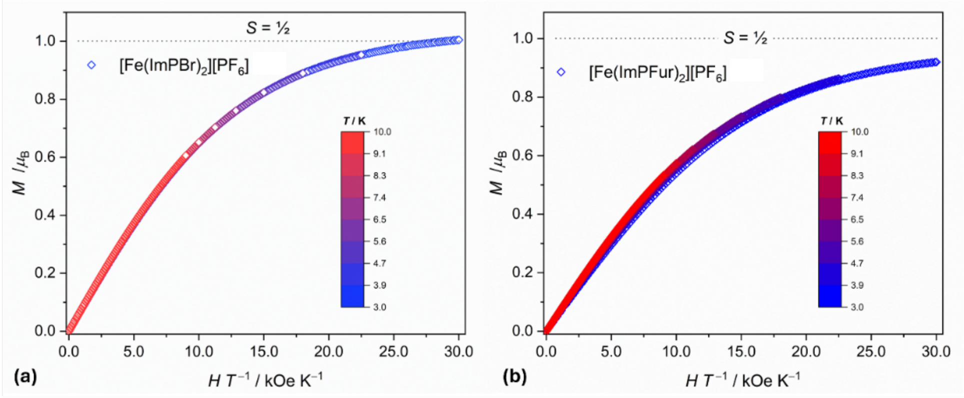

Figure S50: The plots of the magnetization measurements for complexes $[Fe(ImPBr)_2]PF_6$ and $[Fe(ImPFur)_2]PF_6$. recorded at temperatures 3-10 K.

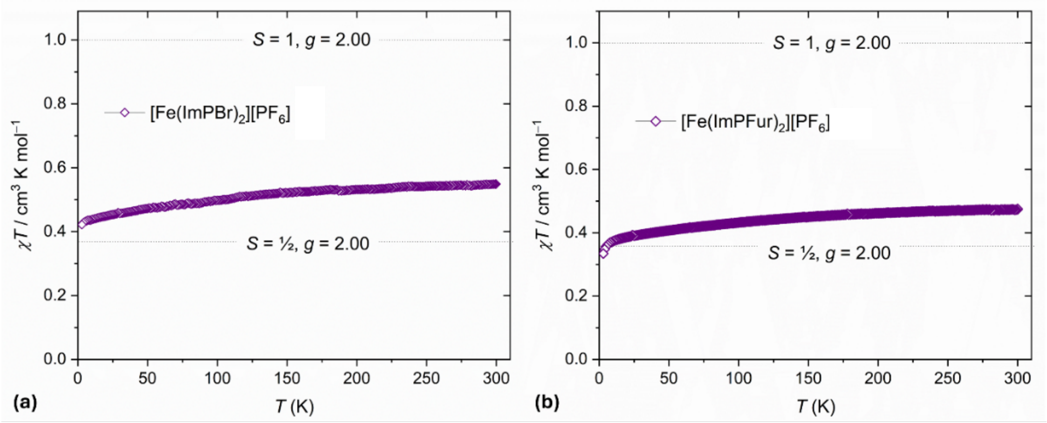

Figure S51: The plot of magnetic susceptibility vs temperature for complexes $Fe(ImPBr)_2]PF_6$ and $Fe(ImPFur)_2]PF_6$.

S8. Mössbauer Measurements

The ^{57}Fe Mössbauer spectra of all the samples at 295 K and 85 K reveal a quadrupole split doublet structure (Figure 1). The fitting results are presented in Table 1.

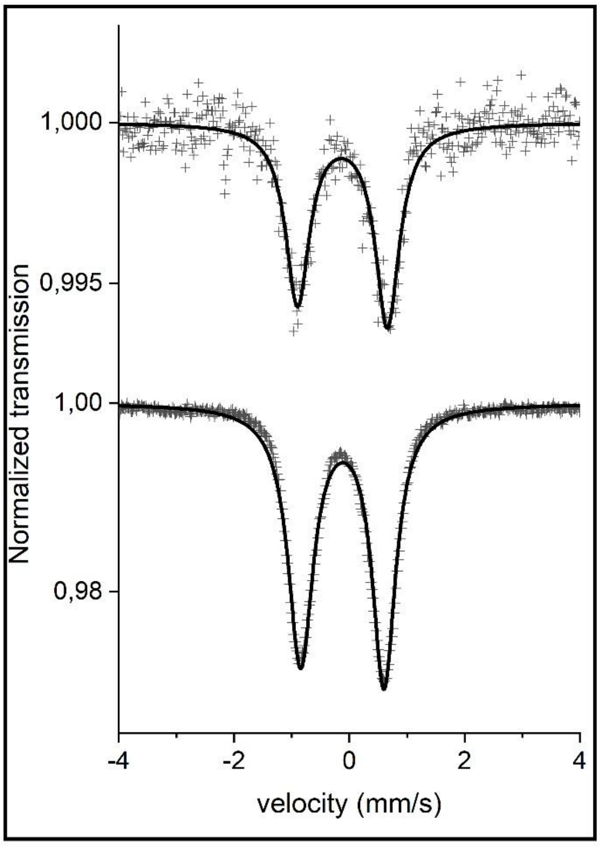

Figure S52: Mössbauer spectra of complex $[Fe(ImPBr)_2]PF_6$ (above) and complex $[Fe(ImPFur)_2]PF_6$ (below) recorded at 85 K. The transmission is low for compound $Fe(ImPBr)_2]PF_6$ due to the high absorption coefficient for Br of the 14.4 keV Mössbauer radiation.

Table S7. Results of the fitting procedure of the 85 K Mössbauer spectra. CS is the center shift relative natural a-Fe held at 295K, |QS| is the magnitude of the electric quadrupole splitting, G is the FWHM Lorentzian line width. Errors in these parameters are ±0.005 mm/s. LS stands for low spin.

Sample	CS (mm/s)	|QS|(mm/s)	W (mm/s)	Fe valence
$[Fe(ImPBr)_2]PF_6$ (**1)**	-0.119	1.559	0.508	LS Fe(III)
$[Fe(ImPFur)_2]PF_6$ (**2)**	-0.122	1.446	0.493	LS Fe(III)

The found center shifts CS and magnitude of electric quadrupole splittings |QS| for Fe ions in complexes **1** and **2** fall close to the low spin Fe(III) values found for other samples measured in this type of Fe-complexes[17–20].

The center shifts found at 295 K are ~0.07 mm/s lower than the values at 85 K, partly due to the second order Doppler shift, between 295 K and 85 K, which is ~ 0.12 mm/s assuming a Debye temperature q_D = 300 K.

The largest contribution to the magnitude of the electric quadrupole splitting QS in low spin Fe(III) complexes emanates from the unpaired Fe 3d valence electrons[21]. Normally the ligand contribution is smaller than the valence electron contribution and furthermore the two contributions have different signs[22]. The present type of Mössbauer spectroscopic powder studies cannot however measure the sign of the electric quadrupole splitting.

The near lattice surroundings for the present Fe(III) complexes are Fe surrounded by 6 C atoms in a distorted octahedral configuration (Fig. S53). For a non-distorted octahedral 6C configuration the ligand contribution to QS would be zero. In the present cases the octahedral surroundings are not perfect hence why small ligand contributions to QS are expected.

The magnitude of the electric quadrupole splittings at 85 K are ~ 0.36 mm/s larger than the corresponding values at 295 K. These changes likely originate from the contraction of the lattice at lower temperatures.

The two lines in the doublets (Fig. S52) have different linewidths (W_-/W_+ ~ 1.09) indicating magnetic relaxation effects, typical for low spin Fe(III) complexes.

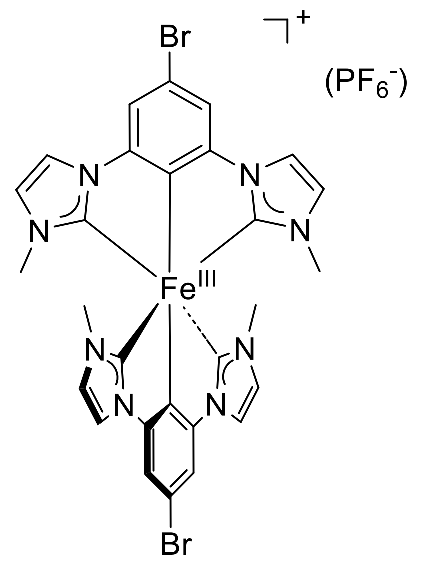

[Fe(ImPBr)$_2$]PF$_6$

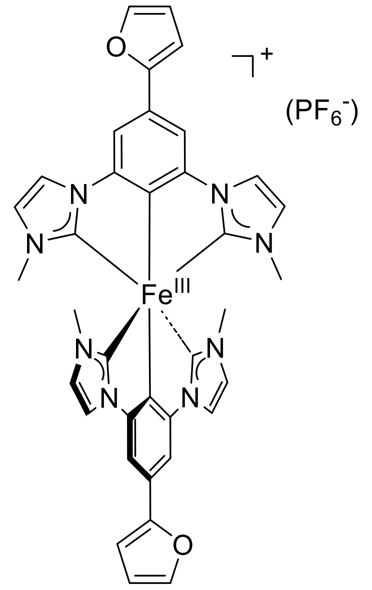

[Fe(ImPFur)$_2$]PF$_6$

Figure S53: Molecular structures of [Fe(ImPBr)$_2$]PF$_6$ and [Fe(ImPFur)$_2$]PF$_6$ showing the near octahedral atomic surrounding for Fe.

S9. Fs-TAS and Global Analysis

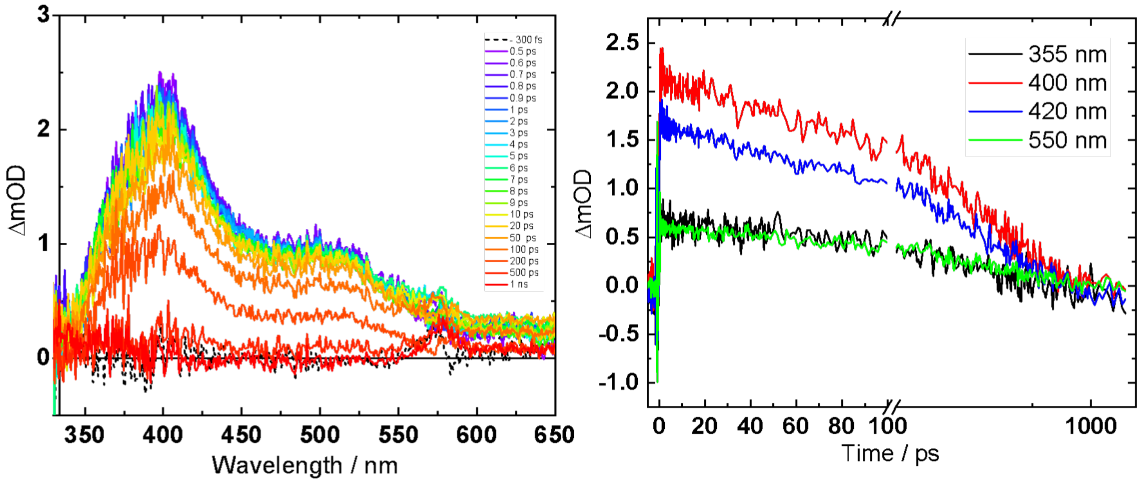

Figure S54. Fs-TAS spectra at selected time delays and kinetics at selected wavelengths of complex $[Fe^{III}(ImPBr)_2]^+$ (585 nm, E = 0.35 ± 0.04 mW).

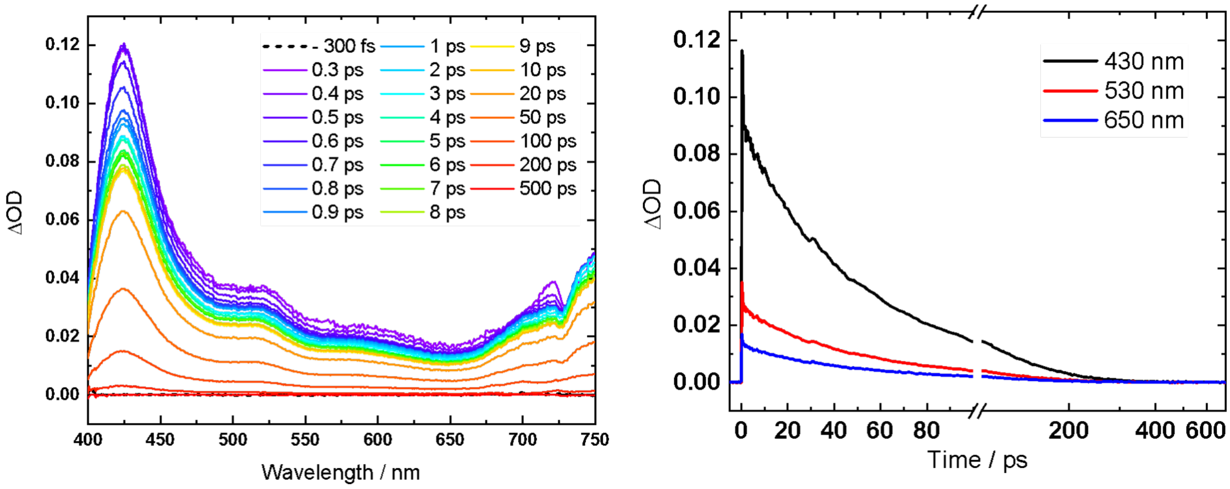

Figure S55. Fs-TAS spectra at selected time delays and kinetics at selected wavelengths of complex $[Fe^{III}(ImPFur)_2]^+$ (700 nm, E = 2.3 ± 0.3 mW).

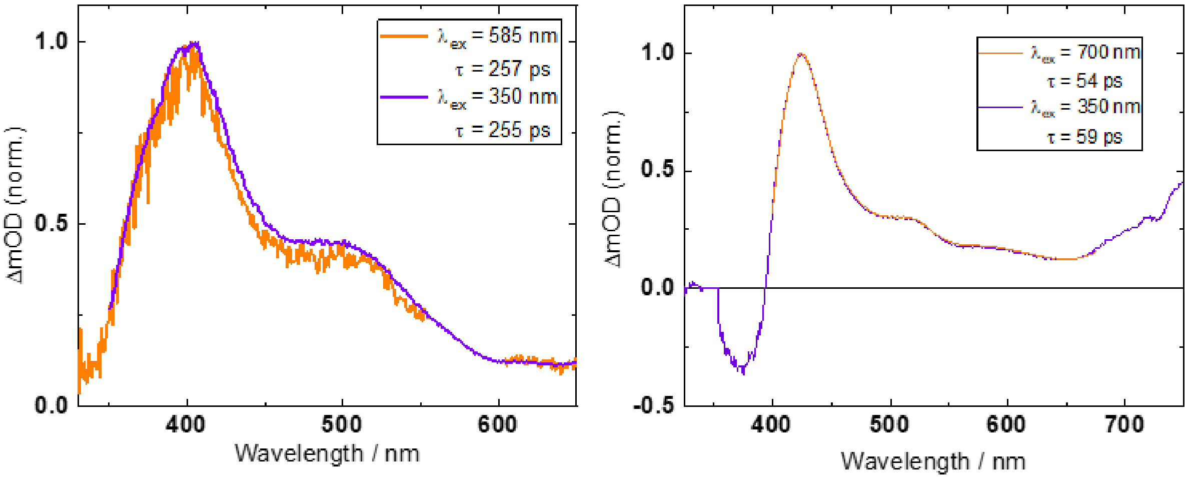

Figure S56. Decay associated spectra obtained from the global analysis of the fs-TAS data. $[Fe(ImPBr)_2]^+$ (left)and $[Fe(ImPFur)_2]^+$ (right).

S10. Emission Spectra

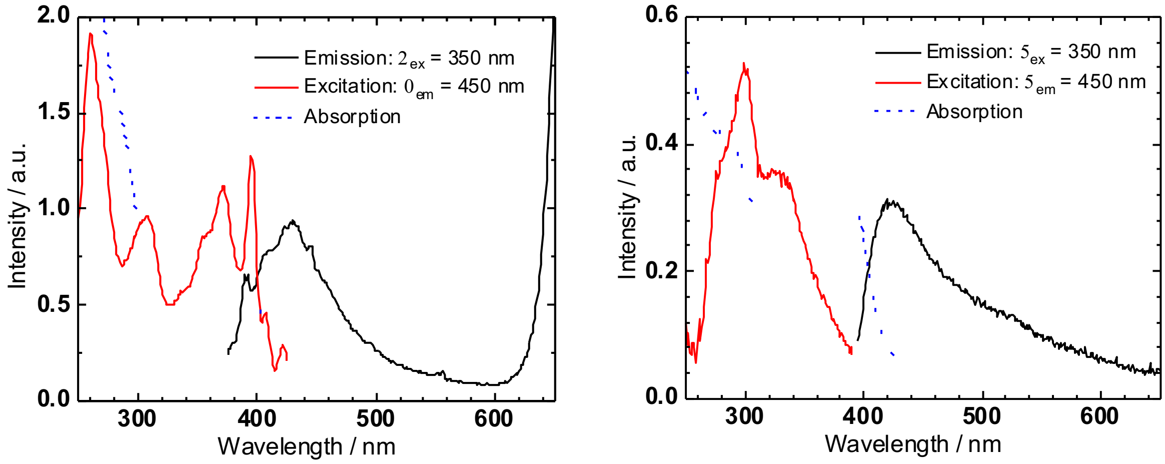

Figure S57. Emission and excitation spectra for the faint higher-energy emission observed with solutions of $[Fe^{III}(ImPBr)_2]^+$ (left) and $[Fe^{III}(ImPFur)_2]^+$ (right) upon UV excitation shown together with the scaled absorption spectra of the complexes. (All spectra in acetonitrile at r.t.)

S10. Quantum Chemical Calculations

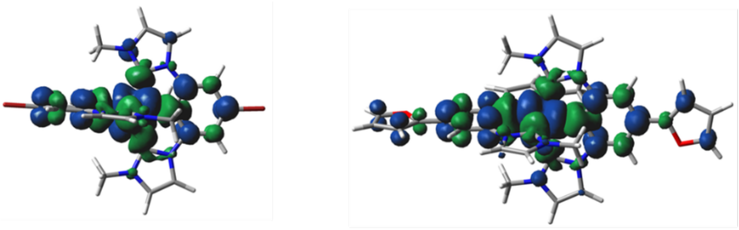

$[Fe^{III}(ImPBr)_2]PF_6$ $[Fe^{III}(ImPFur)_2]PF_6$

Figure S58: DFT optimized geometries with spin density contours of $[Fe^{III}(ImPBr)_2]PF_6$ and $[Fe^{III}(ImPFur)_2]PF_6$ on the doublet PES.

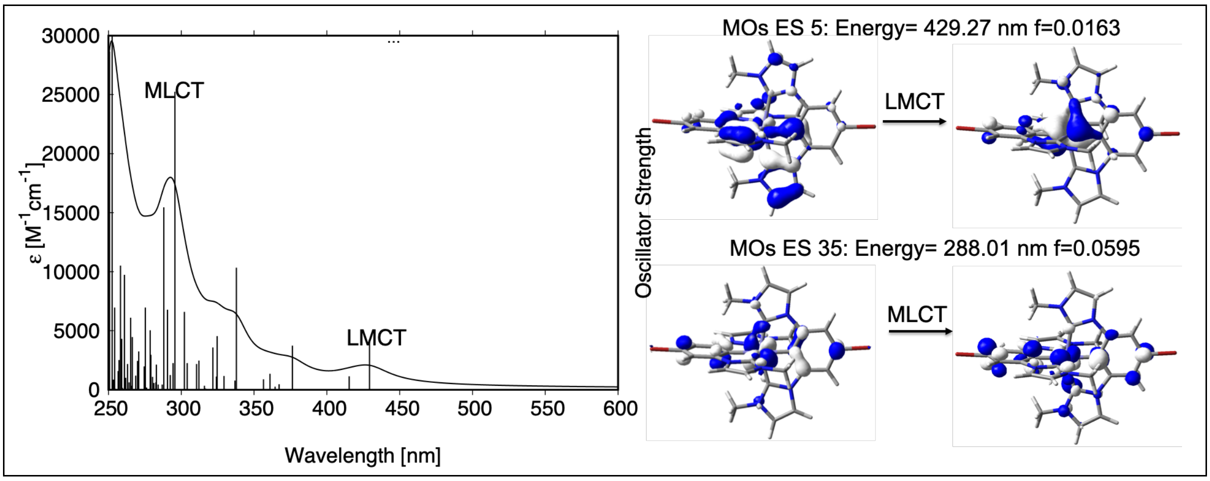

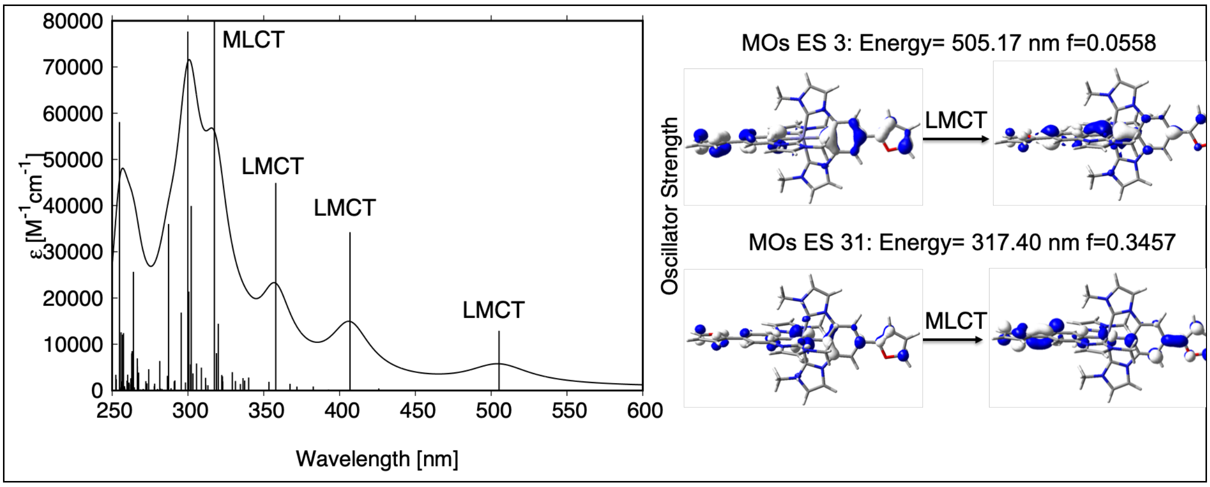

Figure S59. Top: $[Fe^{III}(ImPBr)_2]^+$ and Bottom $Fe^{III}(ImPFur)_2]^+$: Calculated absorption spectra and electronic transitions (MLCT and LMCT bands) obtained by TDDFT in acetonitrile.

The calculated potential energy curves (PECs) for the relevant electronic states of both complexes are shown in Figure S58. PECs of all complexes confirm the doublet ground state (^{2}GS) as the most stable electronic state. The metal-centered states, ^{4,6}MC, are calculated to be high in energy at the optimized doublet geometry, with the relaxed minima states displaced to longer Fe-C bond lengths. This destabilization of the metal-centered states is indicative of the strong σ-donating capabilities of the ligands. Based on the PECs, both complexes should exhibit similar excited-state dynamics, with the initial excitation populating a ^{2}LMCT state. Note that changing Br groups into furan groups significantly lowers the ^{2}LMCT state energy (by ~ 0.44 eV), thus lowering the energy gap between the ^{2}LMCT state and the ^{2}GS state. According to the energy gap law, this suggests a longer ^{2}LMCT lifetime for complex $[Fe^{III}(ImPBr)_2]^+$ than for complex $[Fe^{III}(ImPFur)_2]^+$, in agreement with the experimental data.

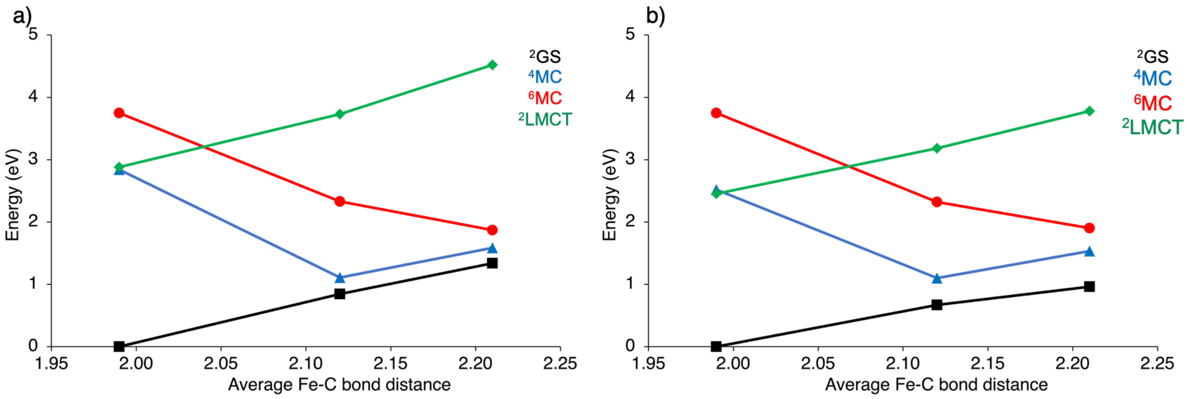

Figure S60. Potential energy curves for the relevant electronic states of a) complex $[Fe^{III}(ImPBr)_2]PF_6$ and b) complex $[Fe^{III}(ImPFur)_2]PF_6$ from energies obtained at the ^{2}GS (R = 1.99 Å), ^{4}MC (R = 2.12 Å), and ^{6}MC (R = 2.21 Å) optimized structures from single point energy calculations at the DFT (^{2}GS, ^{4}MC, ^{6}MC) and TD-DFT levels of theory (^{2}LMCT) utilizing ^{2}GS as the reference state. The reaction coordinate is given as the average Fe-C bond lengths at each optimized structure.

Table S8: List of experimental and DFT calculated bond lengths (in Å) for $[Fe^{III}(ImPBr)_2]PF_6$ (**1**).

	Experimental	Calculated		
Bond lengths		^{2}GS	^{4}MC	^{6}MC
Fe-C1	1.98	2.02	2.25	2.25
Fe-C2	2.01	2.1.99	2.25	2.25
Fe-C3	1.94	1.96	1.97	2.13
Fe-C4	1.94	1.96	2.01	2.13
Fe-C5	1.98	2.02	2.11	2.25
Fe-C6	2.01	1.99	2.11	2.25
Average Fe-C	1.98	1.99	2.12	2.21

Table S9: List of experimental and DFT calculated bond lengths (in Å) for $[Fe^{III}(ImPFur)_2]PF_6$ (**2**).

	Experimental	Calculated		
Bond lengths		^{2}GS	^{4}MC	^{6}MC
Fe-C1	1.99	2.00	2.11	2.25
Fe-C2	1.99	2.01	2.11	2.25
Fe-C3	1.94	1.95	2.01	2.13
Fe-C4	1.95	1.95	1.97	2.21
Fe-C5	1.98	2.00	2.25	2.21
Fe-C6	2.00	2.01	2.25	2.21
Average Fe-C	1.98	1.99	2.12	2.21

S11. Photoredox Catalysis Reactions

Photocatalytic C-H Arylation of 4-methoxyphenyl diazonium tetrafluoroborate

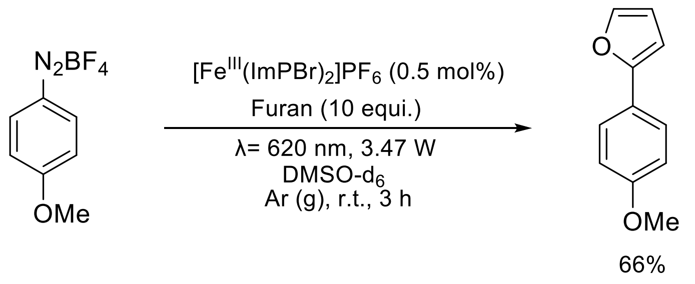

4-methoxyphenyl diazonium tetrafluoroborate (11.1 mg, 0.05 mmol), $[Fe(ImPBr)_2]PF_6$ (0.2 mg, 0.5 mol%), furan (31 µl, 0.5 mmol), and 1,3,5 trimethoxybenzene (8.41 mg, 0.05 mmol) as internal standard were charged in an NMR tube containing 0.5 ml deaerated dmso-d_6. The NMR tube was placed in front of the lamp and irradiated by red LED (623 nm) directly for 3 h for full conversion to 2-(4-methoxyphenyl)furan which was observed by ^{1}H NMR spectra.

The signal at 4.1 ppm diminished over time, indicating the consumption of 4-methoxyphenyl diazonium tetrafluoroborate and the signal at 3.78 ppm appeared indicating the formation of 2-(4-methoxyphenyl)furan after 3 h of the irradiation. Yields were referenced to the internal standard 1,3,5 trimethoxybenzene which shows a signal at 3.7 ppm.

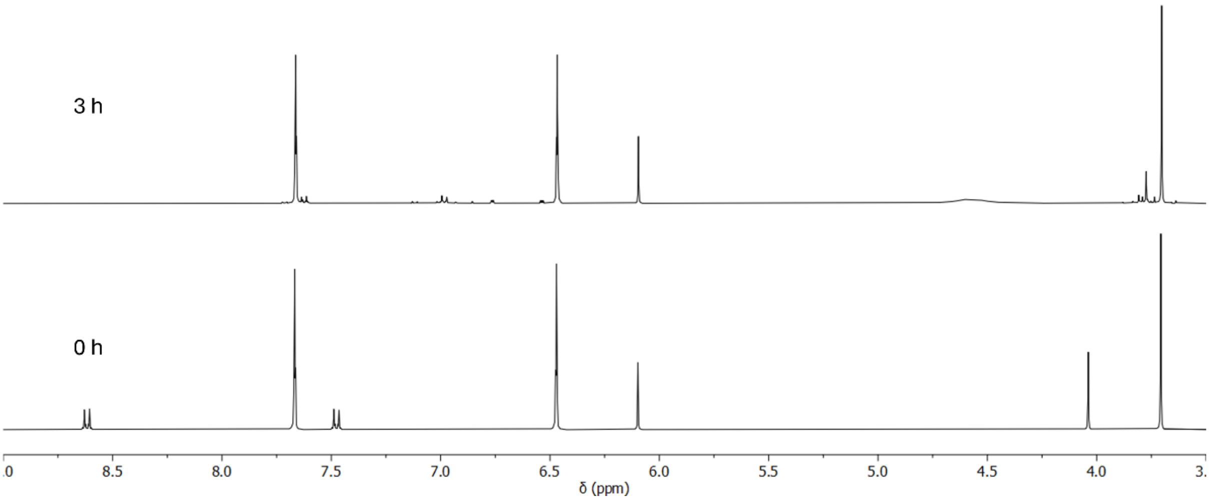

Figure S61: ^{1}H NMR spectrum of the arylation reaction proceeded with the photocatalyst $[Fe(ImPBr)_2]PF_6$ resulted into 100 % conversion and 66 % yield.

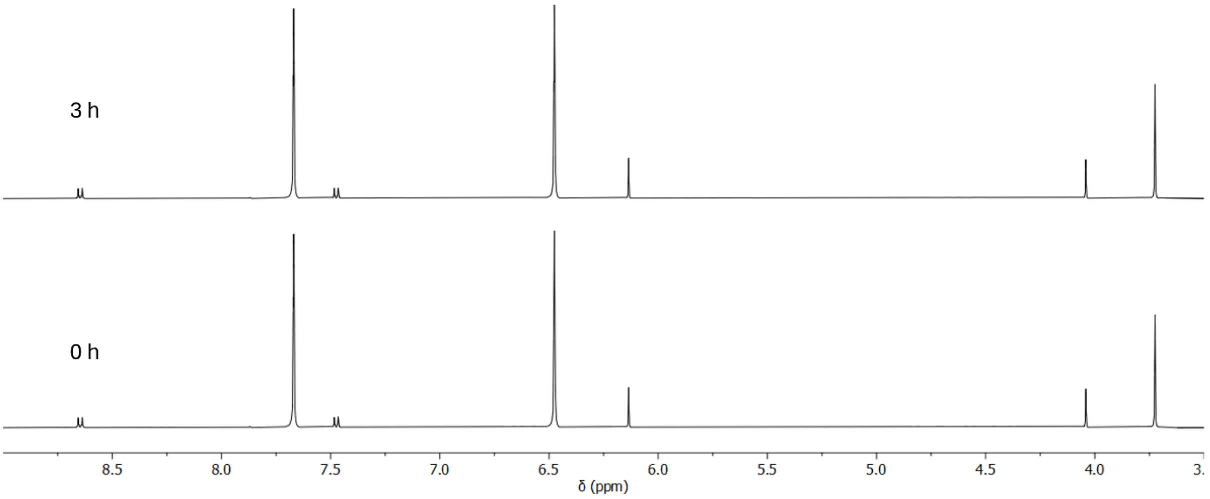

Figure S62: ^{1}H NMR spectrum of the arylation reaction proceeded without the photocatalyst.

Table S10. Conversions and yields in the photocatalytic C-H Arylation of 4-methoxyphenyl diazonium tetrafluoroborate. The reactions were run in parallel under identical reaction conditions.

**Complex**	**Conversion (%)**	**Yield (%)**
$[Fe^{III}(ImPBr)_2]PF_6$	100	66

$[Fe^{III}(ImP)_2]PF_6$	100	66
$[Fe^{III}(ImPP)_2]PF_6$	100	66

ImP = bis(2,6-bis(3-methylimidazol-2-ylidene-1-yl)phenylene), ImPP = bis(2,6-bis(3-methylimidazol-2-ylidene-1-yl)biphenylene)

Aerobic hydroxylation of Phenyl boronic acid

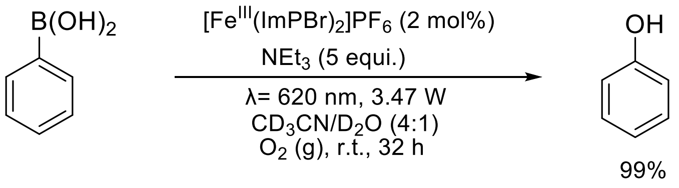

Phenyl boronic acid (3 mg, 25 µmol), triethylamine (17.3 µl, 125 µmol), $[Fe(ImPBr)_2]PF_6$ (0.42 mg, 2 mol%) and trimethyl(phenyl)silane (4.32 µl, 25µmol) as internal standard were charged in an NMR tube containing 0.5 ml CD_3CN/D_2O (4:1) and purged with O_2 for 5 minutes. The NMR tube was placed in front of the lamp and irradiated by red LED (623 nm) directly. ^{1}H NMR spectra were recorded after 16 h and 32 h and confirming the full conversion after 32 h.

The signal at 7.7 ppm diminished over time indicating the consumption of phenyl boronic acid and the signal at 7.15 ppm and 6.75 ppm appeared indicating the formation of phenol after 32 h of the irradiation. Yields were referenced to the internal standard trimethyl(phenyl)silane which shows a signal at 7.5 ppm.

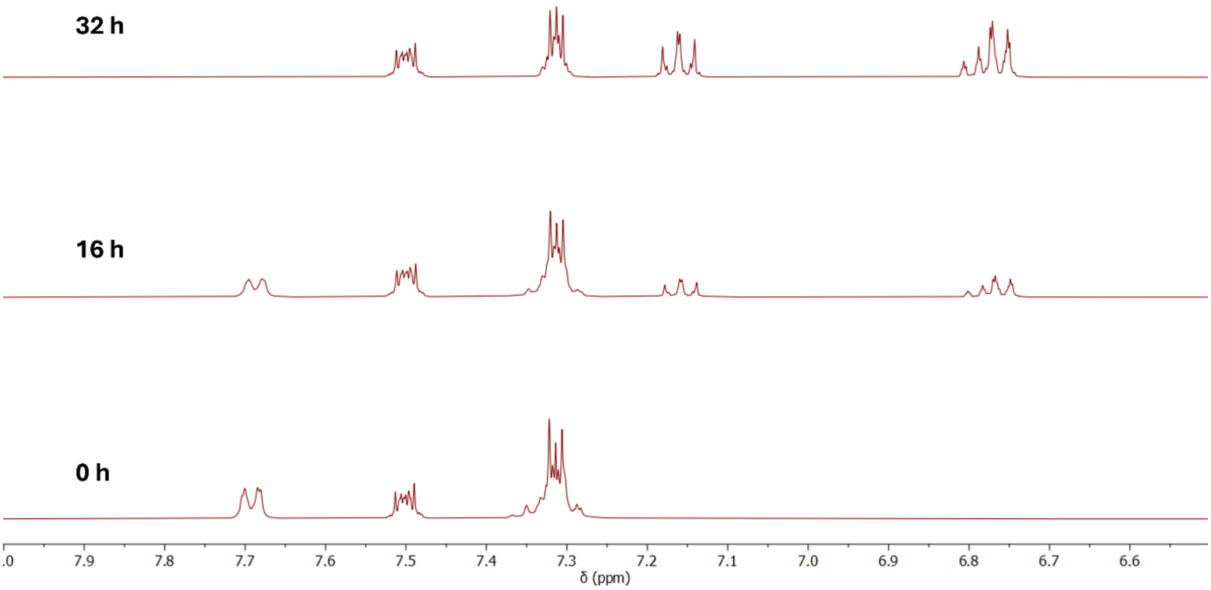

Figure S63: ^{1}H NMR spectrum of the aerobic hydroxylation reaction proceeded with the photocatalyst $[Fe(ImPBr)_2]PF_6$ resulted into 100 % conversion and 99 % yield.

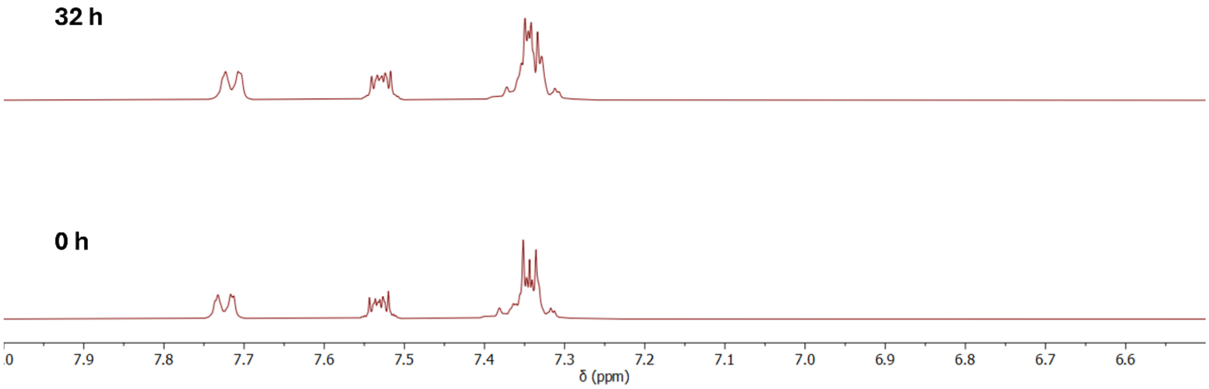

Figure S64: ^{1}H NMR spectrum of the aerobic hydroxylation reaction proceeded without the photocatalyst.

Table S11. Conversions and yields in the photocatalytic aerobic hydroxylation of phenyl boronic acid. The reactions were run in parallel under identical reaction conditions.

**Complex**	**Conversion (%)**	**Yield (%)**
$[Fe^{III}(ImPBr)_2]PF_6$	100	99
$[Fe^{III}(ImP)_2]PF_6$	100	99
$[Fe^{III}(ImPP)_2]PF_6$	100	99

ImP = bis(2,6-bis(3-methylimidazol-2-ylidene-1-yl)phenylene), ImPP = bis(2,6-bis(3-methylimidazol-2-ylidene-1-yl)biphenylene)

S11. References

(1) J. J. Snellenburg, S. P. Laptenok, R. Seger, K. M. Mullen, I. H. M. Stokkum, van. Glotaran: A Java-Based Graphical User Interface for the R Package TIMP. *J. Stat. Softw.* **2012**, 49 (3), 1–22.

(2) M. J. Frisch, G. W. Trucks, H. B. Schlegel, G. E. Scuseria, M. A. Robb, J. R. Cheeseman, G. Scalmani, V. Barone, G. A. Petersson, H. Nakatsuji, X. Li, M. Caricato, A. V. Marenich, J. Bloino, B. G. Janesko, R. Gomperts, B. Mennucci, H. P. Hratchian, J. V. Ortiz, A. F. Izmaylov, J. L. Sonnenberg, D. Williams-Young, F. Ding, F. Lipparini, F. Egidi, J. Goings, B. Peng, A. Petrone, T. Henderson, D. Ranasinghe, V. G. Zakrzewski, J. Gao, N. Rega, G. Zheng, W. Liang, M. Hada, M. Ehara, K. Toyota, R. Fukuda, J. Hasegawa, M. Ishida, T. Nakajima, Y. Honda, O. Kitao, H. Nakai, T. Vreven, K. Throssell, J. A., Jr. Montgomery, J. E. Peralta, F. Ogliaro, M. J. Bearpark, J. J. Heyd, E. N. Brothers, K. N. Kudin, V. N. Staroverov, T. A. Keith, R. Kobayashi, J. Normand, K. Raghavachari, A. P. Rendell, J. C. Burant, S. S. Iyengar, J. Tomasi, M. Cossi, J. M. Millam, M. Klene, C. Adamo, R. Cammi, J. W. Ochterski, R. L. Martin, K. Morokuma, O. Farkas, J. B. Foresman, D. J. Fox *Gaussian 16, Revision A.03. Gaussian, Inc.: Wallingford, CT* **2016**.

(3) A. D. Becke Density-Functional Exchange-Energy Approximation with Correct Asymptotic Behavior. *Phys. Rev. A* **1988**, 38 (6), 3098–3100.

(4) A. D. Becke A New Mixing of Hartree–Fock and Local Density-Functional Theories. *J. Chem. Phys.* **1993**, 98 (2), 1372–1377.

(5) A. D. Becke Density-Functional Thermochemistry. III. The Role of Exact Exchange. *J. Chem. Phys.* **1993**, 98 (7), 5648–5652.

(6) S. Grimme Semiempirical GGA-Type Density Functional Constructed with a Long-Range Dispersion Correction. *J. Comput. Chem.* **2006**, 27 (15), 1787–1799.

(7) G. Scalmani, M. J. Frisch Continuous Surface Charge Polarizable Continuum Models of Solvation. I. General Formalism. *J. Chem. Phys.* **2010**, 132 (11), 114110.

(8) A. D. McLean, G. S. Chandler Contracted Gaussian Basis Sets for Molecular Calculations. I. Second Row Atoms, Z =11–18. *J. Chem. Phys.* **1980**, 72 (10), 5639–5648.

(9) M. Dolg, U. Wedig, H. Stoll, H. Preuss Energy‐adjusted Ab Initio Pseudopotentials for the First Row Transition Elements. *J. Chem. Phys.* **1987**, 86 (2), 866‐872.

(10) P.-O. Löwdin Quantum Theory of Many-Particle Systems. I. Physical Interpretations by Means of Density Matrices, Natural Spin-Orbitals, and Convergence Problems in the Method of Configurational Interaction. *Phys. Rev.* **1955**, 97 (6), 1474–1489.

(11) S. I. Gorelsky *AOMix: Program for Molecular Orbital Analysis*; version 6.94. http://www.sg-chem.net/.

(12) M. E. Casida Time-Dependent Density Functional Response Theory for Molecules. *In Recent Advances in Density Functional Methods*; **1995**; pp 155–192.

(13) M. E. Casida, C. Jamorski, K. C. Casida, D. R. Salahub Molecular Excitation Energies to High-Lying Bound States from Time-Dependent Density-Functional Response Theory: Characterization and Correction of the Time-Dependent Local Density Approximation Ionization Threshold. *J. Chem. Phys.* **1998**, 108 (11), 4439–4449.

(14) R. L. Martin Natural Transition Orbitals. *J. Chem. Phys.* **2003**, 118 (11), 4775–4777.

(15) G. M. Sheldrick Crystal Structure Refinement with SHELXL. *Acta Crystallogr. C* **2015**, 71 (1), 3–8.

(16) G. M. Sheldrick A Short History of SHELX. *Acta Crystallogr. A* **2008**, 64 (1), 112–122.

(17) O. Prakash, L. Lindh, N. Kaul, N. W. Rosemann, I. B. Losada, C. Johnson, P. Chábera, A. Ilic, J. Schwarz, A. K. Gupta, J. Uhlig, T. Ericsson, L. Häggström, P. Huang, J. Bendix, D. Strand, A. Yartsev, R. Lomoth, P. Persson, K. Wärnmark Photophysical Integrity of the Iron(III) Scorpionate Framework in Iron(III)–NHC Complexes with Long-Lived 2LMCT Excited States. *Inorg. Chem.* **2022**, 61 (44), 17515–17526.

(18) K. S. Kjær, N. Kaul, O. Prakash, P. Chábera, N. W. Rosemann, A. Honarfar, O. Gordivska, L. A. Fredin, K.-E. Bergquist, L. Häggström, T. Ericsson, L. Lindh, A. Yartsev, S. Styring, P. Huang, J. Uhlig, J. Bendix, D.

Strand, V. Sundström, P. Persson, R. Lomoth, K. Wärnmark Luminescence and reactivity of a charge-transfer excited iron complex with nanosecond lifetime. *Science* **2019**, 363 (6424), 249–253.

(19) P. Chábera, Y. Liu, O. Prakash, E. Thyrhaug, A. El Nahhas, A. Honarfar, S. Essén, L. A. Fredin, T. C. B. Harlang, K. S. Kjær, K. Handrup, F. Ericson, H. Tatsuno, K. Morgan, J. Schnadt, L. Häggström, T. Ericsson, A. Sobkowiak, S. Lidin, P. Huang, S. Styring, J. Uhlig, J. Bendix, R. Lomoth, V. Sundström, P. Persson, K. Wärnmark A Low-Spin Fe(III) Complex with 100-Ps Ligand-to-Metal Charge Transfer Photoluminescence. *Nature* **2017**, 543 (7647), 695–699.

(20) O. Prakash, P. Chábera, N. W. Rosemann, P. Huang, L. Häggström, T. Ericsson, D. Strand, P. Persson, J. Bendix, R. Lomoth, K. Wärnmark A Stable Homoleptic Organometallic Iron(IV) Complex. *Chem. Eur. J.* **2020**, 26 (56), 12728–12732.

(21) P. Gütlich, E. Bill, A. X. Trautwein Mössbauer Spectroscopy and Transition Metal Chemistry; Springer Berlin Heidelberg: Berlin, Heidelberg, **2011**.

(22) R. Ingalls Electric-Field Gradient Tensor in Ferrous Compounds. *Phys. Rev.* **1964**, 133 (3A), A787–A795.
